# Novel thienopyrimidine derivatives as dual EGFR and VEGFR-2 inhibitors: design, synthesis, anticancer activity and effect on cell cycle profile

**DOI:** 10.1080/14756366.2019.1593160

**Published:** 2019-03-28

**Authors:** Aml E.-S. Mghwary, Ehab M. Gedawy, Aliaa M. Kamal, Suzan M. Abuel-Maaty

**Affiliations:** a Department of Pharmaceutical Organic Chemistry, Faculty of Pharmacy, Cairo University, Cairo, Egypt;; b Department of Pharmaceutical Chemistry, Faculty of Pharmacy and Pharmaceutical Industries, Badr University in Cairo BUC, Cairo, Egypt;; c Department of Organic Chemistry, Faculty of Pharmacy, October University for Modern Science and Arts (MSA), Cairo, Egypt

**Keywords:** Thieno[2,3-d]pyrimidines, design, synthesis, anticancer activity, EGFR, VEGFR-2, vandetanib, apoptosis

## Abstract

**Aim:** Design and synthesis of thienopyrimidine derivatives as dual EGFR and VEGFR-2 inhibitors.**Material and methods:** A series of novel 6,7,8,9-tetrahydro-5*H*-cyclohepta[4,5]thieno[2,3-*d*]pyrimidine derivatives with different substituents on C-4 position was synthesized and evaluated for their anticancer activity against MCF-7 cell line. EGFR, VEGFR-2 inhibitory assay, the cell cycle analysis and apoptosis induction ability of the most potent compound **5f** were evaluated.**Results:** Most of the compounds showed moderate to significant anticancer activity. Compound **5f** exhibited the most potent anticancer activity being 1.73- and 4.64-folds more potent than erlotinib and doxorubicin, respectively. Compound **5f** showed potent EGFR inhibitory activity being 1.18-folds more potent than reference standard erlotinib and it also showed good VEGFR-2 inhibitory activity at the micromolar level with IC_50_ value 1.23 µM. Compound **5f** caused induction of cell cycle arrest at G2/M phase and accumulation of cells in pre-G1 phase. Compound **5f** induced cellular apoptosis.

## Introduction

**Figure 1.**
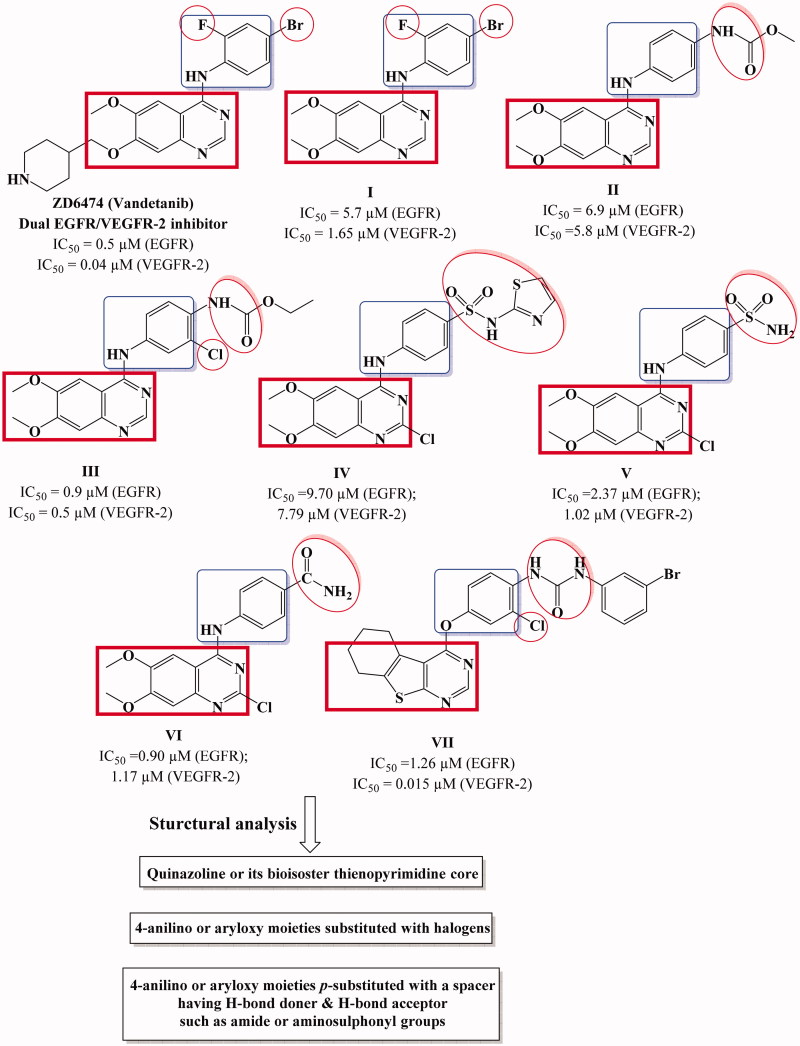
Structures of potent dual EGFR and VEGFR-2 inhibitors.

Protein kinases have a crucial role in signal transduction pathways that mediate several cellular functions^1^. Protein kinase inhibitors has received great attention in the last decades due to their ongoing role in fighting cancer^2^. The epidermal growth factor receptor (EGFR) is a tyrosine kinase transmembrane receptor that mediates several signal transduction cascades [Ras/MAPK, PI3K/Akt, Jak/STAT] which regulate cell proliferation, growth and apoptosis^3^. EGFR over-expression is implicated in various types of cancer such as breast, colon, ovarian and prostate through enhancing the cancer cell proliferation, invasiveness, metastasis and angiogenesis^4–7^. Moreover, EGFR-signaling pathways stimulate vascular endothelial growth factor (VEGF)^8^ which is considered as the key inducer of tumor angiogenesis. Angiogenesis is the process of blood vessel formation that starts with dilatation and increase in the vascular permeability of the existing capillaries and venules. Followed by activation, migration and proliferation of the endothelial cells, of the blood vessels to form the new capillaries^9^. Binding of VEGF with VEGFR-2 stimulates signaling pathways (p38MAPK, PI3K/Akt) that mediate several cellular functions such as proliferation, migration, survival and vascular permeability for the tumor cell and hence promotes angiogenesis^10^. Furthermore, it has been demonstrated that VEGFR-2 are highly expressed in cancer cells especially endothelial cells^11^. In addition, both glycoproteins, EGFR and VEGFR-2 are closely related; inhibition of EGFR decreasing the expression of VEGF, whereas targeting of VEGFR-2 potentiate the anticancer activity of EGFR inhibitors^12–14^. Therefore, recent therapies based on dual inhibition of both EGFR and VEGFR-2 represents a promising cancer treatment protocol^15^^,^^16^. Recently, several EGFR/VEGFR-2 dual inhibitors have been discovered^14^^,^^17–19^, such as vandetanib (ZD6474, Caprelsa^®^, Figure 1) that exhibited potent inhibitory activity against both EGFR and VEGFR-2 (with IC_50_=0.50, 0.04 µM, respectively)^20^. Vandetanib had FDA approval in 2011 for treatment of thyroid cancer^21^. In addition, such 4-anilinoquinazoline derivative substituted with halogen atoms in position 2 and 4 of the anilino group showed significant anticancer activity against breast, colorectal and lung cancers^22–26^.

In 2010, Garofalo et al.^18^ designed several 4-anilinoquinazoline derivatives with urea or carbamate ester moieties (**I**–**III**) (Figure 1). It was reported that presence of urea entity enhanced the VEGFR-2 inhibitory activity and abolished the activity against EGFR. On the other hand, derivatives with carbamate ester group (compound **II)** showed promising dual EGFR/VEGFR-2 inhibitory activity^18^. Furthermore, the presence H-donor/acceptor group at the para position of the 4-anilino moiety such as sulfonamide or amide group (compound **IV–VI)** improved the binding to both EGFR and VEGFR-2 receptors and as a result enhanced the dual enzyme inhibitory activity^19^^,^^27^. Finally, presence of halide in the phenyl ring of 4-anilino or aryloxy moiety enhance the dual inhibitory activity (compound **III**, **VII**) (Figure 1)^14^^,^^28^.

EGFR inhibitors differ in their structure but have common pharmacophoric features, the first pharmacophore is a flat aromatic heterocyclic fused system that acts as hydrogen bond acceptor (HBA) to reside in adenine binding pocket (hinge segment). Moreover, EGFR inhibitors should have a hydrophobic moiety such as phenyl ring (hydrophobic head) linked to aromatic heterocyclic fused system with NH spacer to occupy the hydrophobic region I of the enzyme. Finally, another hydrophobic moiety that is attached or fused to the aromatic flat, (hydrophobic tail) to occupy hydrophobic region I^29^.

On the other hand, the structural requirements for VEGFR-2 kinase inhibitory activity is a flat aromatic ring system with a hydrogen bond acceptor group to interact with NH of Cys917. In addition to the presence of another hydrophobic group with another hydrogen bond acceptor group as well as a hydrogen bond donor group (such as ureido or amide moiety) to interact with NH of Asp1044 and C=O of Glu883, respectively^30^.

**Figure 2.**
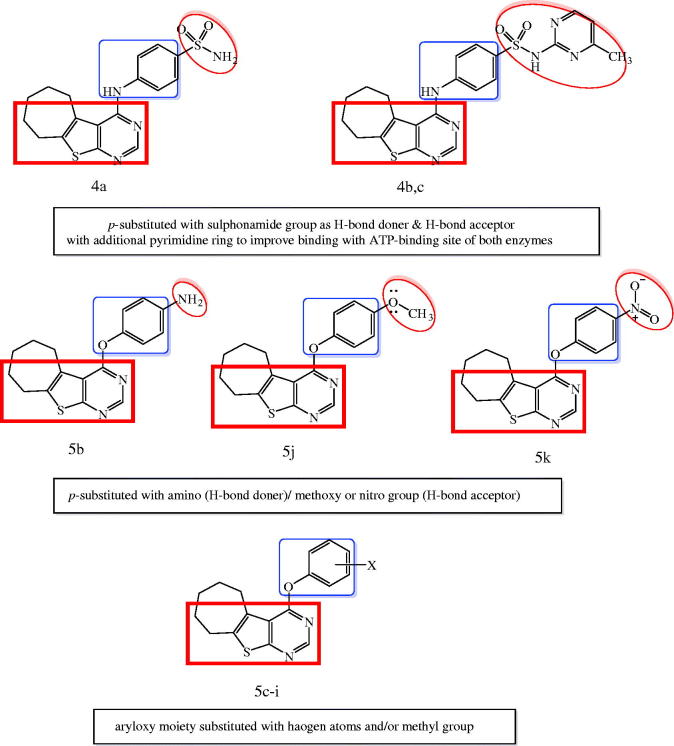
Design strategy of the targeted thieno[2,3-*d*]pyrimidines derivatives as dual EGFR and VEGFR-2 inhibitors.

Motivated by these facts, herein, we designed and synthesized novel cyclohepta[4,5]thieno[2,3-*d*]pyrimidine derivatives as dual EGFR and VEGFR-2 inhibitors (Figure 2). The structural modifications involved replacement of 4-anilinoquinazoline scaffold with is their bioisostere thieno[2,3-*d*]pyrimidine core fused to large lipophilic cycloalkyl group guided by their reported potent anticancer activity^31^^,^^32^. In addition, we explored the substitution of the phenyl ring of 4-anilino or 4-aryloxy moieties with, halogen, hydrogen bond donor (such as amino group), hydrogen bond acceptor (nitro, methoxy groups) or hydrogen bond donor/acceptor (sulphonylamino group) to substantiate the effect of such variations on the dual EGFR and VEGFR-2 inhibitory activity as well as their anticancer activity against MCF-7 breast cancer cell line. The cytotoxicity of the most potent compounds was estimated on MCF-10A (the human mammary epithelial cell line) to determine their safety to normal cells.

## Material and methods

Melting points were determined on Stuart SMP10 apparatus and the values were uncorrected. IR spectra were carried out using KBr discs on Shimadzu IR spectrophotometer (FTIR, 8400S, Kyoto, Japan), Faculty of Pharmacy, Cairo University, Egypt and values were represented in cm^−1^. ^1^H NMR spectra were carried out using a Bruker Advance 400 MHz NMR (Bruker Corp., Billerica, MA) spectrometer, Microanalytical Unit, Faculty of Pharmacy, Cairo University, Egypt. Chemical shift values were recorded in ppm on *δ* scale using tetramethylsilane (TMS) as internal standard and coupling constants (J) were given in Hz. ^13^C NMR spectra were carried out using a Bruker Advance 100 MHz spectrometer, Microanalytical Unit, Faculty of Pharmacy, Cairo University, Egypt. Microanalyses for C, H and N were carried out at the Regional Center for Mycology and Biotechnology, Faculty of Pharmacy, Al-Azhar University. Mass spectra were recorded on a GCMP-QP1000 EX Mass spectrometer. Progress of the reactions was monitored by TLC using aluminum sheets precoated with UV fluorescent silica gel (Merck 60 F254, Kenilworth, NJ) and spots were visualized by UV lamp. The solvent system used was chloroform:benzene:methanol [4.5:5:0.5] and few drops of TEA. Chemicals were purchased from Acros Organics, Geel, Belgium and Sigma-Aldrich, St. Louis, MO. The cytotoxic activity, the cell cycle, *in vitro* EGFR and VEGFR-2 inhibitory activities assays were evaluated at the Confirmatory Unit, Vaccines and Sera Co. (VACSERA), Egypt.

## Chemistry

*2-Amino-5,6,7,8-tetrahydro-4H-cyclohepta[b]thiophene-3-carbonitrile****(1)****, 6,7,8,9-tetrahydro-3H-cyclohepta[4,5]thieno[2,3-d]pyrimidin-4(5H)-one***(2)** and *4-chloro-6,7,8,9-tetrahydro-5H-cyclohepta[4,5]thieno[2,3-d]pyrimidine****(3)***^33^ were prepared according to reported procedures.

***General procedure for the preparation of N-substituted-4-[(6,7,8,9-tetrahydro-5H-cyclohepta[4,5]thieno[2,3-d]pyrimidin-4-yl)amino]benzenesulfonamides (4a–c).*** A mixture of 4-chloro-6,7,8,9-tetrahydro-5*H*-cyclohepta[4,5]thieno[2,3-*d*]pyrimidine **(3)** (1.50 g, 0.0063 mol) and the appropriate substituted benzenesulfonamide (0.0063 mol) in isopropanol (15 mL) was heated under reflux for 10 h. The reaction mixture was allowed to cool, the solid formed was filtered, dried and crystallized from absolute ethanol to give compounds **4a–c**.

***4-((6,7,8,9-Tetrahydro-5H-cyclohepta[4,5]thieno[2,3-d]pyrimidin-4-yl)amino)benzenesulfonamide* (4a)**. Yield: 86%; m.p.: 271–272 °C; IR (KBr) *v*_max_: 3414, 3360, 3290 (NH, NH_2_), 3082 (C–H aromatic), 2916, 2846 (C–H aliphatic) cm^−1^; ^1^H NMR (400 MHz, DMSO-d_6_): *δ* 1.70–1.74 (m, 4H, 2CH_2_), 1.86–1.87 (m, 2H, CH_2_), 2.93–2.96 (m, 2H, CH_2_), 3.16–3.18 (m, 2H, CH_2_), 7.22 (br.s, 2H, NH_2_, D_2_O exchangeable), 7.72 (d, 2H, *J* = 8.8 Hz, ArH), 7.78 (d, 2H, *J* = 8.8 Hz, ArH), 8.47 (s, H, C_2_–H), 9.05 (s, 1H, NH, D_2_O exchangeable) ppm; ^13^C NMR (100 MHz, DMSO-d_6_): *δ* 27.0, 27.1, 29.2, 29.3, 31.1, 118.9, 119.4, 121.6, 126.8, 132.6, 138.4, 143.2, 151.3, 154.9, 164.3 ppm; EIMS [*m/z*, %]: 374 [M·^+^, 45.91], 129 [100.00]; Anal. Calcd for C_17_H_18_N_4_O_2_S_2_ (374.48): C, 54.52; H, 4.84; N, 14.96. Found: C, 54.89; H, 4.97; N, 15.31.

***N-(Pyrimidin-2-yl)-4-((6,7,8,9-tetrahydro-5H-cyclohepta[4,5]thieno[2,3-d]pyrimidin-4-yl)amino)benzenesulfonamide* (4b)**. Yield: 77%; m.p.: 243–245 °C; IR (KBr) *v*_max_: 3456, 3371 (2 NH), 3101, 3078, 3035 (C–H aromatic), 2927, 2850 (C–H aliphatic) cm^−1^; ^1^H NMR (400 MHz, DMSO-d_6_): *δ* 1.64–1.72 (m, 4H, 2CH_2_), 1.82–1.84 (m, 2H, CH_2_), 2.92–2.94 (m, 2H, CH_2_), 3.11–3.14 (m, 2H, CH_2_), 6.00 (s, 1H, NH, D_2_O exchangeable), 6.57 (d, 2H, *J* = 8.8 Hz, ArH), 7.01 (t, 1H, *J* = 4.8 Hz, ArH), 7.62 (d, 2H, *J* = 8.8 Hz, ArH), 8.47 (s, 1H, C2-H), 8.52 (d, 2H, *J* = 4.8 Hz, ArH), 9.07 (s, 1H, NH, D_2_O exchangeable); ^13^C NMR (100 MHz, DMSO-d6): *δ* 27.0, 27.1, 29.1, 29.4, 31.1, 112.6, 115.9, 120.4, 125.3, 129.0, 130.2, 132.5, 138.5, 151.8, 153.4, 157.4, 157.6, 158.7 ppm; EIMS [*m/z*, %]: 452 [M·^+^, 8.09], 133 [100.00]; Anal. Calcd for C_21_H_20_N_6_O_2_S_2_ (452.55): C, 55.73; H, 4.45; N, 18.57. Found: C, 55.61; H, 4.62; N, 18.74.

***N-(4-Methylpyrimidin-2-yl)-4-((6,7,8,9-tetrahydro-5H-cyclohepta[4,5]thieno[2,3-d]pyrimidin-4-yl)amino)benzenesulfonamide* (4c)**. Yield: 77%; m.p. 260–262 °C; IR (KBr) *v*_max_: 3437, 3228 (2 NH), 3070, 3035 (C–H aromatic), 2920, 2850 (C–H aliphatic) cm^−1^; ^1^H NMR (400 MHz, DMSO-d_6_): *δ* 1.64–1.70 (m, 2H, CH_2_),1.72–1.77 (m, 2H, CH_2_), 1.82–1.83 (m, 2H, CH_2_), 2.33 (s, 3H, CH_3_), 2.91–2.93 (m, 2H, CH_2_), 3.11–3.14 (m, 2H, CH_2_), 4.77 (s, 1H, NH, D_2_O exchangeable), 6.91 (d, 1H, *J* = 8.8 Hz, ArH), 7.72 (d, 2H, *J* = 8.8 Hz, ArH), 7.95 (d, 2H, *J* = 8.8 Hz, ArH), 8.34 (d, 1H, *J* = 8.8 Hz, ArH), 8.47 (s, 1H, C2-H), 9.11(s, 1H, NH, D_2_O exchangeable); ^13^C NMR (100 MHz, DMSO-d6): *δ* 23.7, 27.0, 27.1, 29.1, 29.3, 31.1, 115.3, 119.7, 120.5, 129.2, 132.5, 133.9, 138.5, 144.4, 151.5, 154.5, 157.1, 158.0, 165.1, 168.7 ppm; EIMS [*m/z*, %]: 466 [M·^+^, 27.71], 70 [100.00]; Anal. Calcd for C_22_H_22_N_6_O_2_S_2_ (466.58): C, 56.63; H, 4.75; N, 18.01. Found: C, 56.87; H, 4.89; N, 18.25.

***General procedure for the preparation of 4-(aryloxy)-6,7,8,9-tetrahydro-5H-cyclohepta[4,5]thieno[2,3-d]pyrimidines (5a-k).*** A solution of 4-chloro-6,7,8,9-tetrahydro-5*H*-cyclohepta[4,5]thieno[2,3-*d*]pyrimidine **(3)** (1.90 g, 0.0080 mol) and phenolic compound (0.0096 mol) in alcoholic potassium hydroxide solution (KOH 0.54 g in 20 mL ethanol) was heated under reflux for 20 h. The reaction mixture was allowed to cool to room temperature, the solid formed was filtered, dried and crystallized from absolute ethanol to afford compounds **5a–k**.

***3-((6,7,8,9-Tetrahydro-5H-cyclohepta[4,5]thieno[2,3-d]pyrimidin-4-yl)oxy)aniline (5a).*** Yield: 61%; m.p.: 179–180 °C; IR (KBr) *v*_max_: 3452, 3325 (NH_2_), 3109, 3055 (C–H aromatic), 2916, 2846 (C–H aliphatic) cm^−1^; ^1^H NMR (400 MHz, DMSO-d_6_): *δ* 1.68–1.72 (m, 4H, 2CH_2_), 1.87–1.88 (m, 2H, CH_2_), 2.94–2.97 (m, 2H, CH_2_), 3.24–3.26 (m, 2H, CH_2_), 5.28 (s, 2H, NH_2_, D_2_O exchangeable), 6.34 (dd, 1H, *J* = 8 Hz, 2 Hz, ArH), 6.39 (t, 1H, *J* = 2 Hz, ArH), 6.48 (dd, 1H, *J* = 8 Hz, 2 Hz, ArH), 7.06 (t, 1H, *J* = 8 Hz, ArH), 8.47 (s, 1H, C2-H) ppm; ^13^C NMR (100 MHz, DMSO-d_6_): δ 26.9, 27.3, 28.4, 29.6, 31.9, 107.4, 109.1, 111.6, 119.8, 130.1, 132.6, 140.2, 150.7, 152.1, 153.5, 163.2, 166.7 ppm; EIMS [*m/z*, %]: 312 [M + 1·^+^, 37.96], 311 [M·^+^, 100.00]; Anal. Calcd for C_17_H_17_N_3_OS (311.40): C, 65.57; H, 5.50; N, 13.49. Found: C, 65.31; H, 5.73; N, 13.68.

***4-((6,7,8,9-Tetrahydro-5H-cyclohepta[4,5]thieno[2,3-d]pyrimidin-4-yl)oxy)aniline (5b).*** Yield: 30%; m.p.>300 °C; IR (KBr) *v*_max_: 3440–3400 (NH_2_), 3030 (C–H aromatic), 2920, 2850 (C–H aliphatic), 1612 (C=N) cm^−1^; ^1^H NMR (400 MHz, DMSO-d_6_): *δ* 1.69–1.70 (m, 4H, 2CH_2_), 1.87–1.88 (m, 2H, CH_2_), 2.94–2.97 (m, 2H, CH_2_), 3.25–3.28 (m, 2H, CH_2_), 5.12 (s, 2H, NH_2_, D_2_O exchangeable), 6.61 (d, 2H, *J* = 8.8 Hz, ArH), 6.89 (d, 2H, *J* = 8.8 Hz, ArH), 8.43 (s, 1H, C2-H) ppm; ^13^C NMR (100 MHz, DMSO-d6): δ 27.0, 27.3, 28.5, 29.6, 31.9, 114.7, 122.7, 132.7, 136.9, 139.9, 142.4, 146.9, 152.1, 163.9, 166.4 ppm; EIMS [*m/z*, %]: 311 [M·^+^, 9.79], 298 [100.00]; Anal. Calcd for C_17_H_17_N_3_OS (311.40): C, 65.57; H, 5.50; N, 13.49. Found: C, 65.83; H, 5.65; N, 13.72.

***4-(4-Bromophenoxy)-6,7,8,9-tetrahydro-5H-cyclohepta[4,5]thieno[2,3-d]pyrimidine (5c).*** Yield: 73%; m.p.: 135–136 °C; IR (KBr) *v*_max_: 3101, 3066 (C–H aromatic), 2916, 2854 (C–H aliphatic) cm^−1^; ^1^H NMR (400 MHz, DMSO-d_6_): *δ* 1.70–1.72 (m, 4H, 2CH_2_), 1.88–1.90 (m, 2H, CH_2_), 2.97–3.00 (m, 2H, CH_2_), 3.25–3.28 (m, 2H, CH_2_), 7.29 (d, 2H, *J* = 8.8 Hz, ArH), 7.66 (d, 2H, *J* = 8.8 Hz, ArH), 8.48 (s, 1H, C2-H) ppm; ^13^C NMR (100 MHz, DMSO-d_6_): δ 26.9, 27.3, 28.5, 29.6, 31.8, 118.3, 119.8, 124.9, 132.5, 133.0, 140.7, 151.7, 151.8, 162.8, 166.8 ppm; Anal. Calcd for C_17_H_15_BrN_2_OS (375.28): C, 54.41; H, 4.03; N, 7.46. Found: C, 54.68; H, 4.22; N, 7.68.

***4-(2-Chlorophenoxy)-6,7,8,9-tetrahydro-5H-cyclohepta[4,5]thieno[2,3-d]pyrimidine (5d).*** Yield: 21%; m.p.>300 °C; IR (KBr) *v*_max_: 3040 (C–H aromatic), 2920, 2850 (C–H aliphatic), 1620 (C=N) cm^−1^; ^1^H NMR (400 MHz, DMSO-d_6_): *δ* 1.71–1.73 (m, 4H, 2CH_2_), 1.90–1.91 (m, 2H, CH_2_), 2.99–3.02 (m, 2H, CH_2_), 3.33–3.34 (m, 2H, CH_2_), 7.35–7.40 (m, 1H, ArH), 7.45–7.52 (m, 2H, ArH), 7.64 (d, 1H, *J* = 8 Hz, ArH), 8.48 (s, 1H, C2–H) ppm; ^13^C NMR (100 MHz, DMSO-d_6_): δ 26.9, 27.3, 28.5, 29.6, 31.8, 119.5, 125.3, 126.5, 127.9, 129.0, 130.8, 132.5, 140.9, 148.3, 151.9, 152.3, 167.0 ppm; Anal. Calcd for C_17_H_15_ClN_2_OS (330.83): C, 61.72; H, 4.57; N, 8.47. Found: C, 61.89; H, 4.66; N, 8.63.

***4-(4-Chlorophenoxy)-6,7,8,9-tetrahydro-5H-cyclohepta[4,5]thieno[2,3-d]pyrimidine (5e).*** Yield: 53%; m.p. 115–116 °C; IR (KBr) *v*_max_: 3105 (C–H aromatic), 2912, 2854 (C–H aliphatic) cm^−1^; ^1^H NMR (400 MHz, DMSO-d_6_): *δ* 1.70–1.72 (m, 4H, 2CH_2_), 1.88–1.89 (m, 2H, CH_2_), 2.96–2.99 (m, 2H, CH_2_), 3.25–3.28 (m, 2H, CH_2_), 7.34 (d, 2H, *J* = 8.8 Hz, ArH), 7.53 (d, 2H, *J* = 8.8 Hz, ArH), 8.48 (s, 1H, C2-H) ppm; ^13^C NMR (100 MHz, DMSO-d_6_): δ 26.9, 27.3, 28.5, 29.6, 31.8, 119.8, 124.5, 130.0, 130.2, 132.5, 140.5, 151.3, 151.9, 162.9, 166.9 ppm; Anal. Calcd for C_17_H_15_ClN_2_OS (330.83): C, 61.72; H, 4.57; N, 8.47. Found: C, 61.56; H, 4.71; N, 8.79.

***4-(4-Chloro-3-methylphenoxy)-6,7,8,9-tetrahydro-5H-cyclohepta[4,5]thieno[2,3-d]pyrimidine (5f).*** Yield: 23%; m.p.>300 °C; IR (KBr) *v*_max_: 3059, 3032 (C–H aromatic), 2924, 2850 (C–H aliphatic) cm^−1^; ^1^H NMR (400 MHz, DMSO-d_6_): *δ* 1.68–1.71 (m, 4H, 2CH_2_), 1.87–1.88 (m, 2H, CH_2_), 2.34 (s, 3H, CH_3_), 2.95–2.98 (m, 2H, CH_2_), 3.24–3.26 (m, 2H, CH_2_), 7.15 (dd, 1H, *J* = 8.8 Hz, *J* = 2.8 Hz, ArH), 7.31 (d, 1H, *J* = 2.8 Hz, ArH), 7.49 (d, 1H, *J* = 8.8 Hz, ArH), 8.47 (s, 1H, C2-H) ppm; ^13^C NMR (100 MHz, DMSO-d_6_): *δ* 20.0, 26.9, 27.3, 28.5, 29.6, 31.8, 119.8, 121.8, 125.1, 130.2, 130.4, 132.5, 137.5, 140.5, 151.1, 151.9, 162.9, 166.8 ppm; Anal. Calcd for C_18_H_17_ClN_2_OS (344.86): C, 62.69; H, 4.97; N, 8.12. Found: C, 62.83; H, 4.69; N, 8.35.

***4-(4-Fluorophenoxy)-6,7,8,9-tetrahydro-5H-cyclohepta[4,5]thieno[2,3-d]pyrimidine (5g).*** Yield: 22%; m.p.>300 °C; IR (KBr) *v*_ma_*_x_*: 3078, 3012 (C–H aromatic), 2920, 2850 (C–H aliphatic), 1620 (C=N) cm^−1^; ^1^H NMR (400 MHz, DMSO-d_6_): *δ* 1.72–1.75 (m, 4H, 2CH_2_), 1.87–1.90 (m, 2H, CH_2_), 2.97–3.00 (m, 2H, CH_2_), 3.26–3.29 (m, 2H, CH_2_), 7.28–7.34 (m, 4H, ArH), 8.47 (s, 1H, C2-H) ppm; ^13^C NMR (100 MHz, DMSO-d_6_): *δ* 26.9, 27.3, 28.5, 29.6, 31.8,116.6, 116.8, 119.8, 124.4, 124.5, 132.6, 136.9, 140.5, 148.5, 151.9, 163.1, 166.7 ppm; EIMS [*m/z*, %]: 314 [M·^+^, 5.12], 303 [100.00]; Anal. Calcd for C_17_H_15_FN_2_OS (314.38): C, 64.95; H, 4.81; N, 8.91. Found: C, 65.11; H, 4.89; N, 9.14.

***4-(o-Tolyloxy)-6,7,8,9-tetrahydro-5H-cyclohepta[4,5]thieno[2,3-d]pyrimidine (5h).*** Yield: 24%; m.p.>300 °C; IR (KBr) *v*_max_: 3066, 3024 (C–H aromatic), 2924, 2846 (C–H aliphatic), 1589 (C=N) cm^−1^; ^1^H NMR (400 MHz, DMSO-d_6_): *δ* 1.70–1.71 (m, 4H, 2CH_2_), 1.88–1.90 (m, 2H, CH_2_), 2.08 (s, 3H, CH_3_), 2.97–2.99 (m, 2H, CH_2_), 3.30–3.33 (m, 2H, CH_2_), 7.22 (t, 2H, ArH), 7.28 (d, 1H, *J* = 7.6 Hz, ArH), 7.35 (d, 1H, *J* = 7.6 Hz, ArH), 8.44 (s, 1H, C2-H) ppm; ^13^C NMR (100 MHz, DMSO-d_6_): δ 16.3, 26.9, 27.3, 28.5, 29.6, 31.9, 119.5, 122.9, 126.3, 127.6, 130.4, 131.6, 132.7, 140.4, 150.9, 152.0, 162.7, 166.7 ppm; EIMS [*m/z*, %]: 311 [M + 1⌉·^+^, 38.80], 310 [M·^+^, 81.28], 56 [100.00]; Anal. Calcd for C_18_H_18_N_2_OS (310.41): C, 69.65; H, 5.84; N, 9.02. Found: C, 69.88; H, 6.07; N, 9.31.

***4-(p-Tolyloxy)-6,7,8,9-tetrahydro-5H-cyclohepta[4,5]thieno[2,3-d]pyrimidine (5i).*** Yield: 22%; m.p.>300 °C; IR (KBr) *v*_max_: 3032 (C–H aromatic), 2920, 2850 (C–H aliphatic) cm^−1^; ^1^H NMR (400 MHz, DMSO-d6): *δ* 1.70–1.72 (m, 4H, 2CH_2_), 1.87–1.90 (m, 2H, CH_2_), 2.35 (s, 3H, CH_3_), 2.97–2.99 (m, 2H, CH_2_), 3.27–3.30 (m, 2H, CH_2_), 7.15 (d, 2H, *J* = 8.4 Hz, ArH), 7.27 (d, 2H, *J* = 8.4 Hz, ArH), 8.45 (s, 1H, C2–H) ppm; ^13^C NMR (100 MHz, DMSO-d_6_): δ 20.9, 26.9, 27.3, 28.5, 29.6, 31.9, 119.8, 122.2, 130.5, 132.6, 135.2, 140.3, 148.5, 150.3, 152.0, 163.3 ppm; EIMS [*m/z*, %]: 311 [M + 1⌉·^+^, 51.51], 310 [M·^+^, 15.82]; Anal. Calcd for C_18_H_18_N_2_OS (310.41): C, 69.65; H, 5.84; N, 9.02. Found: C, 69.87; H, 5.98; N, 9.31.

***4-(4-Methoxyphenoxy)-6,7,8,9-tetrahydro-5H-cyclohepta[4,5]thieno[2,3-d]pyrimidine (5j)***. Yield: 26%; m.p.>300 °C; IR (KBr) *v*_max_: 3055, 3032 (C–H aromatic), 2920, 2850 (C–H aliphatic) cm^−1^; ^1^H NMR (400 MHz, DMSO-d_6_): *δ* 1.69–1.71 (m, 4H, 2CH_2_), 1.88–1.89 (m, 2H, CH_2_), 2.96–2.98 (m, 2H, CH_2_), 3.26–3.29 (m, 2H, CH_2_), 3.79 (s, 3H, OCH_3_), 7.00 (d, 2H, *J* = 9.2, ArH), 7.19 (d, 2H, *J* = 9.2, ArH), 8.45 (s, 1H, C2-H) ppm; ^13^C NMR (100 MHz, DMSO-d_6_): δ 26.9, 27.3, 28.5, 29.6, 31.8, 55.9, 115.1, 119.7, 123.4, 132.7, 140.2, 145.7, 152.0, 157.2, 163.5, 166.6 ppm; EIMS [*m/z*, %]: 327 [M + 1⌉·^+^, 25.15], 326 [M·^+^, 100.00]; Anal. Calcd for C_18_H_18_N_2_O_2_S (326.41): C, 66.23; H, 5.56; N, 8.58. Found: C, 66.44; H, 5.72; N, 8.80.

***4-(4-Nitrophenoxy)-6,7,8,9-tetrahydro-5H-cyclohepta[4,5]thieno[2,3-d]pyrimidine (5k).*** Yield: 78%; m.p. 166–169 °C; IR (KBr) *v*_max_: 3101, 3059 (C–H aromatic), 2920, 2843 (C–H aliphatic) cm^−1^; ^1^H NMR (400 MHz, DMSO-d_6_): *δ* 1.70–1.72 (m, 4H, CH_2_), 1.88–1.89 (m, 2H, CH_2_), 2.98–3.00 (m, 2H, CH_2_), 3.24–3.27 (m, 2H, CH_2_), 7.61 (d, 2H, *J* = 9.2 Hz, ArH), 8.35 (d, 2H, *J* = 9.2 Hz, ArH), 8.52 (s, 1H, C2-H) ppm; ^13^C NMR (100 MHz, DMSO-d_6_): δ 26.9, 27.3, 28.5, 29.6, 31.8, 120.0, 123.7, 125.9, 132.4, 141.1, 145.2, 151.8, 157.7, 162.2, 167.3 ppm; EIMS [*m/z*, %]: 342 [M + 1⌉·^+^, 29.82], 341 [M·^+^, 100.00]; Anal. Calcd for C_17_H_15_N_3_O_3_S (341.38): C, 59.81; H, 4.43; N, 12.31. Found: C, 59.72; H, 4.61; N, 12.49.

## Biological assay

### Measurement of anticancer activity

Human breast cancer cell line (MCF-7) and the human mammary epithelial cell line (MCF-10A) were obtained from American Type Culture Collection, they were cultured using Dulbecco’s Modified Eagle’s medium (DMEM) (Invitrogen/Life Technologies, Carlsbad, CA) supplemented with 10% fetal bovine serum (FBS) (Hyclone, USA), 10 µg/mL of insulin (Sigma-Aldrich, St. Louis, MO), and 1% penicillin–streptomycin. All of the other chemicals and reagents were purchased from Sigma-Aldrich, or Invitrogen.

### Cell culture protocol

The culture medium was removed to a centrifuge tube. The cell layer was rinsed with 0.25% (w/v) trypsin 0.53 mM EDTA solution by which all traces of serum containing trypsin inhibitor were removed. Then, 2.0–3.0 mL of trypsin EDTA solution was added to a flask and cells were monitored under an inverted microscope until cell layer was dispersed (usually within 5–15 min). Complete growth medium (6.0–8.0 mL) was added. The cell suspension was then centrifuged for 5–10 min. The cell pellet was resuspended in a fresh growth medium and suitable aliquots of the cell suspension were added to new culture vessels. Cultures were incubated at 37 °C for 24 h.

### MTT assay protocol

Anticancer activity of the newly synthesized compounds was evaluated *in vitro* against MCF-7 according to the 3-(4,5-dimethylthiazol-2-yl)-2,5-diphenyltetrazolium bromide (MTT) method^34^. Doxorubicin (Adriamycin^®^) and erlotinib were used as reference standards. Cells were plated (cells density 1.2–1.8 × 10,000 cells/well) in a volume of 100 µL complete growth medium and 100 µL of the tested compound per well in a 96-well plate for 24 h before the MTT assay. Treatment of the cells with different concentrations (0.39, 1.56, 6.25, 25 and 100 µM) of the test compounds, doxorubicin and erlotinib followed by incubation for 48 h at 37 °C. Then cultures were removed from incubator into laminar flow hood or other sterile work area. Each vial of 3-(4,5-dimethylthiazol-2-yl)-2,5-diphenyltetrazolium bromide (MTT) [M-5655] was reconstituted with 3 mL of medium or balanced salt solution without phenol red and serum. Consequently, reconstituted MTT was added in an amount equal to 10% of the culture medium volume. Cultures were incubated for 2–4 h based on cell type and maximum cell density. Cultures were removed from incubator and the afforded formazan crystals were dissolved *via* addition of MTT solubilization solution [M-8910]. Color intensity was measured spectrophotometrically by ROBONIK P2000 spectrophotometer at a wavelength of 570 nm. The percentage of surviving cells was plotted against drug concentration to get the survival curve of MCF-7 after each compound. The IC_50_ value [the concentration required for 50% inhibition of cell viability] for each test compound and reference drug compound doxorubicin and erlotinib was calculated in µM.

### *In vitro* measurement of inhibitory activity of compound 5a, 5b, 5d–g and 5k against EGFR

Compounds **5a**, **5b**, **5d**, **5e**, **5f**, **5g** and **5k** were further submitted for evaluation against EGFR (Cat. # 40321) using EGFR assay kit: cloud clone SEA757Hu 96 tests according to manufacturer’s instructions. The assay kit exits as 96 well form with purified recombinant EGFR enzyme, EGFR substrate, ATP and kinase assay buffer for 100 enzyme reactions. All samples and controls were tested in triplicate. 5× Kinase assay buffer, ATP and 50× PTK substrate were thawed. The master mixture was constructed (25 µL per well), consequently 25 µL was added to every well. About 5 µL inhibitor solution was added to each well labeled as “Test Inhibitor” while for the “Positive control” and “Blank”, 5 µL of the buffer solution without inhibitor (Inhibitor buffer) was added.

About 3 mL of 1× kinase assay buffer was developed through mixing 600 µL of 5× kinase assay buffer with 2400 µL water. About 20 µL of 1× kinase assay buffer was added to the wells designated as “Blank”. EGFR enzyme was thawed on ice. Consequent to the first thaw, the tube containing enzyme was shortly spun so as to recover full content of the tube. The amount of EGFR required for the assay was calculated then the enzyme was diluted to 1 ng/µL with 1× kinase assay buffer. Reaction was initiated *via* addition of 20 µL of diluted EGFR enzyme to the wells designated “Positive Control” and “Test Inhibitor Control” then incubation at 30 °C for 40 min. Kinase-Glo Max reagent was thawed. After the 40 min reaction, 50 µL of Kinase-Glo Max reagent was added to each well. Plates were covered with aluminum foil and incubated at room temperature for 15 min. Finally, luminescence was measured using the microplate Robonik P2000 ELISA Reader (India). Erlotinib, lapatinib and gefitinib were selected as reference drugs due to their potent inhibitory activity of EGFR.

### *In vitro* measurement of inhibitory activity of compounds 5a, 5b and 5d–f against VEGFR-2

Compounds **5a**, **5b** and **5d–f** was submitted for evaluation against VEGFR-2 [#7788] using recombinant human VEGFR-2/KDR ELISA kit according to manufacturer’s instructions. About 10 µL of 10 mM ATP was added to 1.25 mL 6 µM substrate peptide followed by dilution of the mixture with distilled water to 2.50 mL to make 2× ATP/substrate cocktail ([ATP] = 40 µM, [substrate] = 3 µm). The enzyme was immediately transferred from –80 °C to ice and was allowed to thaw on ice. Microcentrifuge was performed briefly at 4 °C and returned immediately to ice. About 10 µL of dithiothreitol (DTT) (1.25 M) was added to 2.5 mL of 4× HTScan^®^ Tyrosine Kinase Buffer [240 mM 4-(2-hydroxyethyl)-1-piperazineethanesulfonic acid (HEPES) pH 7.5, 20 mM MgCI_2_, 20 mM MnCI_2_, 12 µM Na_3_VO_4_] to make DTT/Kinase buffer. About 0.6 mL of DTT/Kinase buffer was transferred to each enzyme tube to make 4× reaction mixture. Exactly 12.50 µL of the 4× reaction mixture was incubated with 12.50 µL/well of prediluted tested compounds **5a**, **5b** and **5d–f** (around 0.01, 0.1, 1 and 10 µM) for 5 min at room temperature. About 25 µL of 2× ATP/substrate cocktail was added to 25 µL/well pre-incubated reaction mixture/compound. Reaction plate was incubated at room temperature for 30 min. Then, 50 µL/well stop buffer (50 mM EDTA, pH 8) was added to stop the reaction. About 25 µL of each reaction and 75 µL distilled water/well was transferred to a 96-well streptavidin coated plate and incubate at room temperature for 60 min and subsequently washed three times with 200 µL/well PBS (phosphate-buffered saline)/T. Primary antibody, Phospho-Tyrosine mAb (P-Tyr-100) were diluted 1:1000 in PBS/T with 1% BSA (bovine serum albumin). About 100 µL/well primary antibody was added and incubated at room temperature for 60 min followed by washing three times with 200 µL/well PBS/T.

Appropriate dilution of HRP labeled secondary antibody in PBS/T was prepared with 1% BSA (1:500 dilution for anti-mouse IgG or 1:1000 for anti-rabbit IgG). About 100 µL/well secondary antibody solution was added and incubated at room temperature for 30 min then washed five times with 200 µL/well PBS/T followed by addition of 100 µL/well 3,3′,5,5′-tetramethylbenzidine (TMB) substrate and incubation at room temperature for 15 min and finally addition of 100 µL/well of stop solution and mix well. The absorbance was read at 450 nm with a microtiter plate reader. The values of % activity versus a series of compound concentrations (0.01 µM–0.1 µM–1 µM–10 µM) was then plotted using non-linear regression analysis of sigmoidal dose-response curve. The IC_50_ values for compounds **5a**, **5b** and **5d–f** against VEGFR-2 were determined by the concentration causing a half-maximal percent activity and the data were compared with vandetanib as standard VEGFR-2 inhibitor.

### Molecular docking of the compound 5b and 5f

The molecular docking of the compounds **5b** and **5f** were performed using Molecular Operating Environment (MOE, 10.2008; Montreal, Quebec, Canada) software. Compounds were minimized with MOE till an RMSD gradient of 0.05 kcal mol^−1^ Å^−1^ using MMFF94× force field and the partial charges were automatically calculated. The X-ray crystallographic structures of EGFR co-crystallized with erlotinib (PDB ID: 1m17) and VEGFR-2 co-crystallized with tivozanib as inhibitor (PDB ID: 4ASE) were downloaded from the protein data bank. Enzyme structure was inspected at first for missing bonds, atoms and contacts. The receptor was prepared for docking study using Protonate 3D protocol in MOE with default options and water for crystallization was kept as it’s important for binding with the active site. The cocrystallized ligand was used to determine the active site for docking. Triangle matcher placement method and London dG scoring function were used for docking. Docking setup was first validated by re-docking of the cocrystallized ligand (erlotinib and tivozanib) in the pocket of the active site of both receptors with energy score (S)= −10.97 and −17.04 kcal/mol, respectively. The validated setup was subsequently utilized in order to predict the ligands receptor interactions at the active site for compounds **5b** and **5f**. Poses displaying the best superimposition mode on the ligand and binding energy score for each compound were analyzed to identify potential interactions with amino acids in the active site of each enzyme.

### Cell cycle analysis of compound 5f

Cell cycle were carried out using propidium iodide flow cytometry kit (Abcam, ab139418) according to the manufacturer’s instructions. The MCF-7 cells were treated with 0.66 µM of compound **5f** for 24 h. Then, the cells were washed twice with ice-cold PBS, centrifuged and fixed using ice-cold 66% (v/v) ethanol, washed with PBS, re-suspended with 0.1 mg/mL RNase and stained with 200 µL propidium iodide (PI). Finally, cells were analyzed by flow cytometry. The cell cycle distributions were calculated using Cell-Quest software (Becton Dickinson). An interference with the normal cell cycle distribution resulted upon exposure of MCF-7 cells to this compound as indicated.

### Measurement of apoptosis using Annexin-V-FITC apoptosis detection kit

Apoptosis was evaluated utilizing Annexin V-FITC/PI apoptosis detection kit (Biovision, Mountain View, CA #K101-25) according to the manufacturer’s instructions after staining the cells with Annexin V fluorescein isothiocyanate (FITC) and counterstaining with propidium iodide (PI).

About 1–5 × 10^5 ^cells were exposed to compound **5f** at its IC_50_ concentration (0.66 µM) for 24 h. The cells then were centrifuged and in case of adherent cells, cells were gently trypsinized and washed once with serum-containing media followed by resuspension in 500 µL of binding buffer. About 5 µL of Annexin V-FITC and 5 µL of (PI 50 mg/mL) were added and incubated at room temperature for 5 min in the dark. Analyses were carried out using flow cytometer (Ex = 488 nm; Em = 530 nm) using FITC signal detector (usually FL1) and PI staining by the phycoerythrin emission signal detector (usually FL2).

## Results and discussion

### Chemistry

**Figure 3.**
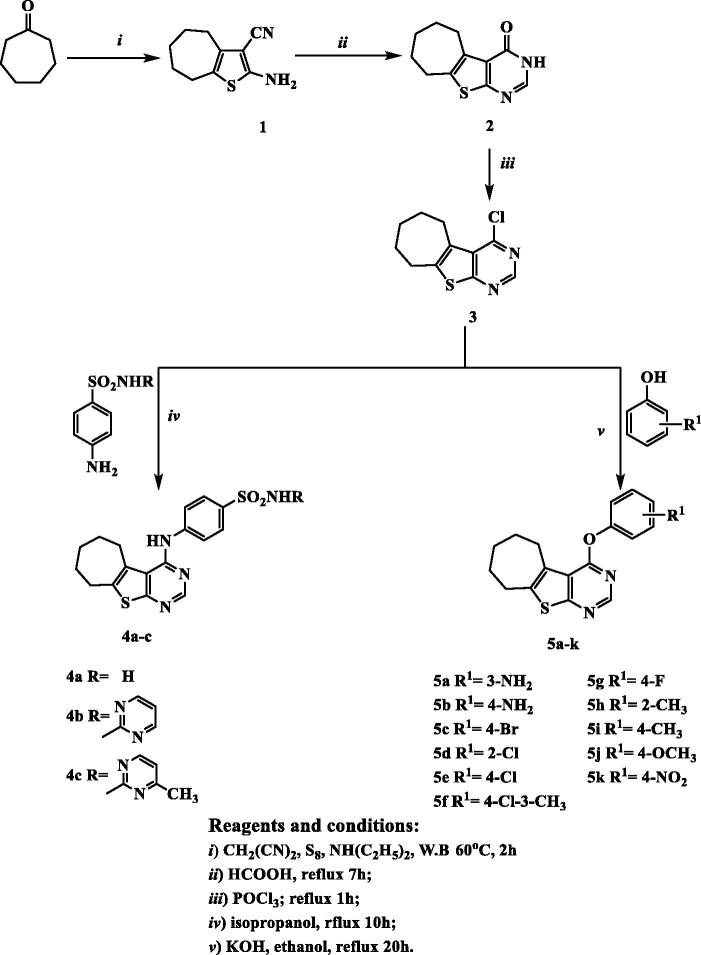
The synthetic pathway and reagents for the preparation of the target compounds **4a–c** and **5a–k**.

The target compounds were synthesized as outlined in Figure 3. Our primary starting material 2-amino-5,6,7,8-tetrahydro-4*H*-cyclohepta[*b*]thiophene-3-carbonitrile (**1**) was synthesized utilizing one step Gewald reaction^35–37^ through the reaction of cycloheptanone with malononitrile and sulfur in presence of catalytic amount of diethylamine as reported by Arya^33^. Reacting compound **1** with formic acid gave 6,7,8,9-tetrahydro-3*H*-cyclohepta[4,5]thieno[2,3-*d*]pyrimidin-4(5*H*)-one (**2**) which was subsequently underwent chlorination upon heating under reflux with phosphorus oxychloride to afford 4-chloro-6,7,8,9-tetrahydro-5*H*-cyclohepta[4,5]thieno[2,3-*d*]pyrimidine (**3**) as reported^33^. Reacting compound **3** with the appropriate substituted benzenesulphonamide in isopropanol resulted in 4-arylaminothienopyrimidine derivatives **4a–c**. IR spectra showed the appearance of two absorption bands at 3456–3228 cm^−1^ corresponding to NH, NH_2_ groups. Moreover, ^1^H NMR spectrum of compound **4a** elucidated two exchangeable singlet peaks at *δ* 7.22 and 9.05 ppm relative to NH and NH_2_ protons. Additionally, compounds **4b** and **4c** showed two exchangeable singlet peaks at *δ* 6.00, 9.07 and 4.77, 9.11 ppm corresponding to two NH protons. It also displayed the expected signals corresponding to the aromatic protons of **4a–c**, compound **4c** further revealed the appearance of CH_3_ group as singlet signal at *δ* 2.33 ppm. Additionally, ^13^C NMR spectra of compounds **4a–c** was as a further evidence and it revealed an increase in absorbance peaks in the aromatic region corresponding to the aromatic carbons. Furthermore, compound **4c** showed absorbance peak at *δ* 23.7 ppm corresponding to CH_3_. The nucleophilic substitution of 4-chlorothienopyrimidine **3** with different substituted phenols was achieved *via* dissolving various substituted phenols in potassium hydroxide and ethanol in order to form potassium phenoxide that subsequently heated under reflux with 4-chlorothienopyrimidine **3** to afford compounds **5a–k**. Compounds **5a–k** were verified by spectral data and elemental analysis. IR spectra of compound **5a** and **5b** showed two absorption bands at 3452–3325 and 3440–3400 cm^−1^, respectively which corresponding to NH_2_ groups. Furthermore, ^1^H NMR revealed the expected signals of the aromatic protons corresponding to the substituted phenoxy rings which appear at range of *δ* 6.34–8.52 ppm, also compounds **5a** and **5b** confirmed the presence of NH_2_ group as singlet exchangeable signal at *δ* 5.28 and 5.12 ppm, respectively. In addition, compound **5f**, **5h** and **5i** showed singlet signal at *δ* 2.34, 2.08 and 2.35 ppm, respectively which is an evidence for the presence of CH_3_ group while ^1^H NMR spectrum of compound **5j** elucidated the O-CH_3_ group as singlet signal at *δ* 3.79 ppm. On the other hand, ^13^C NMR revealed the peaks corresponding to the carbon atoms in compounds **5a–k**, additionally, compound **5f**, **5h** and **5i** showed absorbance peak at 20.0, 16.3 and 20.9 ppm, respectively which are corresponding to CH_3_ group while compound **5j** displayed absorbance peak at *δ* 55.9 ppm confirming the presence of the O–CH_3_ group.

## Biological assay

### *In vitro* anticancer activity

**Figure 4.**
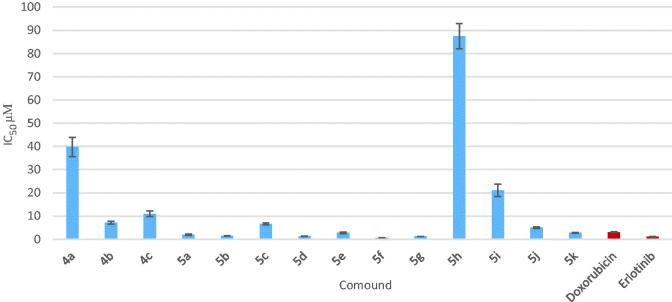
Graphical representation of IC_50_ expressed in µM of test compounds against MCF-7 cell line compared to doxorubicin and erlotinib.

The newly synthesized compounds were evaluated for their anticancer activity against MCF-7 cell line compared to doxorubicin and erlotinib as reference anticancer drugs using MTT assay. The results expressed as half maximal inhibitory concentration (IC_50_) values, were summarized in Table 1 and graphically illustrated in Figure 4. According to the *in vitro* results, it was revealed that all of the test compounds except **5h** exhibited moderate to significant anticancer activity against MCF-7 cell line. Seven compounds **5a, 5b, 5d–g** and **5k** showed 4.64–1.11-folds more potent anticancer activity than doxorubicin, six of them **5a**, **5b**, **5d**, **5e**, **5g** and **5k** were comparable to erlotinib while compound **5f** was more potent than erlotinib. Four compounds **4b**, **4c**, **5c** and **5j** displayed anticancer activity comparable to both doxorubicin and erlotinib with IC_50_ values 7.12, 10.98, 6.65 and 5.01 µM, respectively. Compounds **4a** and **5i** showed moderate anticancer activity. From the activity results, the relation between the test compounds and the activity can be concluded as follows:

**Table 1.** Results of *in vitro* anticancer activity of the newly synthesized compounds **4a–c** and **5a–k** against MCF-7 cell line.
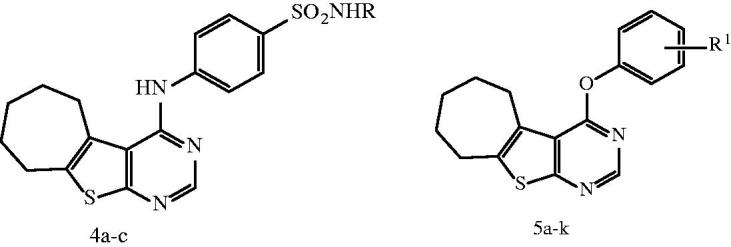

Compound No.	R	R^1^	IC_50_^a^, µM*±SD	IC_50_^b^, µM*±SD	Selectivity index
**4a**	H	–	39.75 ± 4.11	–	–
**4b**		–	7.12 ± 0.55	–	–
**4c**		–	10.98 ± 1.24	–	–
**5a**	–	3-NH_2_	1.98 ± 0.21	–	–
**5b**	–	4-NH_2_	1.47 ± 0.11	72.74 ± 3.98	49.5
**5c**	–	4-Br	6.65 ± 0.41	–	–
**5d**	–	2-Cl	1.34 ± 0.07	–	–
**5e**	–	4-Cl	2.76 ± 0.33	–	–
**5f**	–	4-Cl-3-CH_3_	0.66 ± 0.05	153.16 ± 7.57	232.1
**5g**	–	4-F	1.23 ± 0.04	–	–
**5h**	–	2-CH_3_	87.43 ± 5.4	–	–
**5i**	–	4-CH3	21.07 ± 2.7	–	–
**5j**	–	4-OCH_3_	5.01 ± 0.31	–	–
**5k**	–	4-NO_2_	2.76 ± 0.19	–	–
Doxorubicin	–	–	3.06 ± 0.19	27.58 ± 1.55	–
Erlotinib	–	–	1.14 ± 0.05	69.41 ± 4.24	–

a IC_50_ on MCF-7 cell line.

b IC_50_ on normal mammary epithelial cell line (MCF-10A).

* The results given are means of three experiments.

1. The introduction of different substituents at C-4 position has pronounced influence on the antitumor activity.
  - Presence of benzenesulphonamide moieties compounds **4a–c** exhibited moderate activity, Furthermore, compounds **4b** and **4c** bearing pyrimidine ring in benzenesulphonamide moiety displayed more potent anticancer activity than **4a**. On the other hand, in compound **4c**, introduction of electron donating group (–CH_3_) at position-4 of the pyrimidine ring showed slight decrease in the anticancer activity compared with **4b.**
  - Replacement of the benzenesulphonamide substituents with phenoxy moieties **5a–k** showed marked increase in the anticancer activity. Additional analysis of compounds **5a–k** elucidated that the presence of H-bond donor group such as amino group in compounds **5a** and **5b** interestingly enhanced the anticancer activity. Moreover, introduction of H-bond acceptor groups such as methoxy or nitro groups (compounds **5j** and **5k**, respectively) resulted in potent anticancer activity.
    The potency was also favored by substitution with halogens, compounds **5c–g** (with IC_50_ = 0.66–6.65 µM). Moreover, compound **5f** that showed presence of methyl group at position-3 in addition to 4-chloro atoms revealed the most potent anticancer activity with IC_50_ in micromolar range (IC_50_ = 0.66 µM).
2. Furthermore, our study also demonstrated that 4-substituted aryloxy derivatives were more potent anticancer activity than 2- and 3-substituted ones, such as compound compare **5a** with **5b**, compound **5f** with **5d** and compound **5i** with **5h** position^14^.
3. Finally, compounds **5b** and **5f** (the most potent compounds in the present work) were tested for their possible anticancer activity on the human mammary epithelial cell line (MCF-10A). The results revealed that the compounds showed IC_50_ of 72.74 and 153.16 µM, respectively. They exhibited approximately 49.5–232 times more selectivity against the tumor cell line.

### Measurement of *in vitro* EGFR and VEGFR-2 inhibition

**Figure 5.**
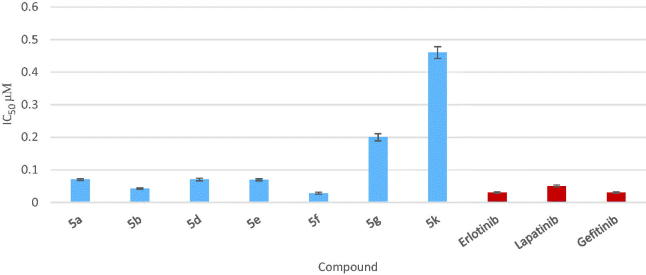
Graphical representation for IC_50_ of EGFR assay in µM of compounds **5a**, **5b**, **5d–g** and **5k**.

The target compounds **5a**, **5b, 5d–g** and **5k** that showed potent anticancer activity against MCF-7 breast cancer cell line were tested for their inhibitory activity against EGFR. The IC_50_ value of the tested compounds were evaluated compared to reference EGFR inhibitors (erlotinib, gefitinib and lapatinib). Compounds **5a**, **5d** and **5e** exhibited comparable inhibitory activity on EGFR to that of the reference EGFR inhibitor lapatinib but compounds **5b** and **5f** displayed excellent EGFR inhibitory activity, at the sub-micromolar level with IC_50_ value 0.042 and 0.028 µM, respectively, compound **5b** was 1.24-folds more potent than lapatinib but comparable with both erlotinib and gefitinib, while compound **5f** was 1.83–1.18-folds more potent than the three reference drugs erlotinib, gefitinib and lapatinib (IC_50_ = 0.033, 0.038 and 0.052 µM, respectively) (Table 2 and Figure 5).

**Table 2.** Results of *in vitro* EGFR kinase activity of compounds **5a**, **5b**, **5d–g** and **5k**.

Compound No.	IC_50_, µM* ± SD
**5a**	0.07 ± 0.003
**5b**	0.042 ± 0.002
**5d**	0.07 ± 0.004
**5e**	0.069 ± 0.003
**5f**	0.028 ± 0.003
**5g**	0.20 ± 0.011
**5k**	0.46 ± 0.018
Erlotinib	0.03 ± 0.002
Lapatinib	0.05 ± 0.003
Gefitinib	0.03 ± 0.002

* The results given are means of three experiments.

Examining the structures and EGFR inhibitory activity of the selected compounds demonstrated the following structure–activity relationship:

- Presence of *p*-amino substituent (in compound **5b**) interestingly enhanced the EGFR inhibitory activity while changing the position of amino group from *p*- to *m*-position decreased the enzyme inhibition activity (compare compound **5a** and **5b**).
- Changing *p*-amino substituent by H-acceptor group (*p*-nitro) dramatically decreased the enzyme inhibition activity (compare compound **5b** and **5k**).

Introduction of chloro substituent in the aryloxy ring slightly increased the EGFR inhibitory activity (compound **5d** and **5e**) while replacing *p*-chloro with *p*-fluoro greatly decreased the enzyme inhibition (compare compound **5e** and **5g**). On the other hand, compound **5f** that possessed (3-CH_3_) group in addition to *p*-chloro substituent revealed potent EGFR inhibitory activity.

Furthermore, the compounds **5a**, **5b** and **5d–f** that exhibited potent EGFR inhibitory activity were further submitted for VEGFR-2 biological enzyme assay to confirm the dual inhibitory activity of these compounds.

**Figure 6.**
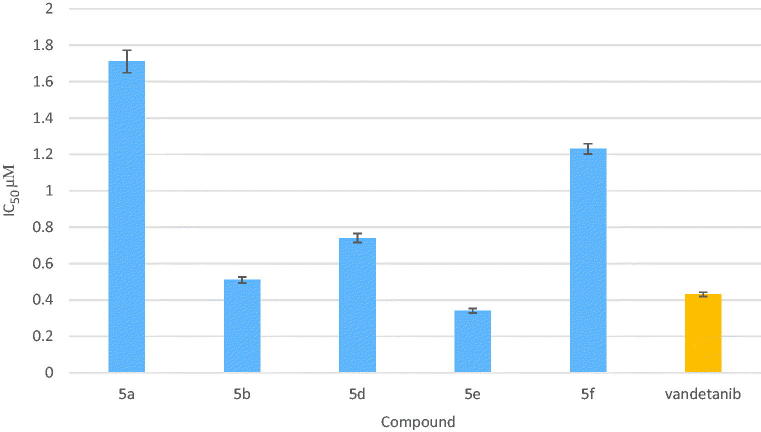
Graphical representation for IC_50_ of VEGFR-2 assay in µM of compounds **5a**, **5b** and **5d–f**.

The IC_50_ value of compounds **5a**, **5b** and **5d–f** were compared to reference VEGFR-2 inhibitor (vandetanib). Compounds **5b**, **5d** and **5f** showed good VEGFR-2 inhibitory activity, at the sub-micromolar level with IC_50_ value 0.51, 0.74 and 1.23 µM, respectively, which were comparable to that of vandetanib (IC_50_ = 0.43 µM), moreover, compound **5e** exhibited the most potent VEGFR-2 inhibitory activity with IC_50_ value 0.34 µM while compound **5a** displayed the least VEGFR-2 inhibitory activity with IC_50_ value 1.71 µM compared to vandetanib (Table 3 and Figure 6).

**Table 3.** Results of *in vitro* VEGFR-2 kinase activity of compounds **5a**, **5b** and **5d–f**.

Compound No.	IC_50_, µM*±SD
**5a**	1.71 ± 0.062
**5b**	0.51 ± 0.016
**5d**	0.74 ± 0.025
**5e**	0.34 ± 0.013
**5f**	1.23 ± 0.028
Vandetanib	0.43 ± 0.012

* The results given are means of three experiments.

Examining the structures of the selected compounds demonstrated the following structure–activity relationship as dual EGFR and VEGFR-2 inhibitors:

- Presence of H-bond donor group (NH_2_) in *p*-position of the aryloxy moiety (compound **5b**) showed potent dual EGFR and VEGFR-2 inhibitory activity with IC_50_ = 0.042 and 0.51 µM, respectively.
- Replacement of *p*-amino group with halogen and methyl group (compound **5f**) showed potent EGFR inhibitory activity but moderate VEGFR-2 inhibitory activity with IC_50_ = 0.028 and 1.23 µM, respectively.

### Molecular docking of compounds 5b and 5f in the active site of EGFR

**Figure 7.**
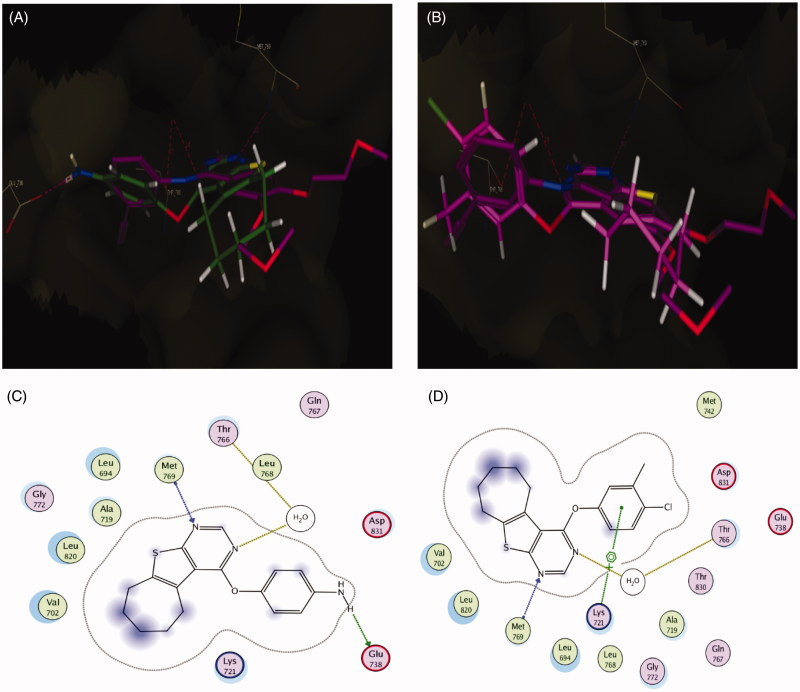
(A) Superposition of **5b** (green) with erlotinib (magenta) inside the EGFR tyrosine kinase ATP-binding site. (B) Superposition of **5f** (pink) with erlotinib (magenta) inside the EGFR tyrosine kinase ATP-binding site. (C) Binding interactions of **5b** (green) with EGFR showing good binding energy score value (score: −10.13). (D) Binding interactions of **5f** (pink) with EGFR showing the best binding energy score value (score: −10.51).

Docking study was carried out for compounds **5b** and **5f** which showed excellent activity in the EGFR enzyme inhibition assay to illustrate the molecular reasons for the observed inhibition activity profile, such as the amino acids in the EGFR active site that supposed to be involved in the expected hydrogen bonding interactions. Based on the obtained results with the semi-rigid docking approach, the novel synthesized 4-aryloxythieno[2,3-*d*]pyrimidine compounds interacted in the EGFR ATP-binding site where the N1 of pyrimidine ring interacted as an H-bond acceptor with the amino acid Met 769 and N3 of pyrimidine ring also as an H-bond acceptor interacted through water molecule with the amino acid Thr 766 in a similar orientation to that of the co-crystallized drug erlotinib in the reference crystallographic structure (PDB ID: 1m17), as shown in Figure 7(A,B). As previously mentioned, the increased potency of compound **5b** of almost 1.24-folds more potent than lapatinib but comparable with both erlotinib and gefitinib against EGFR (IC_50_ = 0.042 µM) could be related to the hydrogen bond donating ability of the amino group, which provides an additional interaction with the side chain of amino acid Glu738 (Figure 7(C)). For compound **5f**, which displayed the most significant inhibitory activity being 1.83–1.18-folds more potent than erlotinib, gefitinib and lapatinib could be attributed to the presence of arene cation interaction between the phenyl ring and the amino acid Lys721 (Figure 7(D). Furthermore, the significant activity of compound **5b** and **5f** against EGFR (IC_50_ = 0.042 and 0.028 µM) was supported by their good docking score (−10.13 and −10.51 kcal/mol, respectively), which was comparable to that of erlotinib (−10.97 kcal/mol), additionally, their excellent superimposition on the ligand (erlotinib) in the active site.

### Molecular docking of compound 5b and 5f in the active site of VEGFR-2

Docking study was performed for compound **5b** and **5f** which displayed good activity in the VEGFR-2 enzyme inhibition assay, which were comparable to that of vandetanib, in order to illustrate the binding mode of these compounds in the active site of the VEGFR-2. Accordingly, we used (PDB ID: 4ASE), which has VEGFR-2 co-crystallized with tivozanib as inhibitor. Also, vandetanib which was chosen as a reference compound depending on similarity in its structure with those of compounds **5b** and **5f** was docked to ensure the validation of VEGFR-2 inhibitory activity results of both **5b** and **5f**.

**Figure 8.**
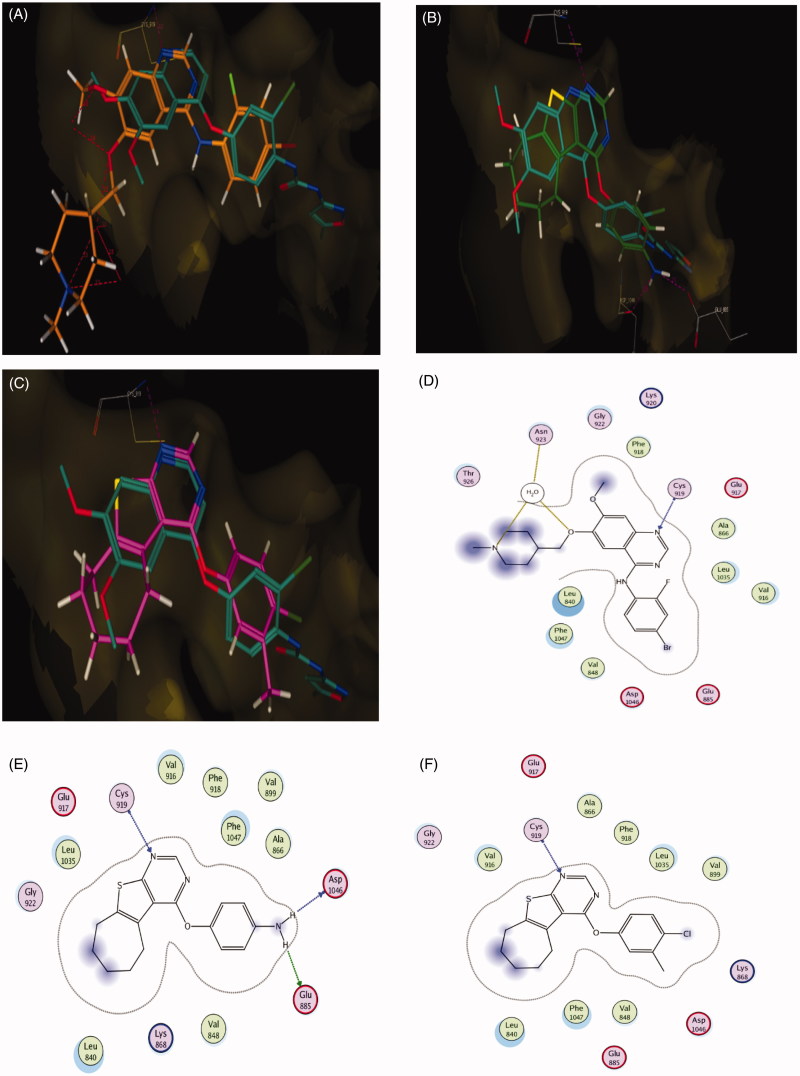
(A) Superposition of vandetanib (orange) with tivozanib (blue) inside the VEGFR-2 tyrosine kinase ATP-binding site. (B) Superposition of **5b** (green) with tivozanib (blue) inside the VEGFR-2 tyrosine kinase ATP-binding site. (C) Superposition of **5f** (pink) with tivozanib (blue) inside the VEGFR-2 tyrosine kinase ATP-binding site. (D) 2D binding interactions of vandetanib (orange) with EGFR showing the best binding energy score value (score: −18.48). (E) 2D binding interactions of **5b** (green) with VEGFR-2 showing good binding energy score value (score: −12.02). (F) 2D binding interactions of **5f** (pink) with EGFR showing the best binding energy score value (score: −12.90).

According to results elucidated in (Figure 8), the reference compound vandetanib and the novel synthesized 4-aryloxythieno[2,3-*d*]pyrimidine compounds **5b** and **5f** interact in VEGFR-2 ATP-binding site through N1 of the pyrimidine ring as H-bond acceptor with the key amino acid Cys919, following an orientation similar to the co-crystallized tivozanib, as shown in Figure 8(A–C). The good activity of compound **5b** and its superior activity when compared to **5f** (IC_50_ = 0.51 µM) probably correlated with the ability of the amino group to form two hydrogen bonds with the side chain of the amino acid Glu885 and the C=O from the Asp1046 backbone (Figure 8(E,F)). These findings support results previously described in the literature^38–40^ which indicate the correlation of hydrogen bond interactions with these amino acids for VEGFR-2 potent inhibition. Additionally, the good activity of compounds **5b** and **5f** against VEGFR-2 (IC_50_ = 0.51 and 1.23 µM, respectively) were validated by their good docking score (−12.02 and −12.90 kcal/mol, respectively).

### Cell cycle analysis and detection of apoptosis

**Figure 9.**
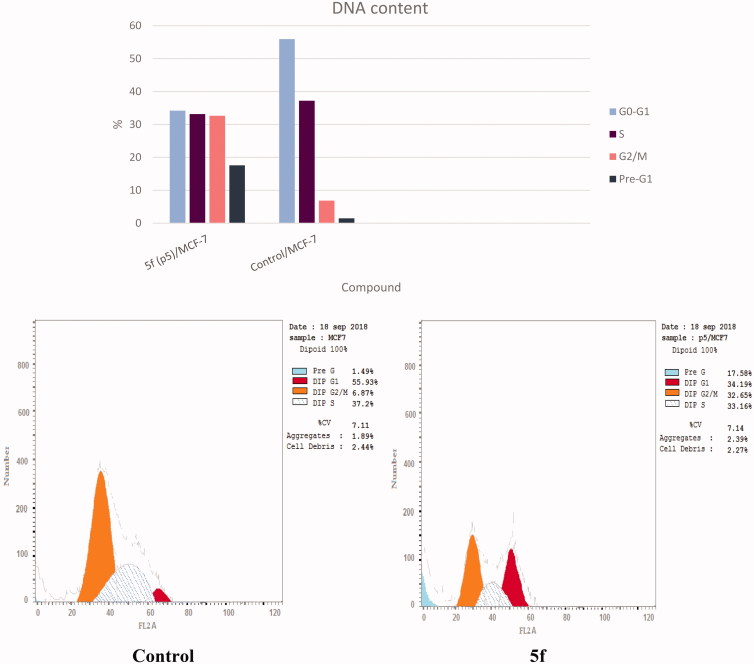
Effect of compound **5f** (0.66 μM) on DNA-ploidy flow cytometric analysis of MCF-7 cells after 24 h.

The most potent compound **5f** was chosen for further investigation of its effects on cell cycle progression and induction of apoptosis in the MCF-7 cell line. Exposure of MCF-7 cells to compound **5f** at its IC_50_ value for 24 h and its effect on the normal cell cycle profile and induction of apoptosis were analyzed. Exposure of MCF-7 cells to compound **5f** caused an interference with the normal distribution of cell cycle of this cell line. Compound **5f** induced a pronounced increase in the percentage of cells at pre-G1 and G2/M phases by 11.80 and 4.75-folds, respectively (Table 4 and Figure 9) compared to control. Accumulation of cells in pre-G1 phase, confirmed by the presence of a sub-G1 peak in the cell cycle profile analysis, which may have resulted from degradation or fragmentation of the genetic material, indicating a possible role of apoptosis in compound **5f**-induced cancer cell death and cytotoxicity. Moreover, G2 arrest may have resulted in the accumulation of the cells in G2/M phase.

**Table 4.** Results of cell cycle inhibition activity of compound **5f**.

Compound No.	%G0–G1	%S	%G2/M	%Pre-G1
**5f**/MCF-7	34.19	33.16	32.65	17.58
Control/MCF-7	55.93	37.2	6.87	1.49

### Apoptosis detection by annexin V-FITC assay

**Figure 10.**
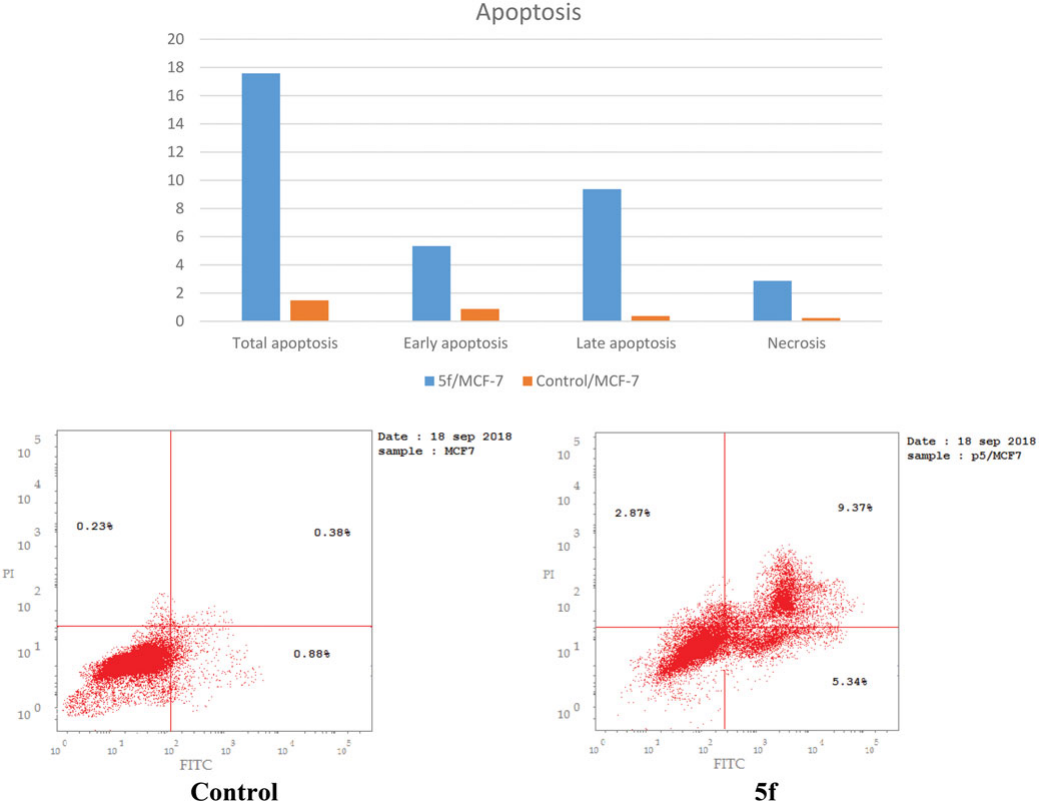
Representative dot plots of MCF-7 cells treated with **5f** (0.66 μM) for 24 h and analyzed by flow cytometry after double staining of the cells with annexin-V FITC and PI.

The ability of compound **5f** to induce apoptosis was investigated through a biparametric cytofluorimetric analysis, using propidium iodide (PI), which stains DNA and is allowed to enter only the dead cells, and fluorescent conjugate of the protein annexin V with fluorescein isothiocyante (FITC), that binds to phosphatidylserine (PS) expressed on the surface of the apoptotic cells and fluoresces green, after interacting with the labeled annexin-V. Dual staining of annexin-V with PI grants differentiation between viable cells, early apoptotic cells, late apoptotic cells and necrotic cells. As shown in Table 5 and Figure 10, after 24 h of treatment with compound **5f** at its IC_50_ concentration (0.66 µM) a decrease in the percentage of the survived cells was observed. Compound **5f** caused a pronounced elevation in the percentage of the total apoptosis 17.58% compared to control which showed 1.49% and the percentage of the necrosis 2.87% which was higher than control that demonstrated only 0.23%. Moreover, a significant increase in the percentage of annexin-V positive cells (6.07-folds more than control) occurred, indicating an early apoptosis (lower right quadrant). Furthermore, the study showed significant increase in the percentage of the late apoptotic and necrotic cells (annexin-V positive, PI positive cells) about 24.66-folds (upper right quadrant) and 12.48-folds (upper left quadrant) more than control.

**Table 5.** Results of apoptotic activity of compound **5f**.

Compound No.	Apoptosis			
	%Total	%Early	%Late	Necrosis
**5f**/MCF-7	17.58	5.34	9.37	2.87
Control/MCF-7	1.49	0.88	0.38	0.23

## Conclusion

A series of novel 4-substituted-6,7,8,9-tetrahydro-5*H*-cyclohepta[4,5]thieno[2,3-*d*]pyrimidine derivatives was synthesized and was evaluated for their anticancer activity against MCF-7 cell line. Seven compounds **5a, 5b, 5d–g** and **5k** showed 4.64–1.11-folds more potent anticancer activity than doxorubicin, six of them **5a**, **5b**, **5d**, **5e**, **5g** and **5k** were comparable to erlotinib while compound **5f** showed the most significant anticancer activity being 4.64 and 1.73-folds more potent than both doxorubicin and erlotinib, respectively. Moreover, Compounds **5b** revealed potent dual EGFR and VEGFR-2 inhibitory activity with IC_50_ = 0.042 and 0.51 µM, respectively. Molecular docking attributed its potent dual activity to the presence of additional hydrogen bonding interaction of the *p*-amino group with Glu738 amino acid of EGFR enzyme and with Asp1046 and Glu88 of VEGFR-2. On the other hand, compound **5f** that displayed potent EGFR inhibitory activity with moderate VEGFR-2 inhibitory activity with IC_50_ = 0.028 and 1.23 µM, respectively. The increased potency of compound **5f** against EGFR compared to its potency against VEGFR-2 enzyme could be related to its ability to form extra arene cation interaction between the phenyl ring and the amino acid Lys721 of EGFR enzyme.

Also, compound **5f** caused a pronounced increase in the percentage of cells at pre-G1 and G2/M phases by 11.80 and 4.75-folds, respectively, compared to control, which confirm a possible role of apoptosis in compound **5f**-induced cancer cell death and cytotoxicity. Compound **5f** displayed potent apoptotic activity.

## Supplementary Material

Supplemental Material
