# Supplementary figures and images for: Novel thienopyrimidine derivatives as dual EGFR and VEGFR-2 inhibitors: design, synthesis, anticancer activity and effect on cell cycle profile
Source: J Enzyme Inhib Med Chem. 2019 Mar 28;34(1):838–52. doi: 10.1080/14756366.2019.1593160 (PMC6442109; doi:10.1080/14756366.2019.1593160)

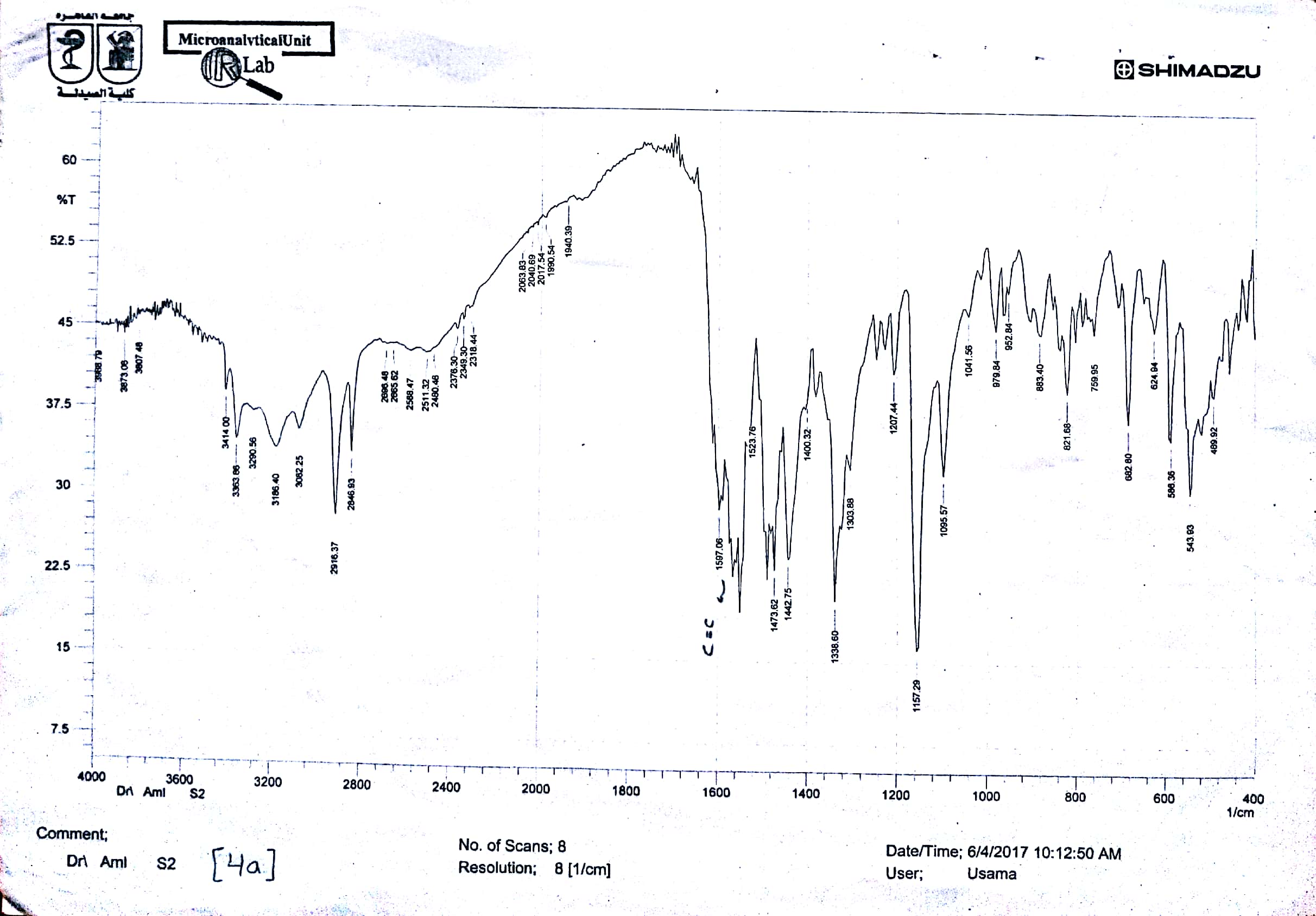

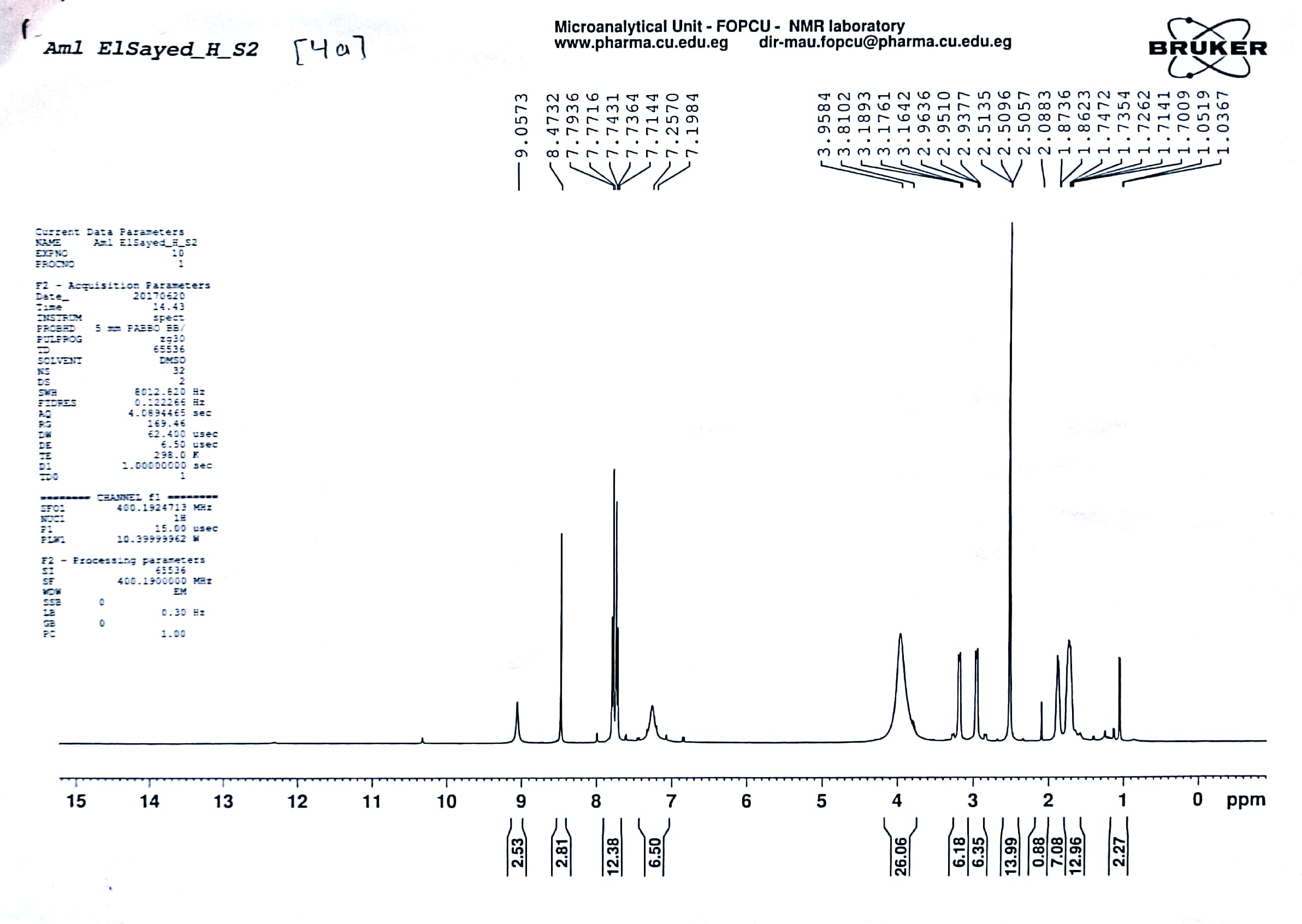

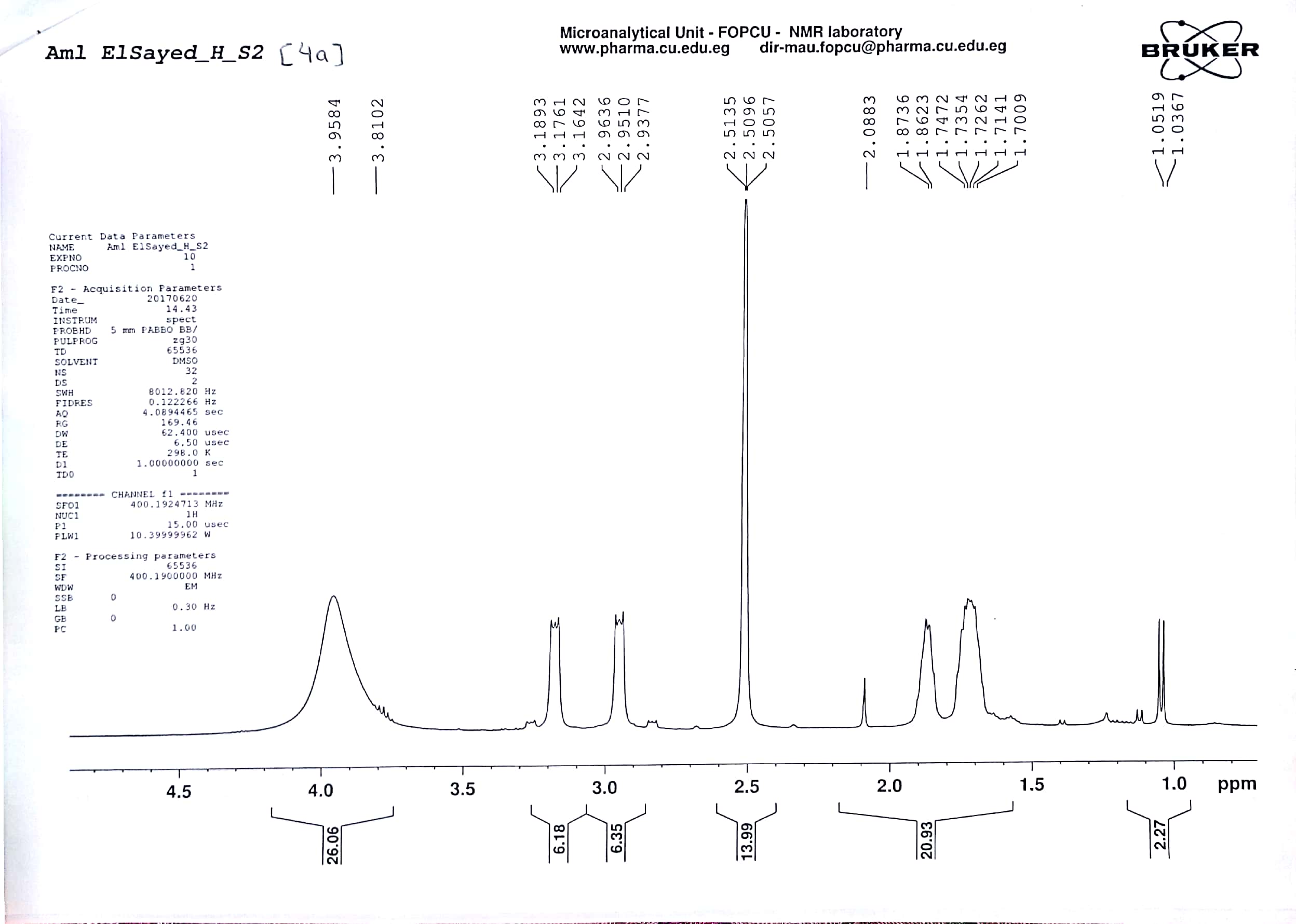

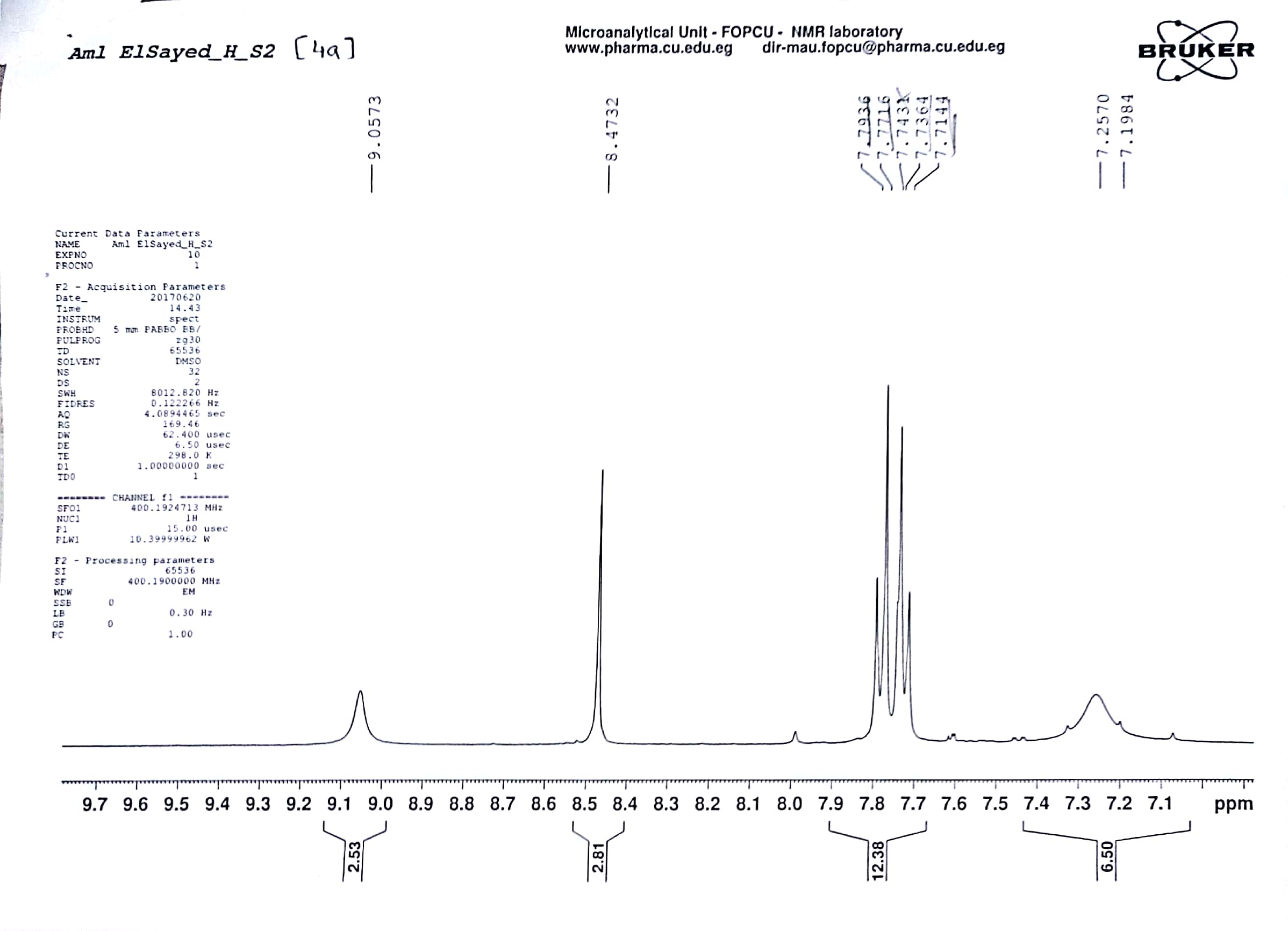

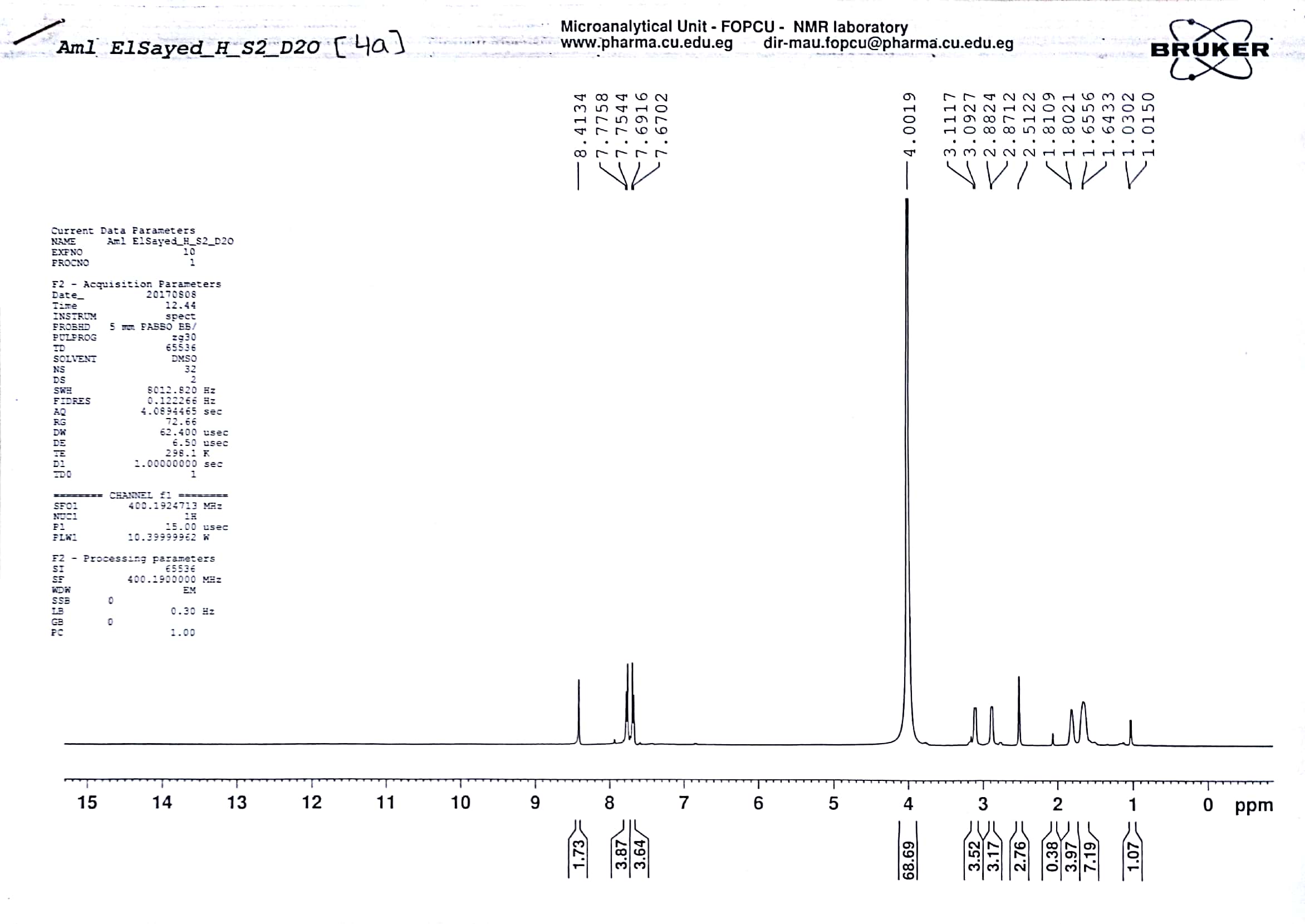

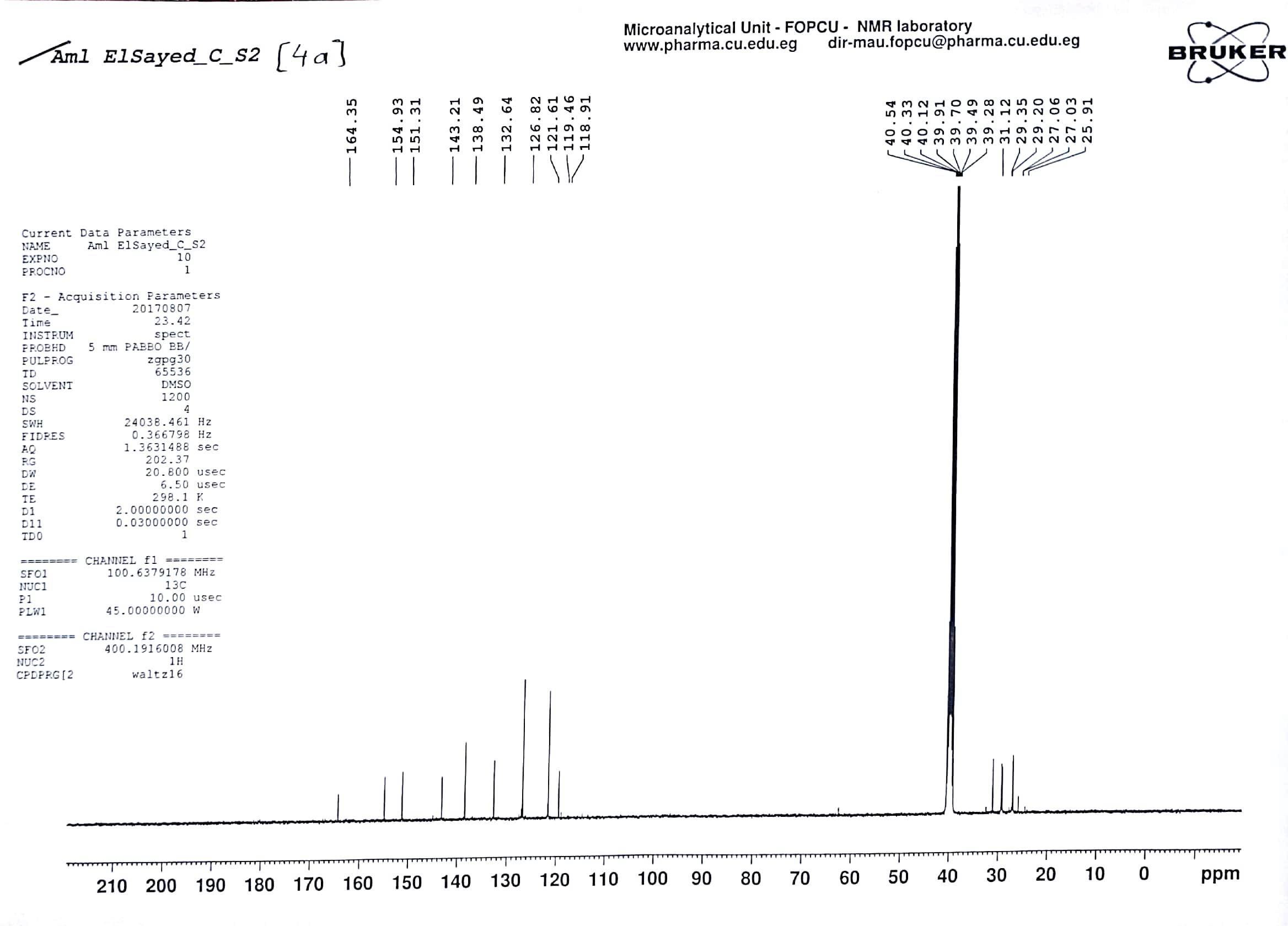

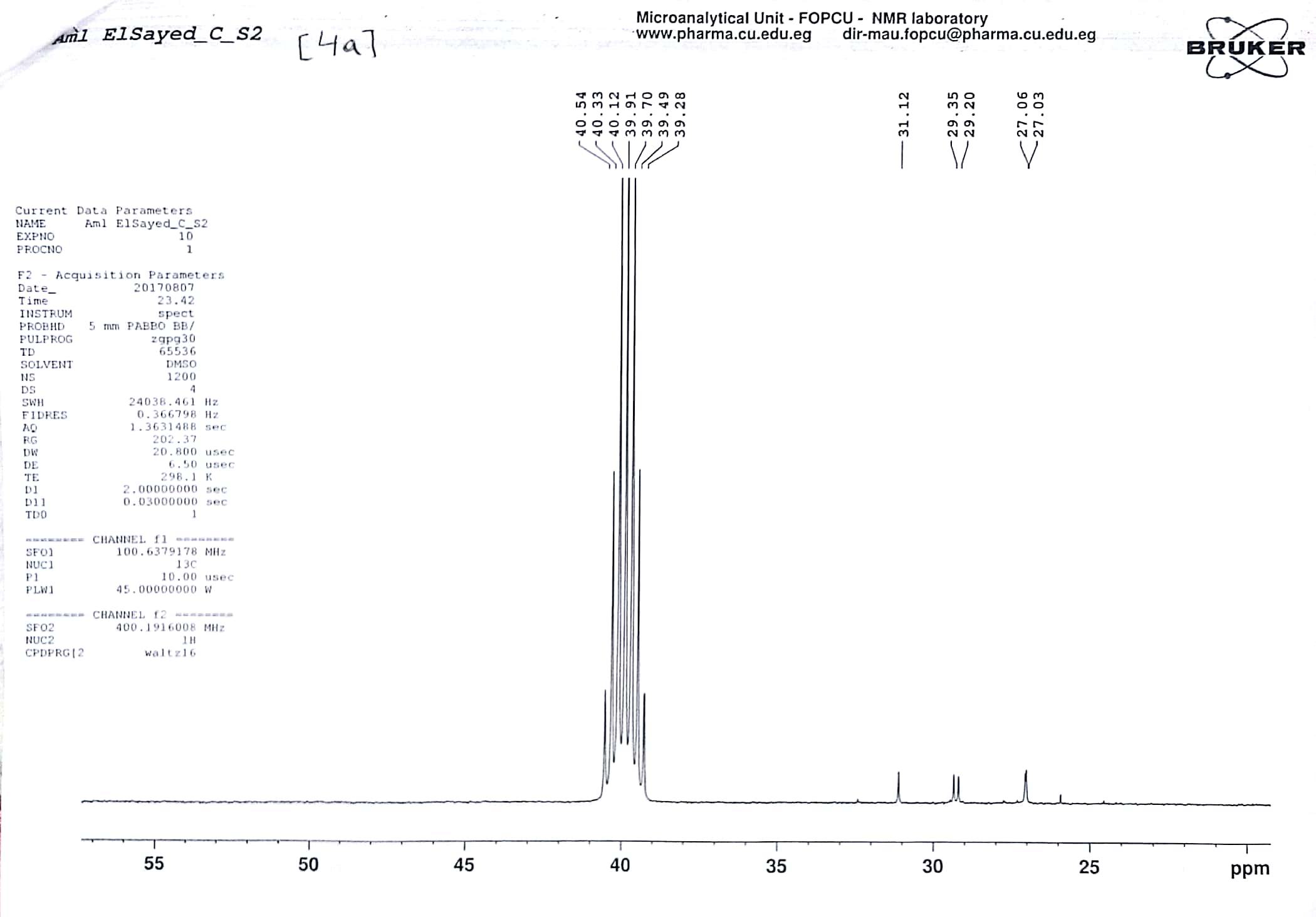

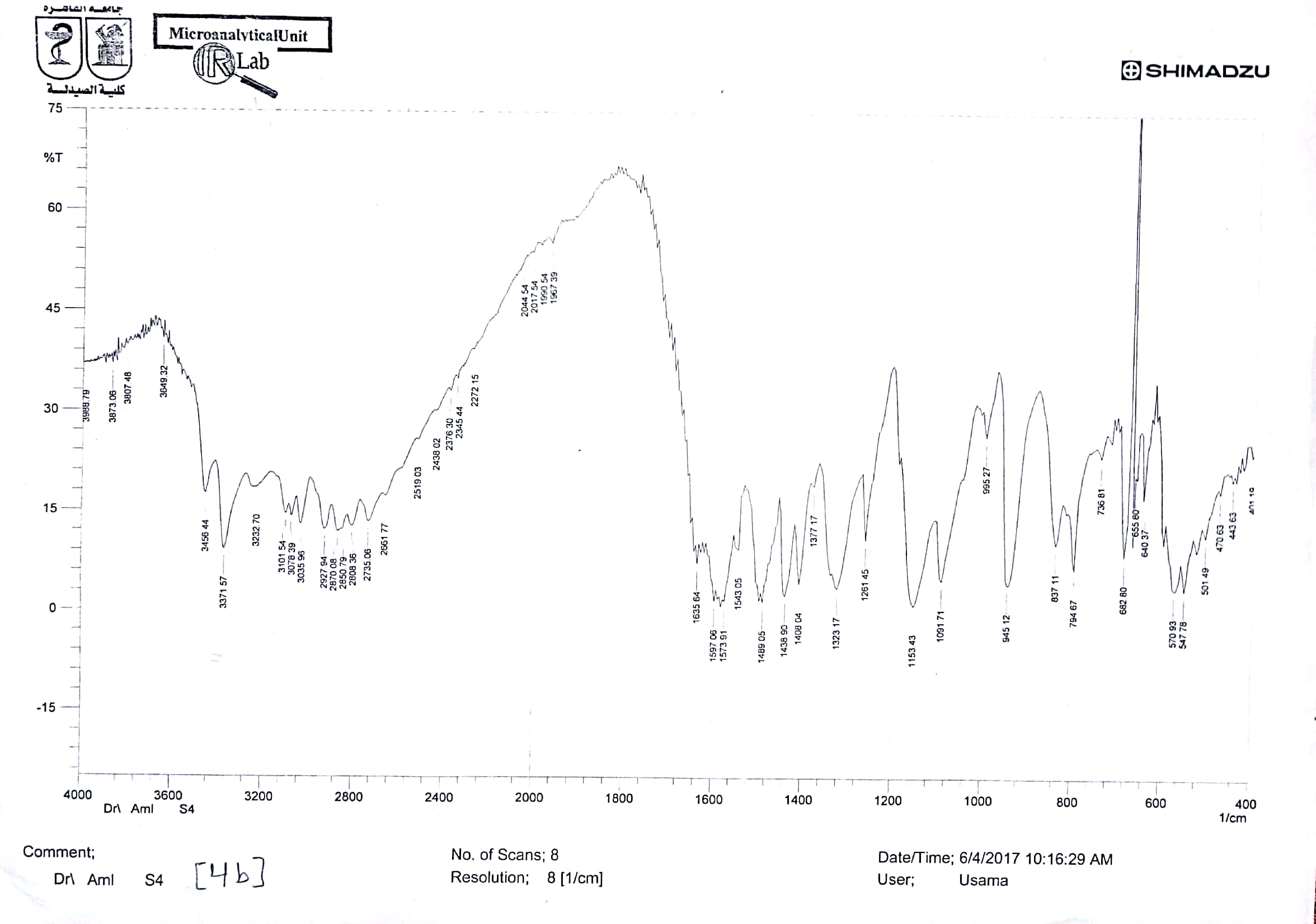

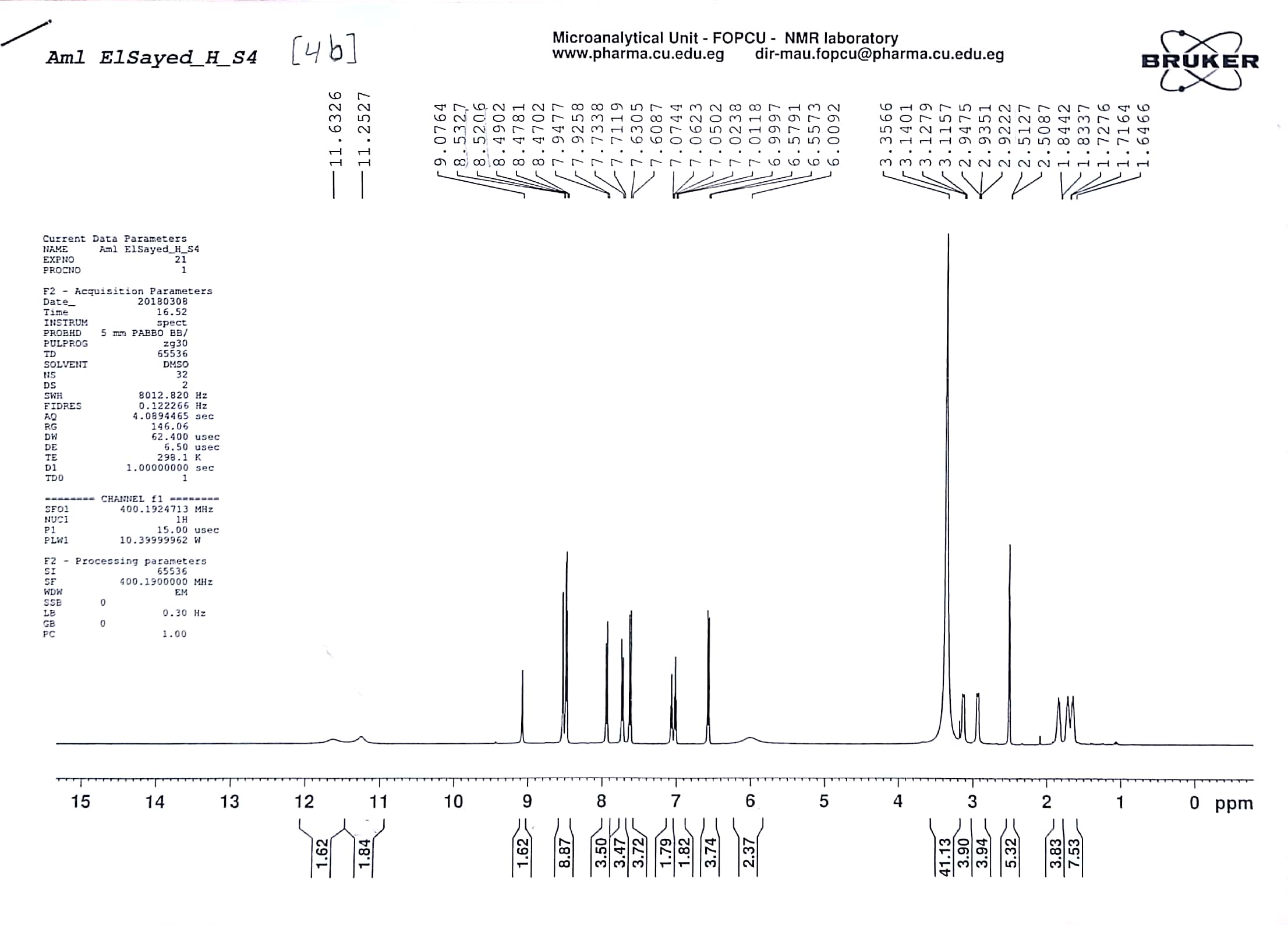

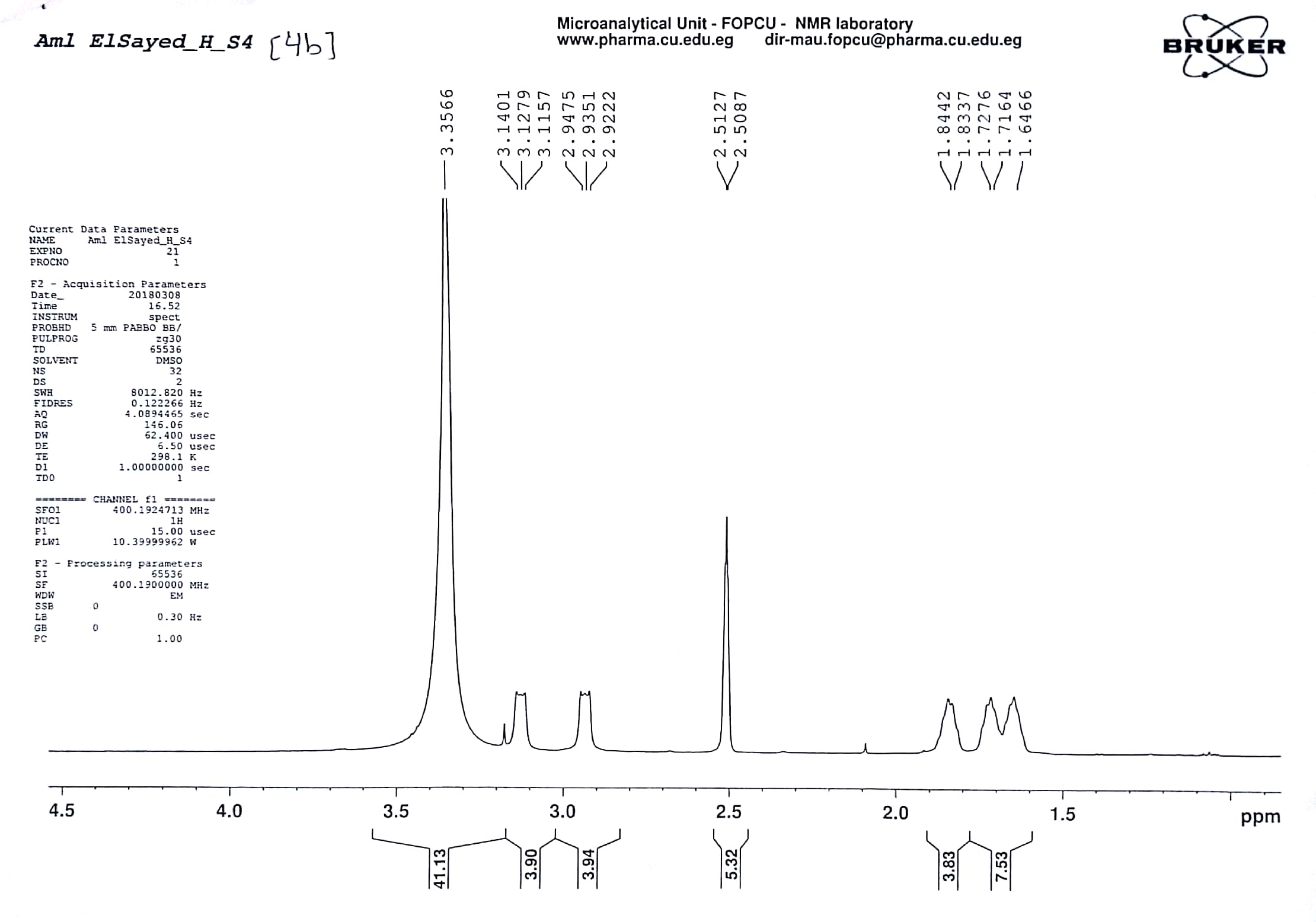

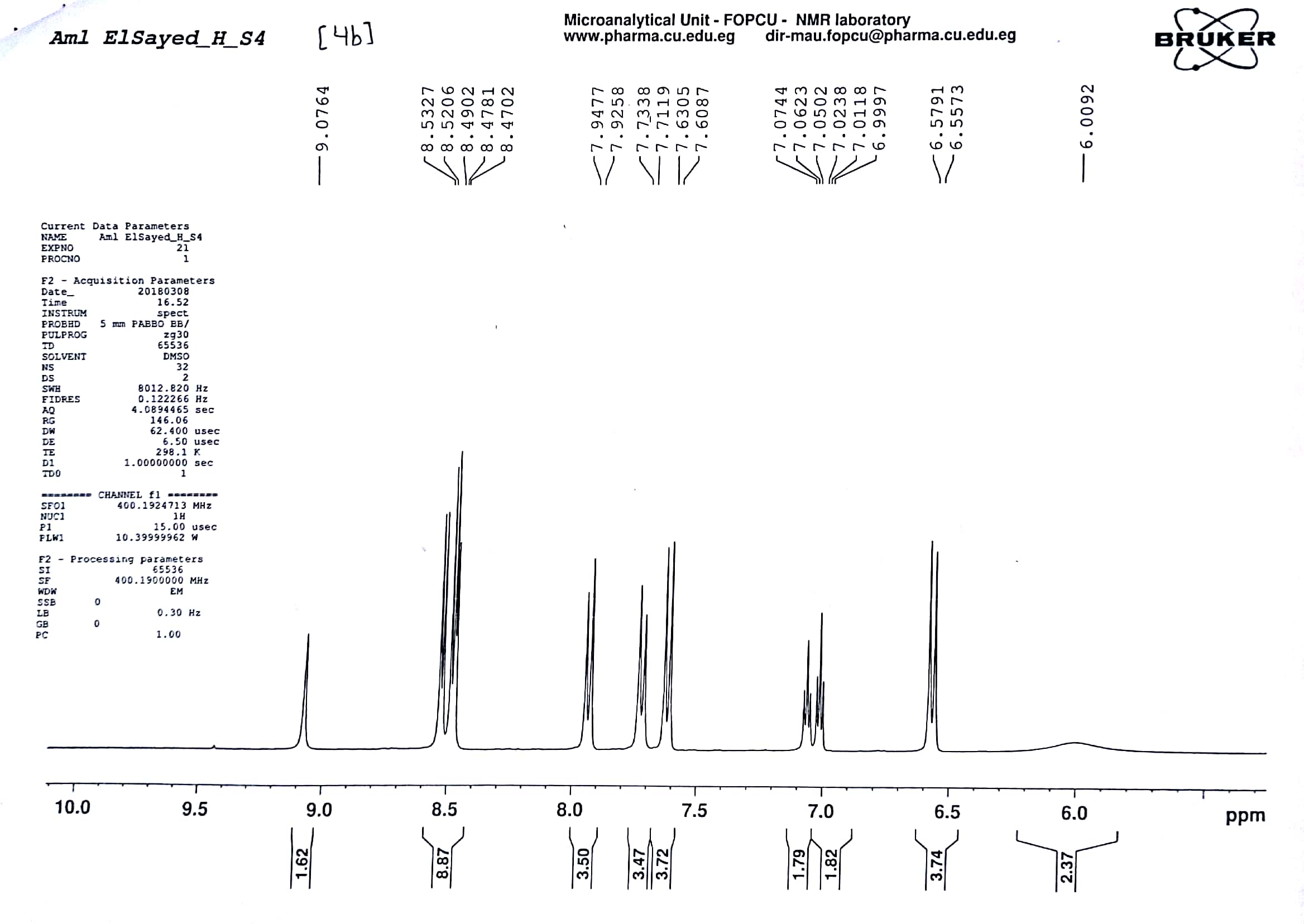

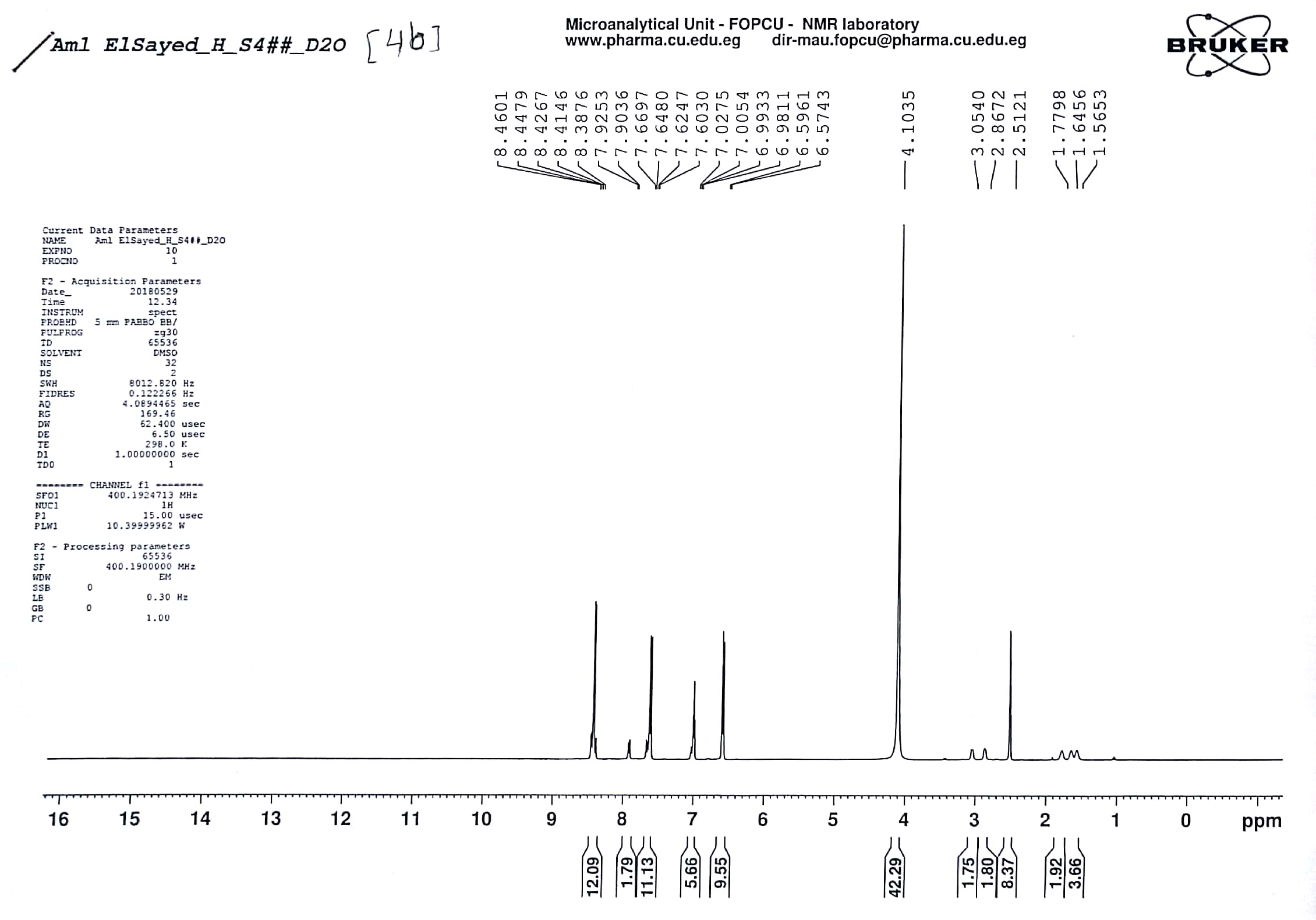

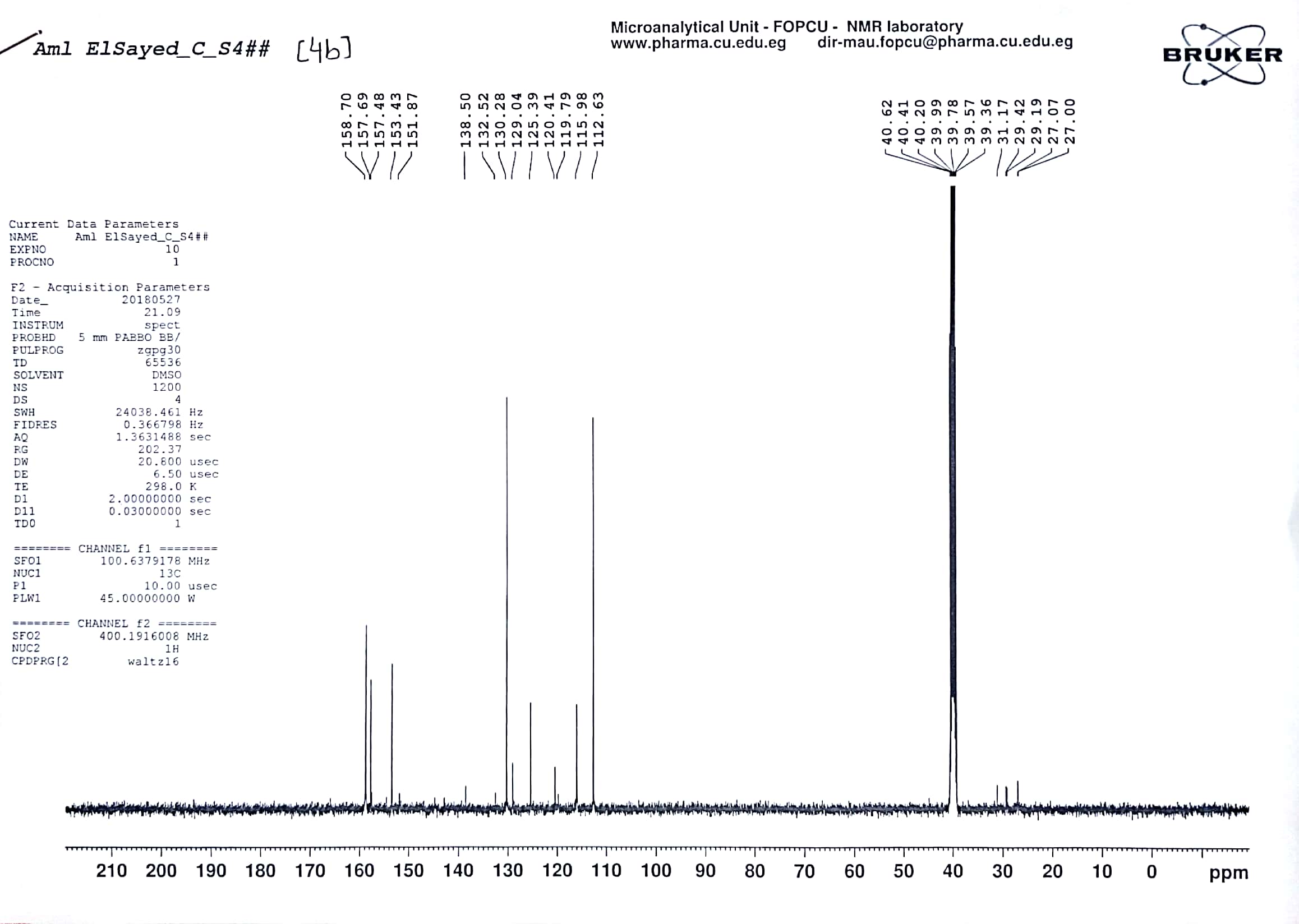

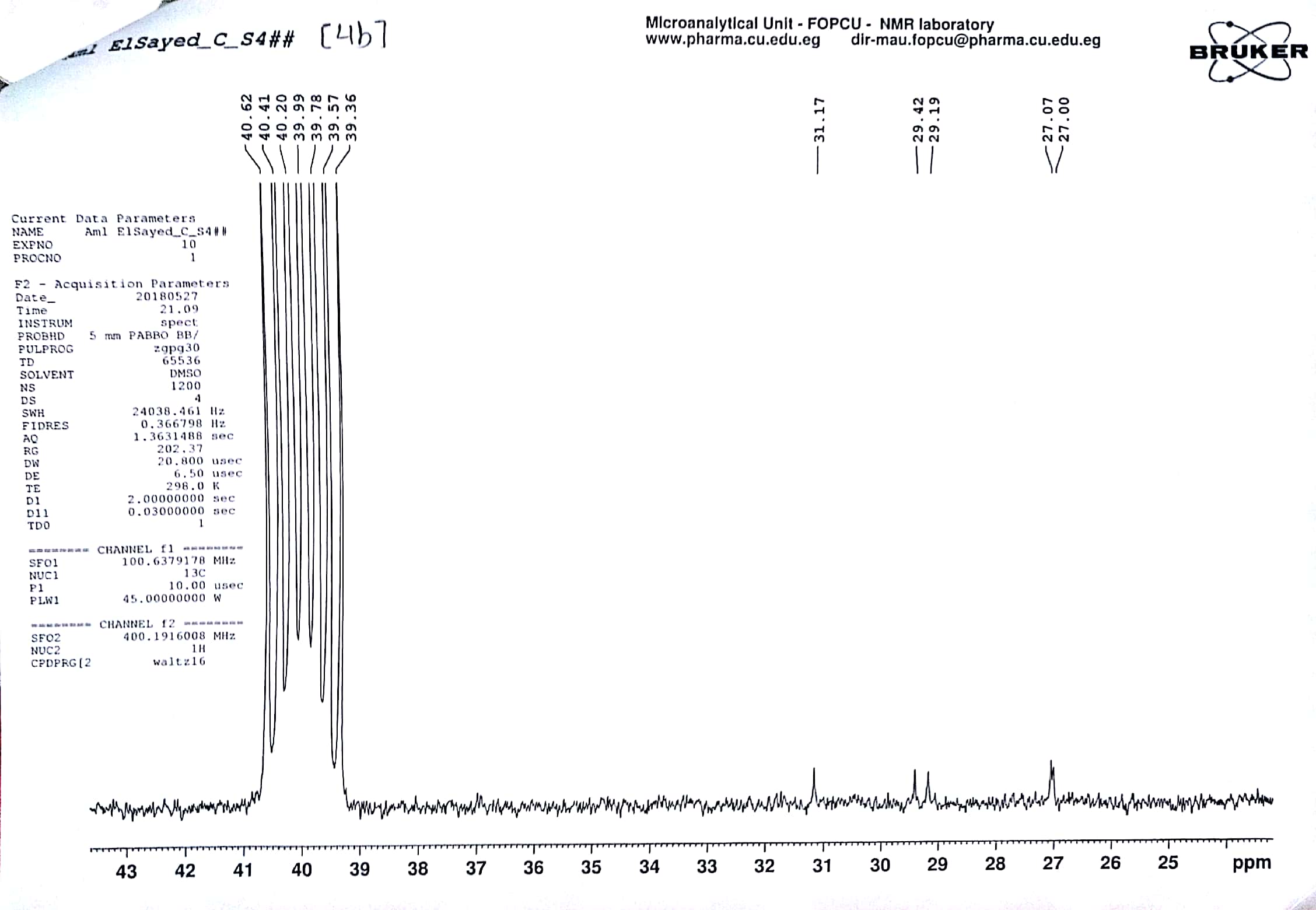


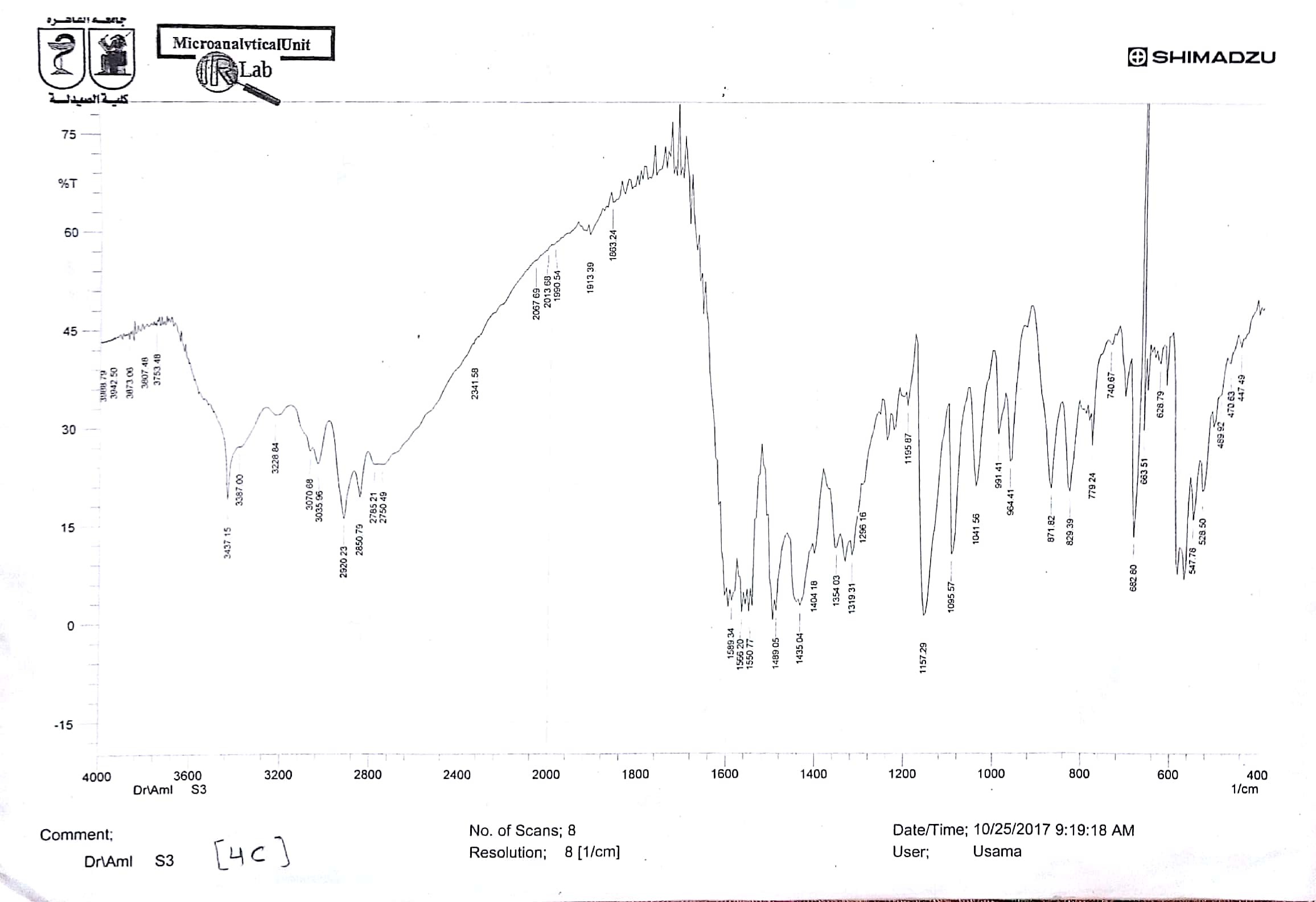

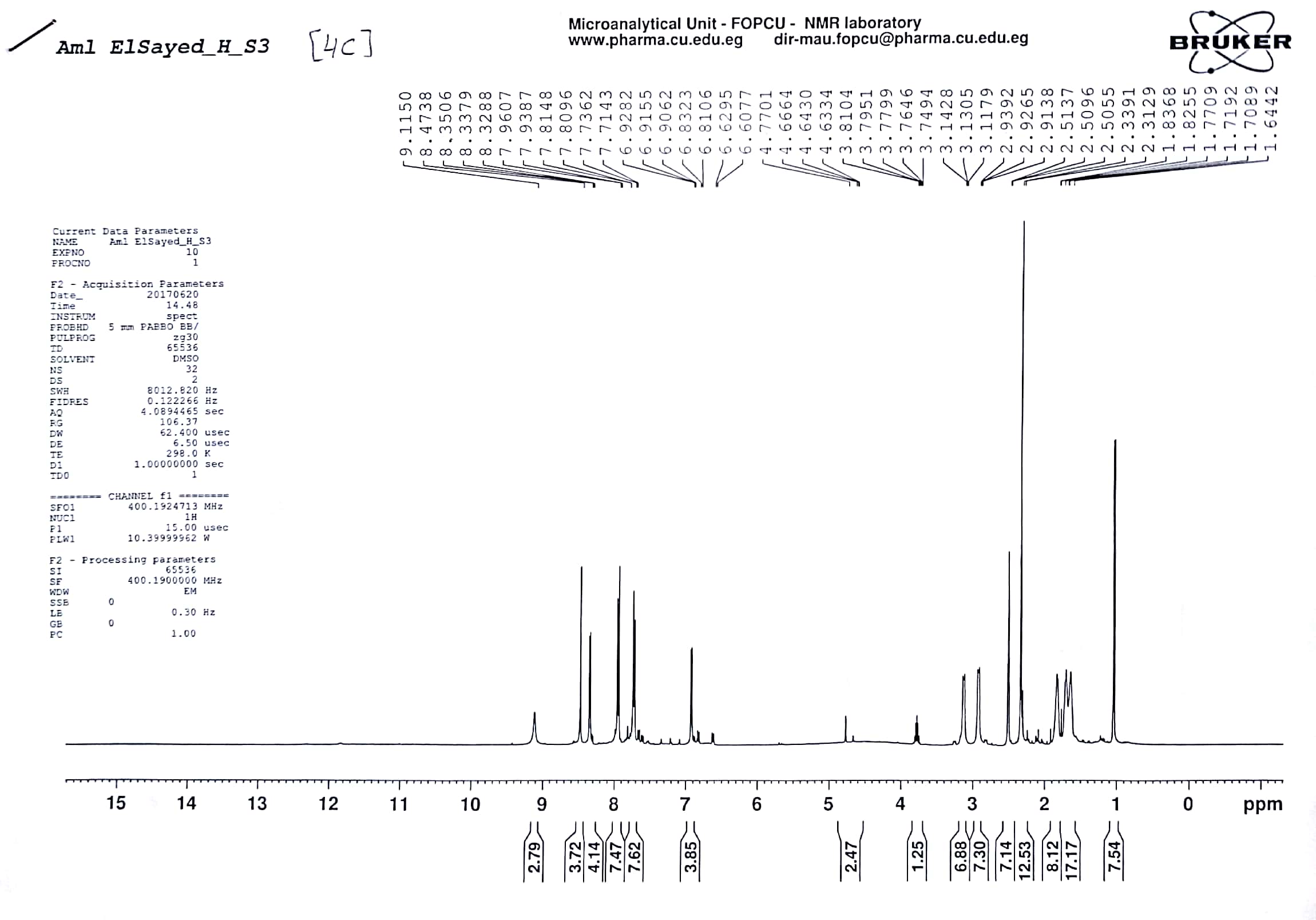

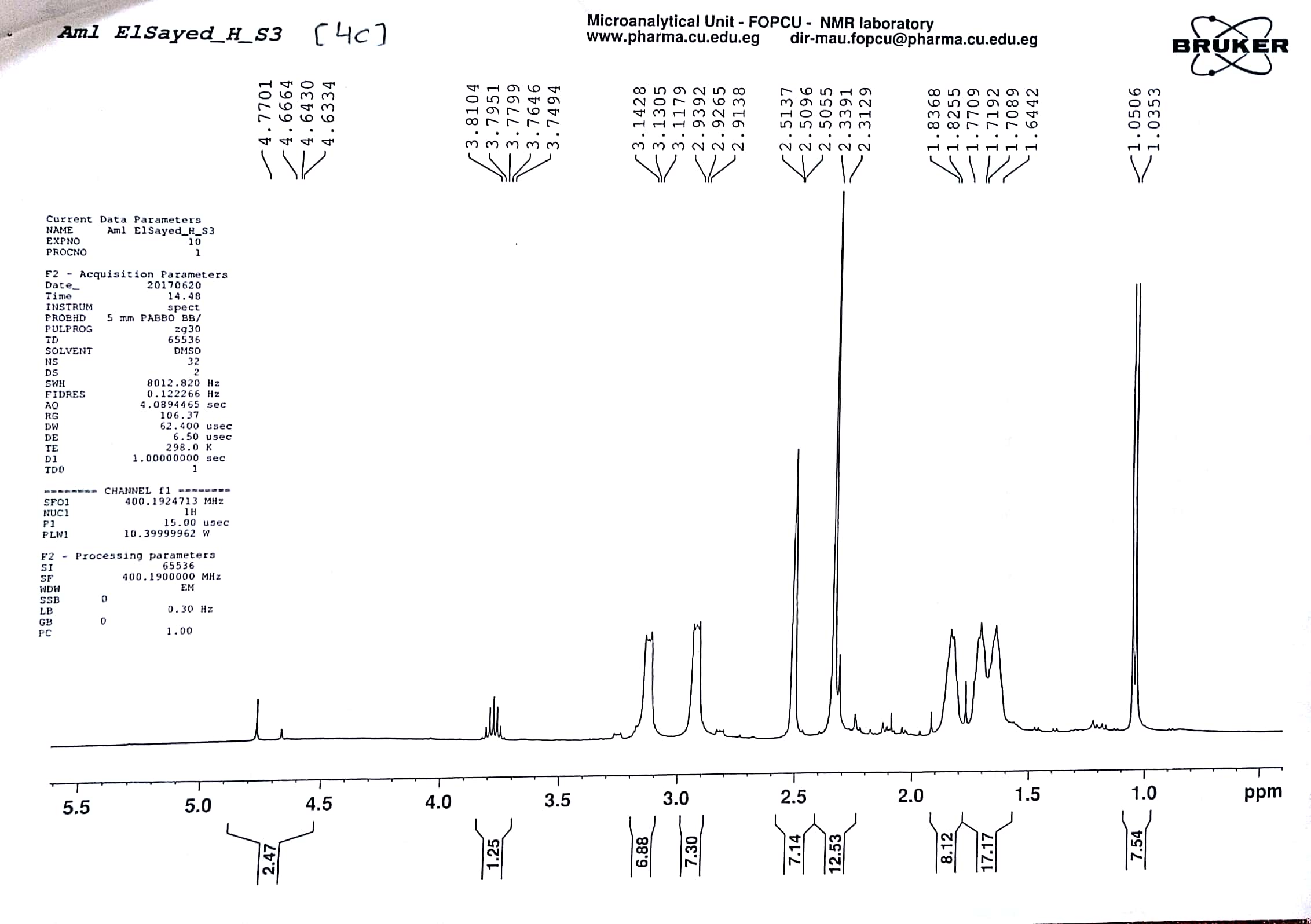

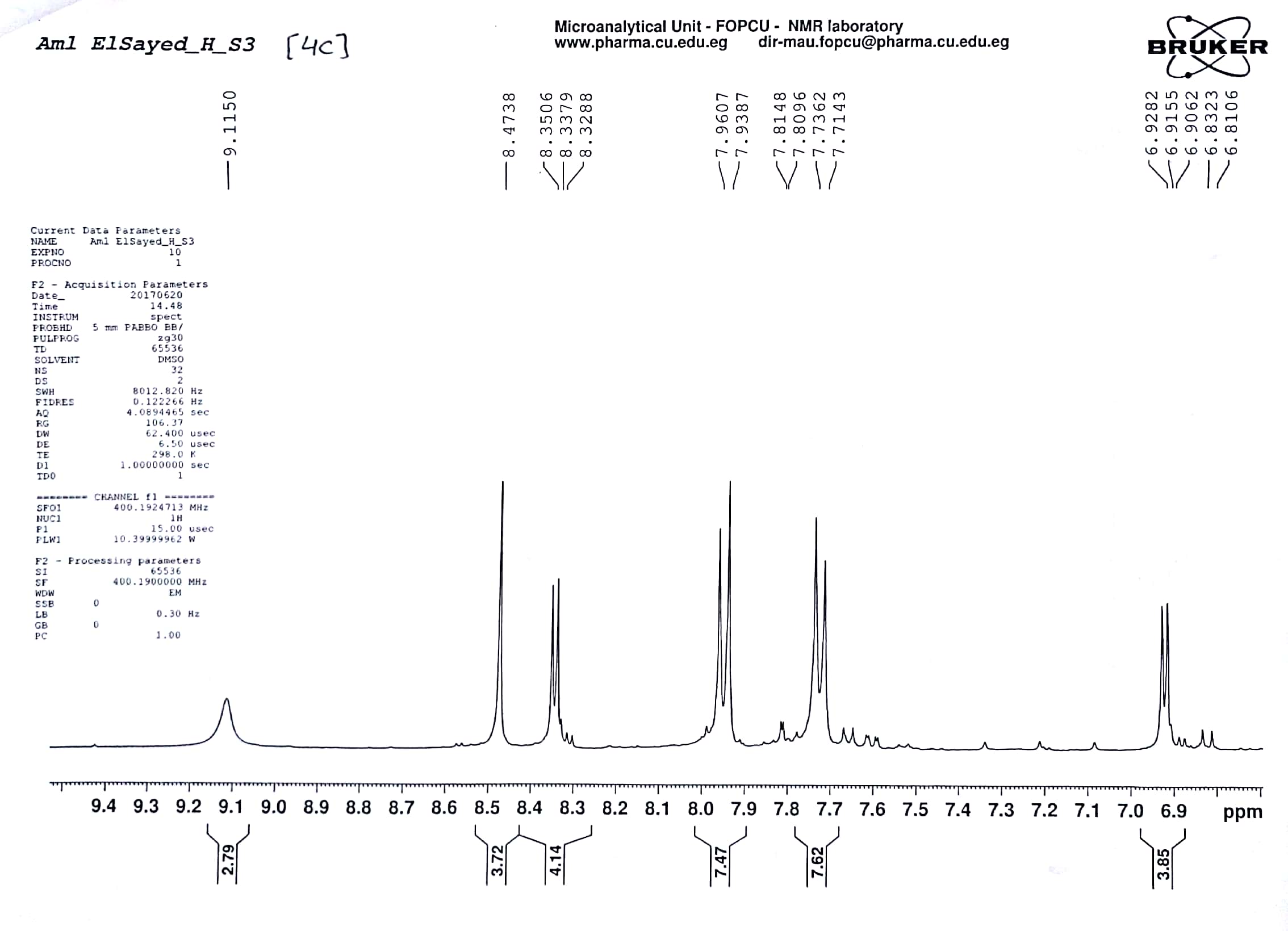

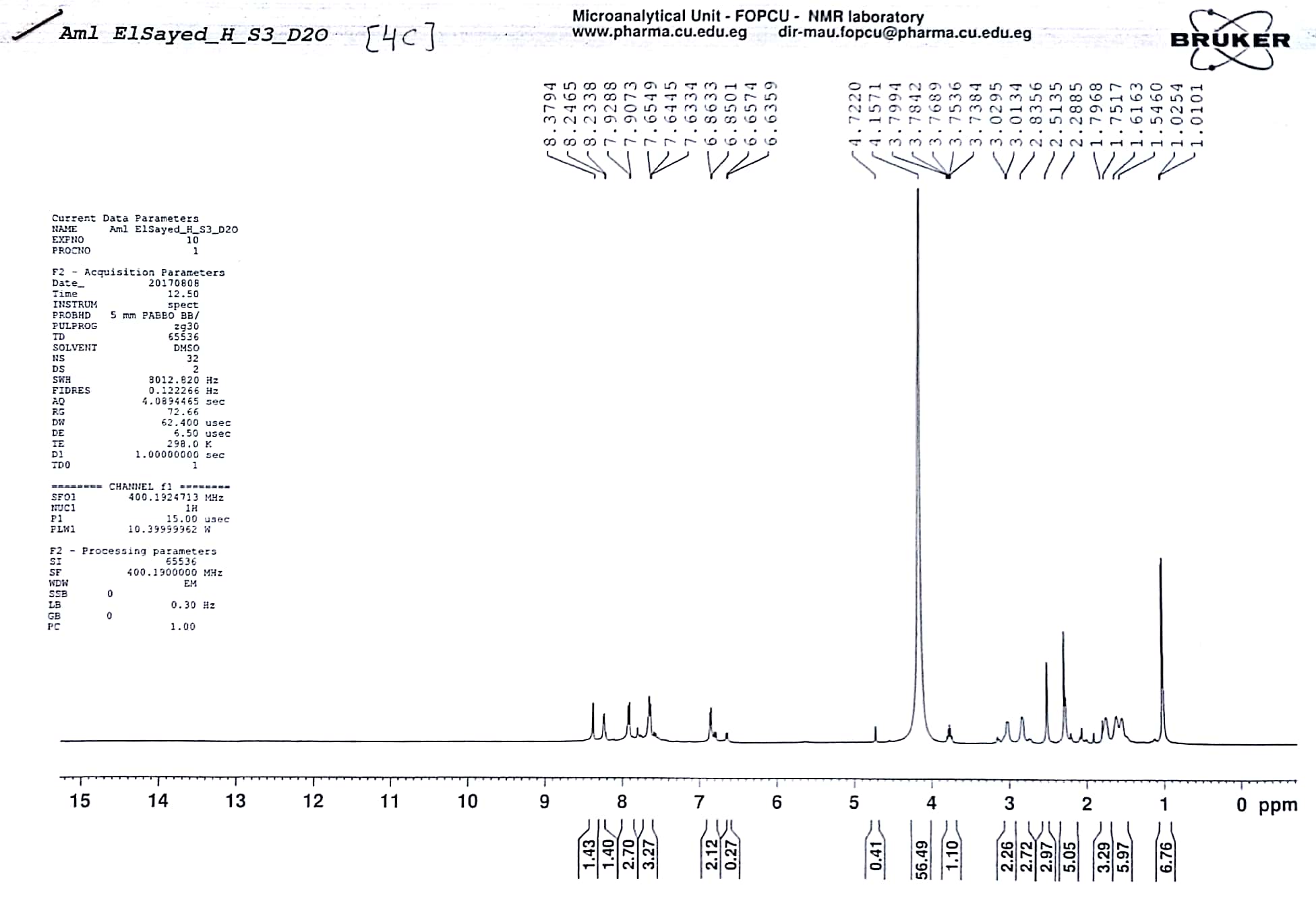

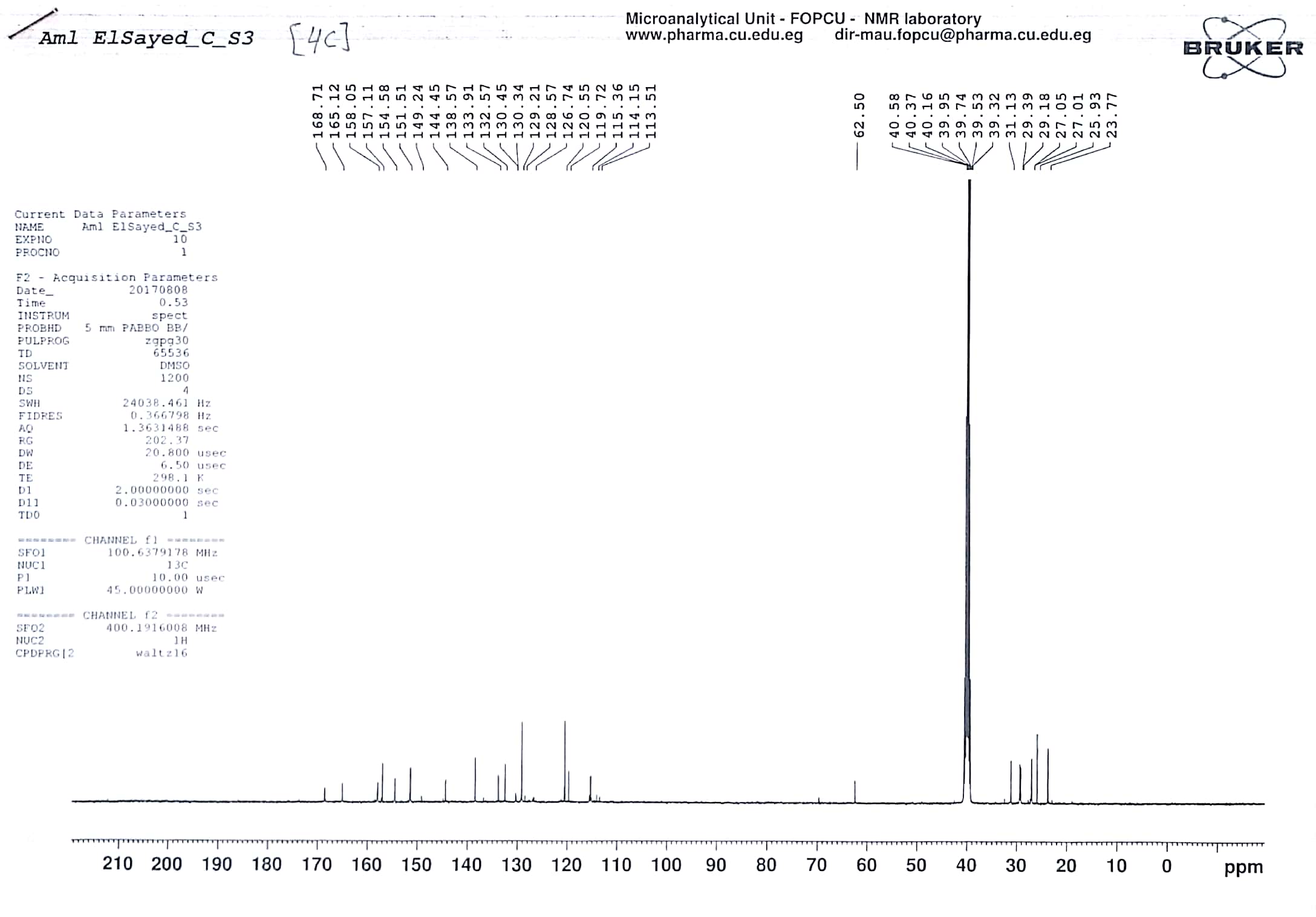

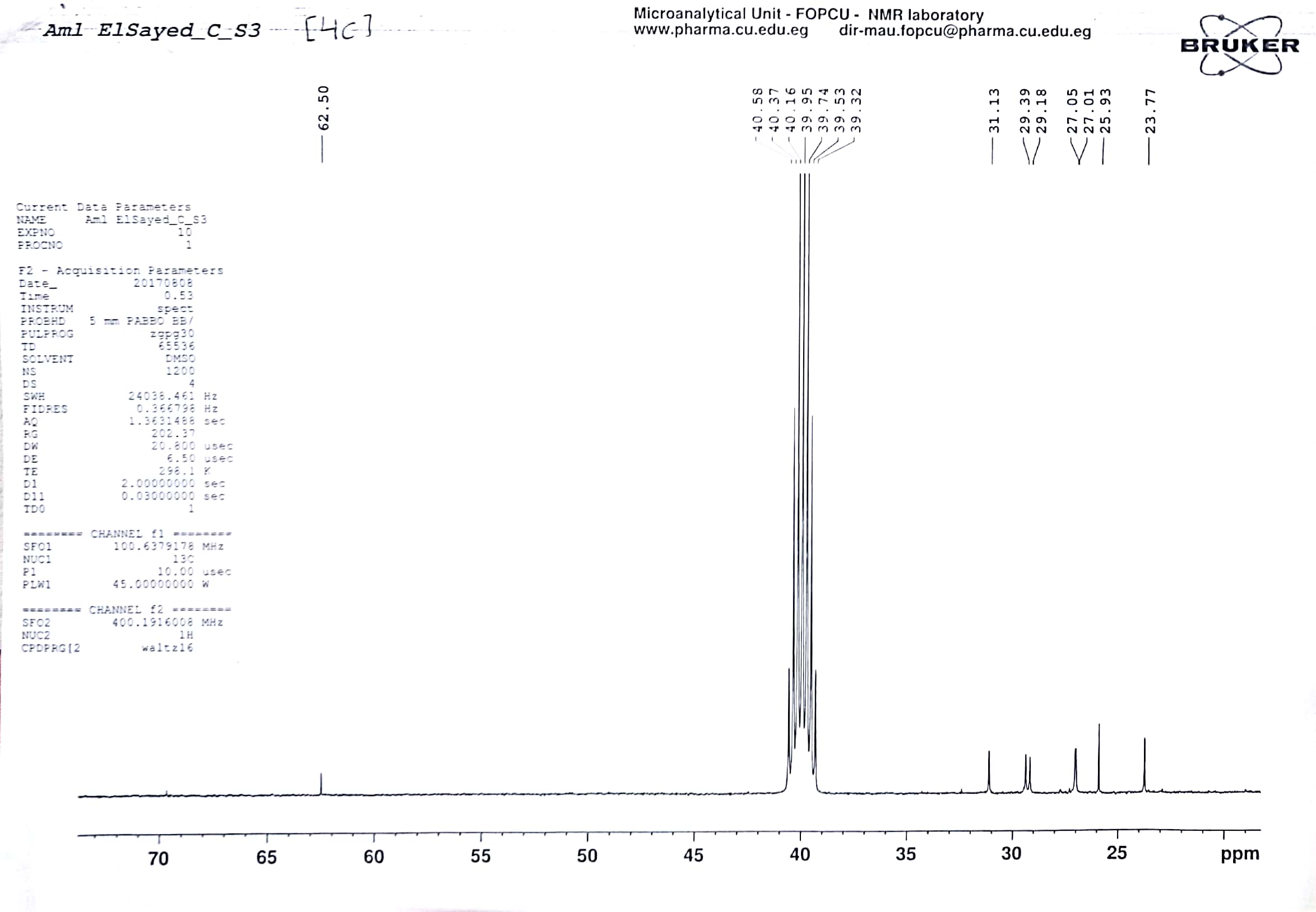

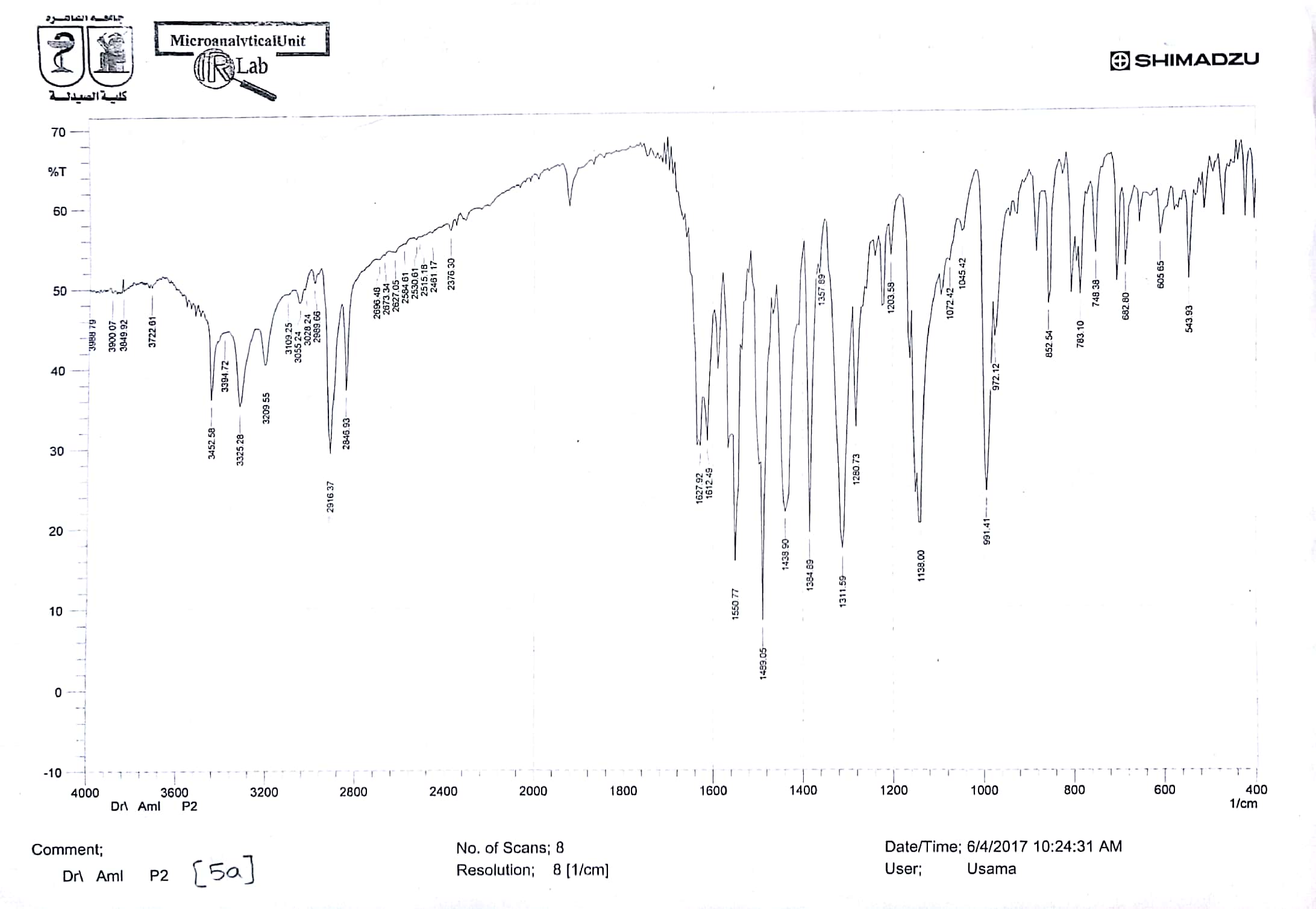

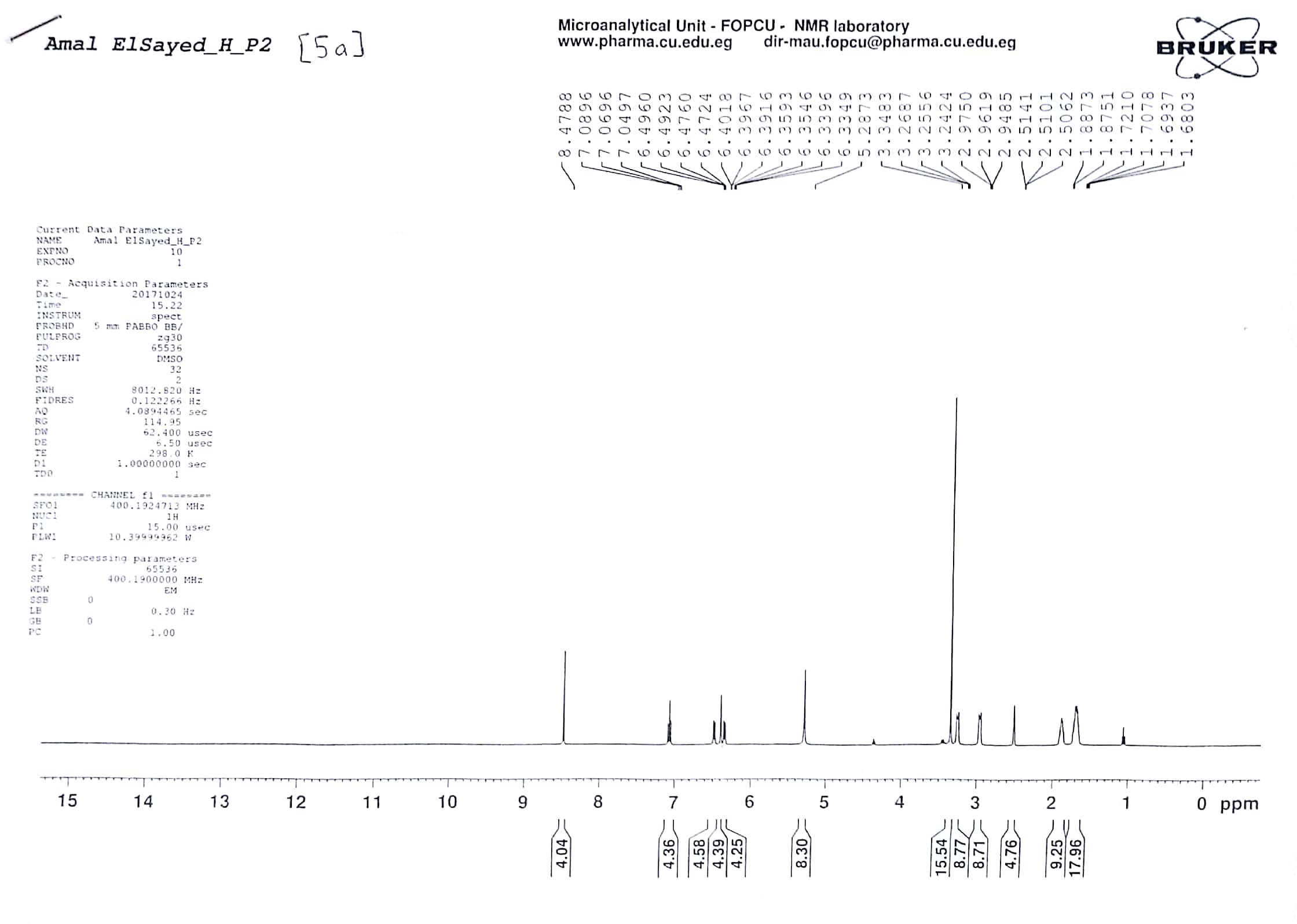

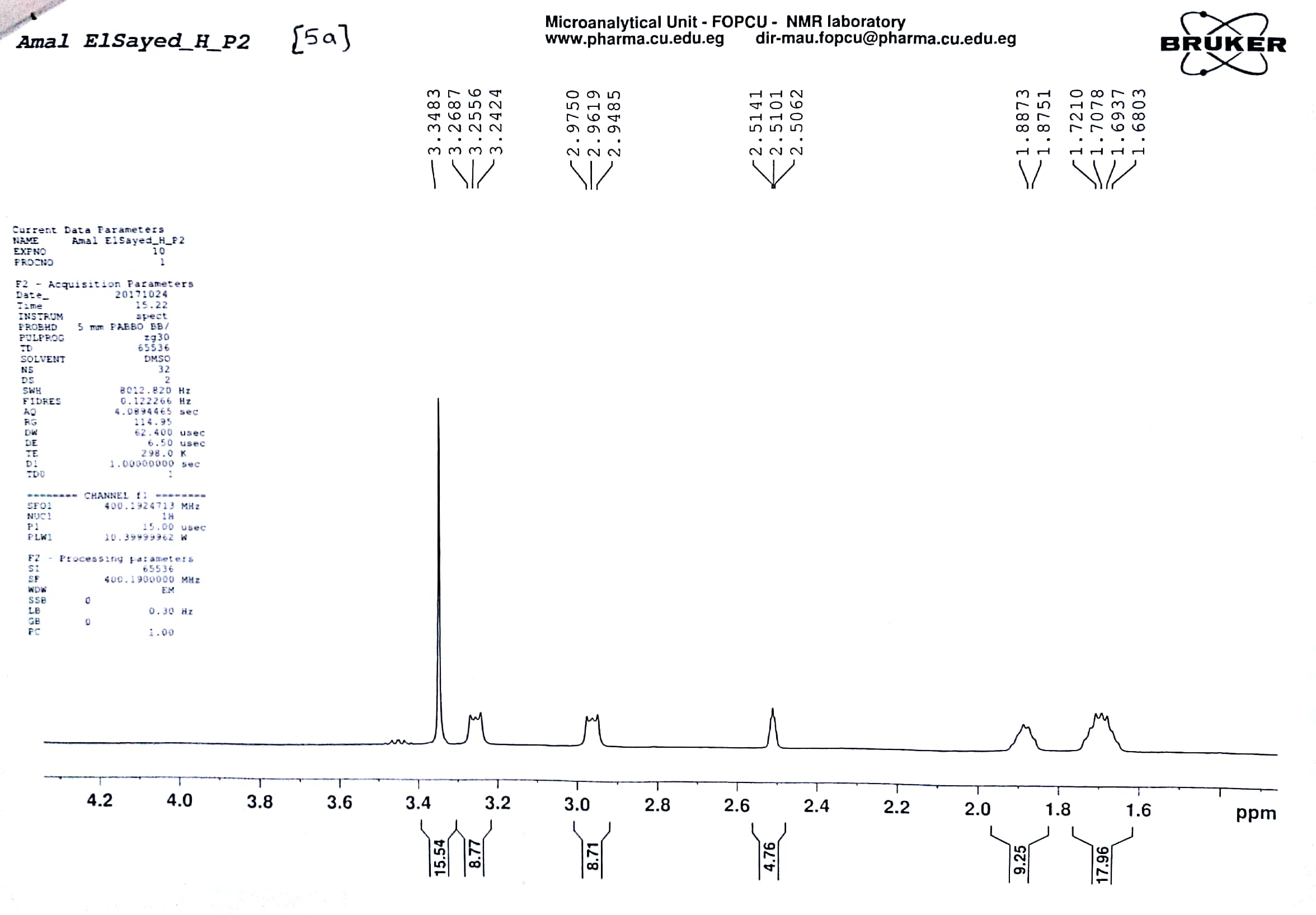

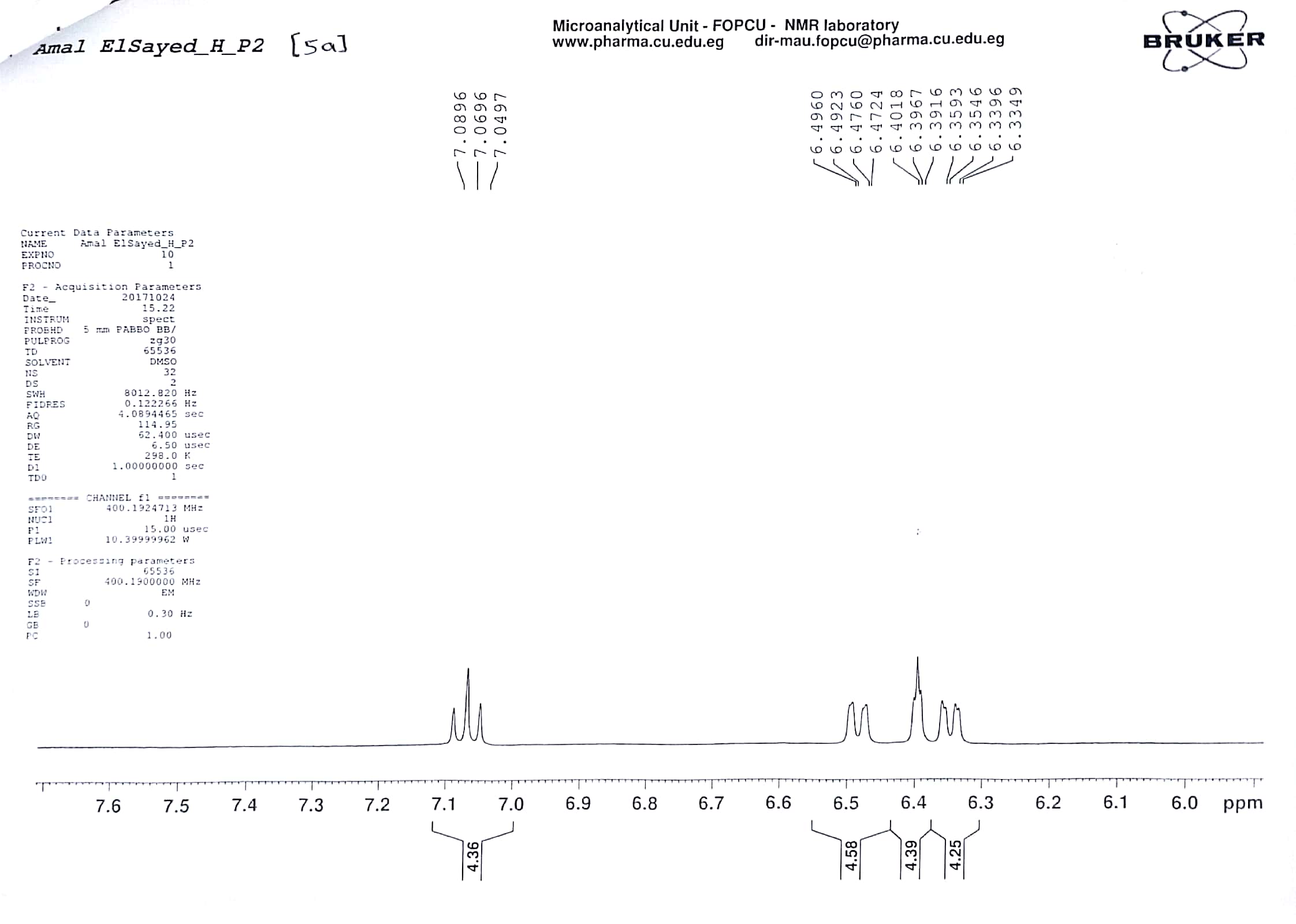

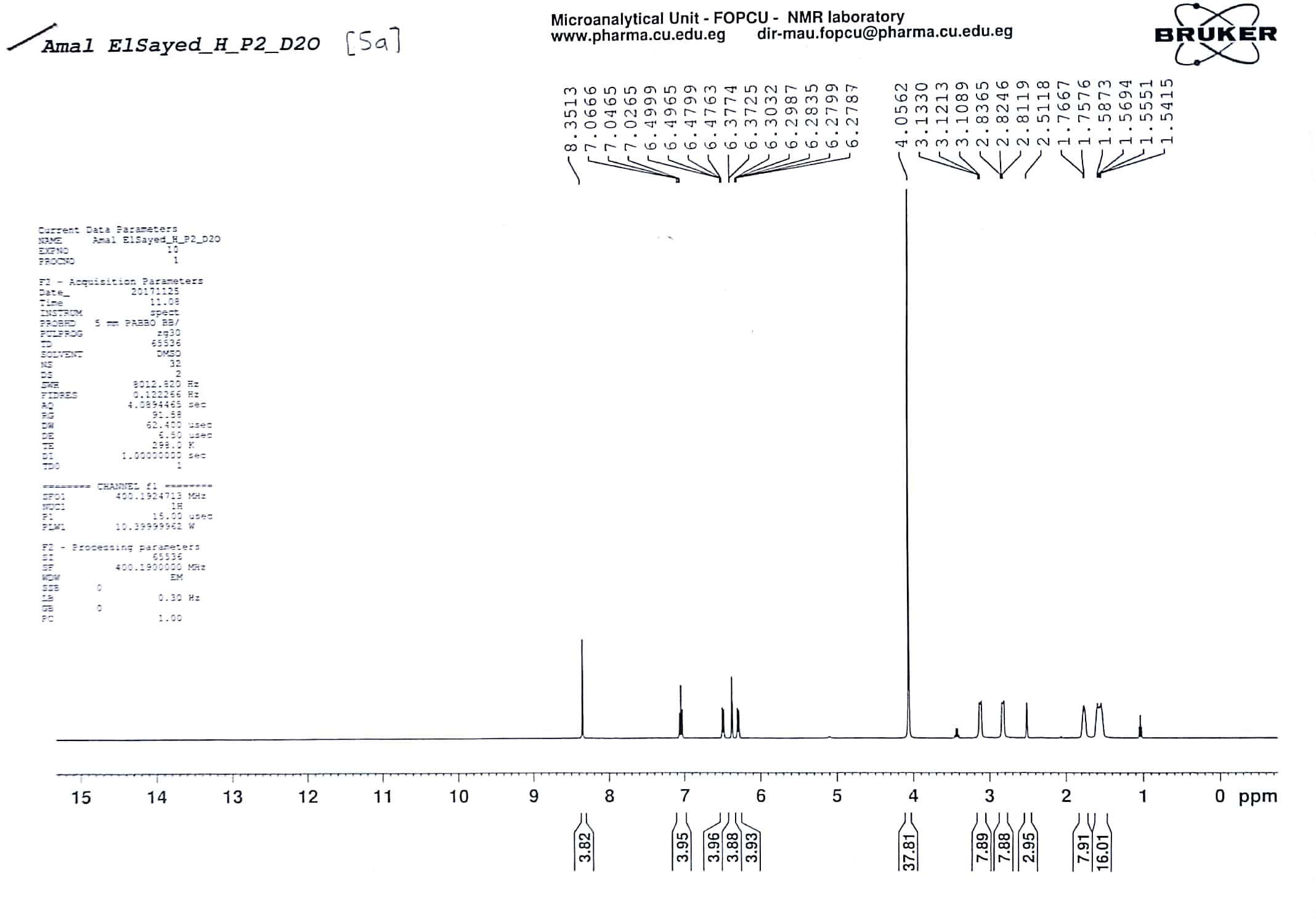

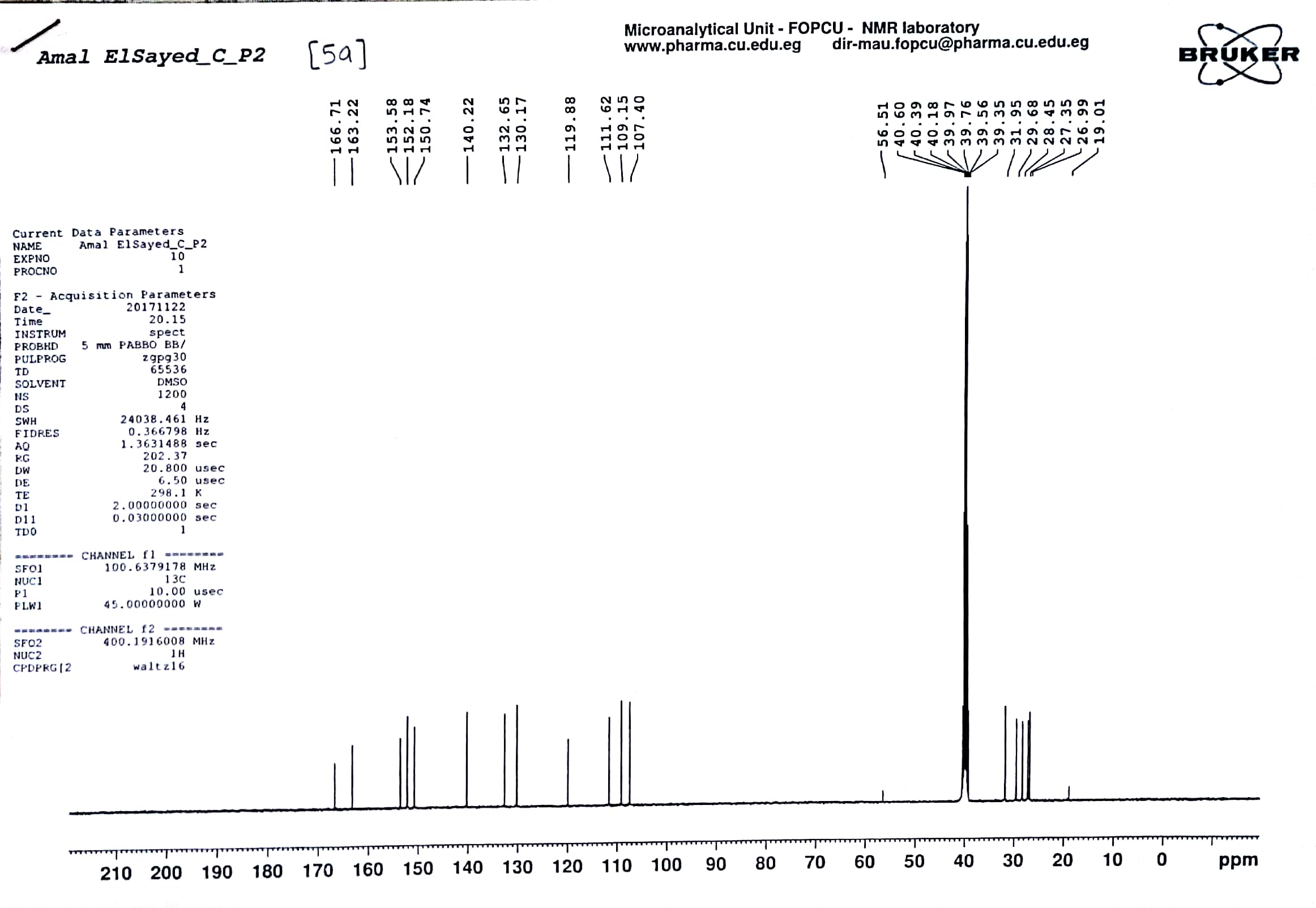


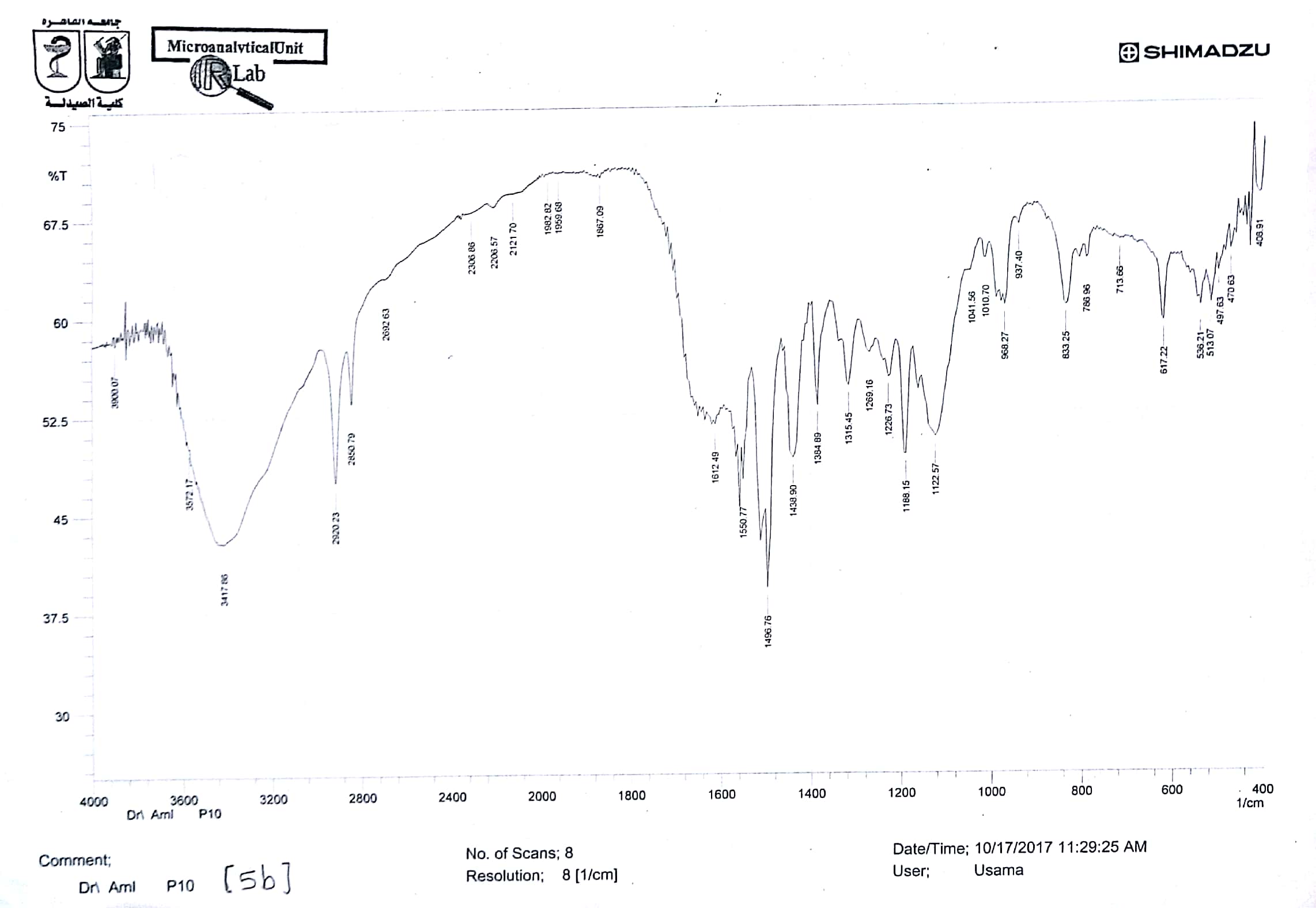

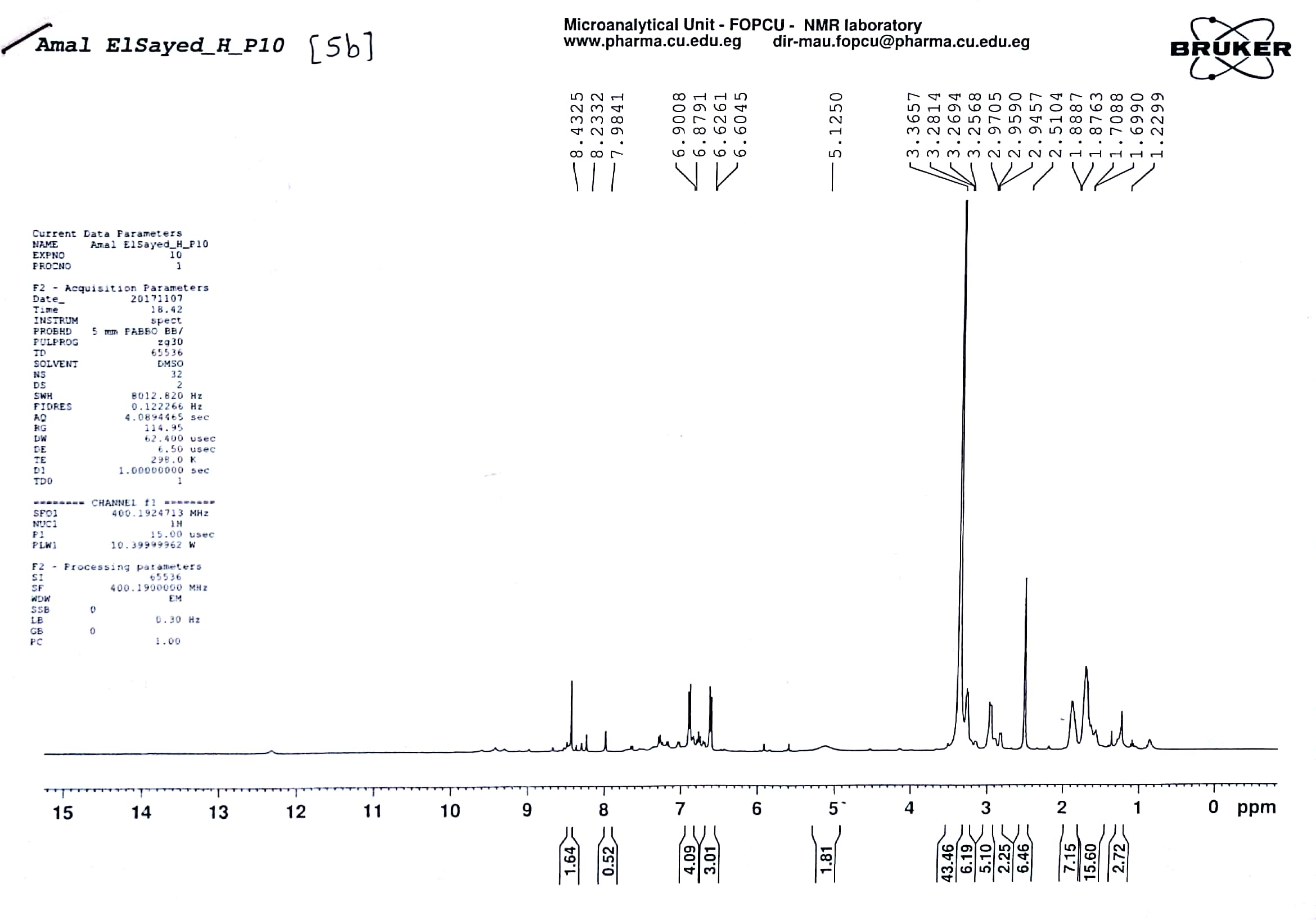

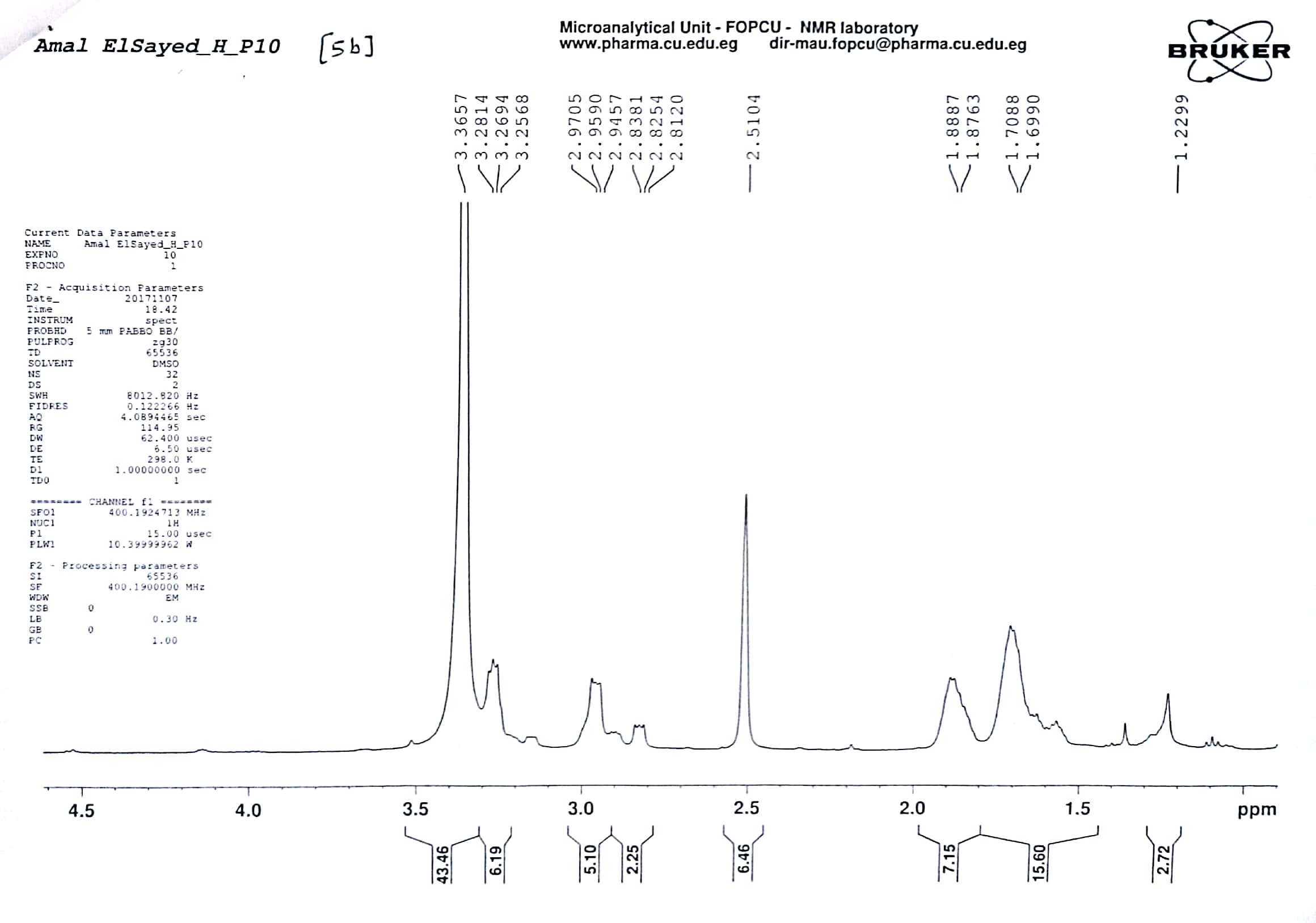

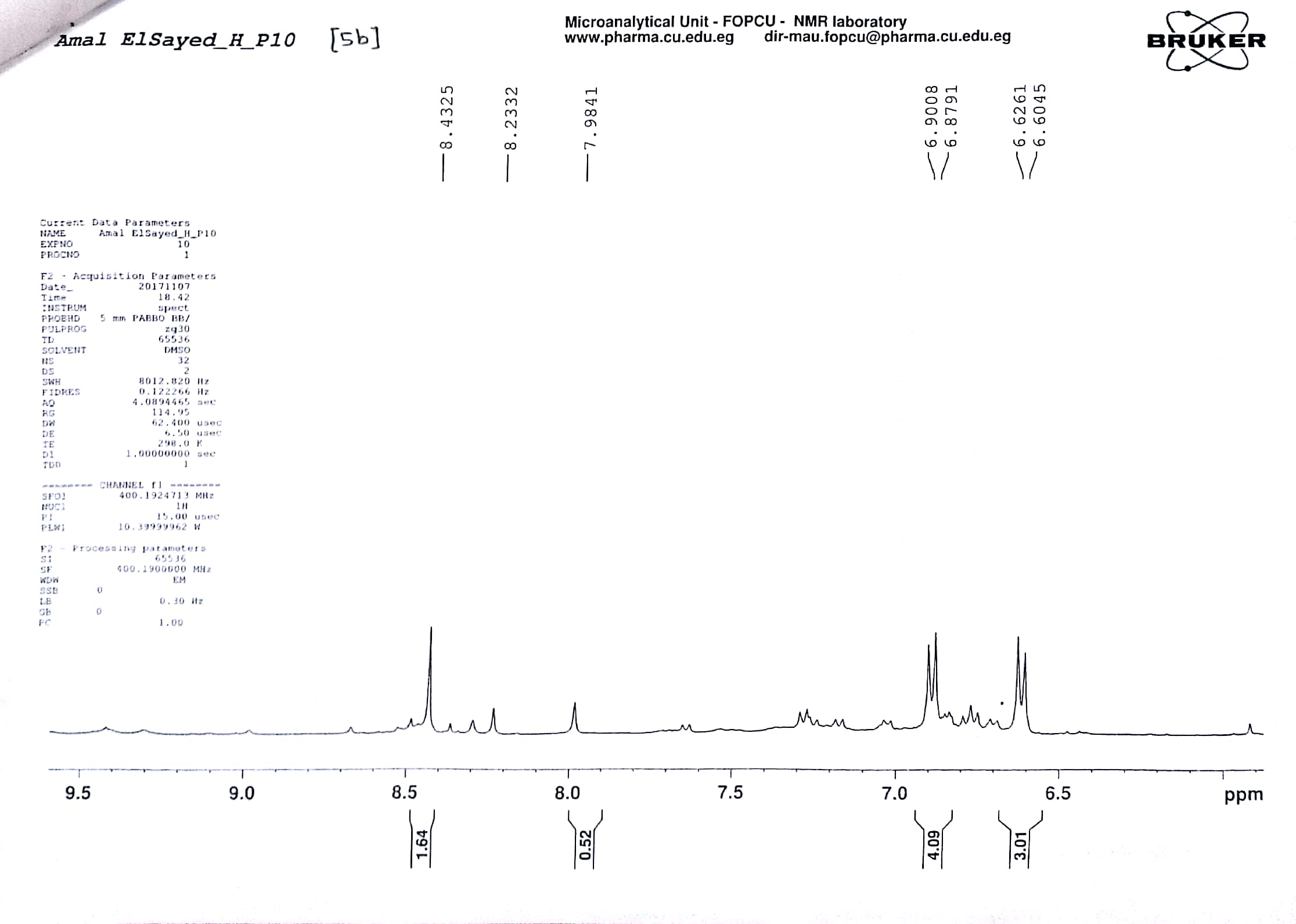

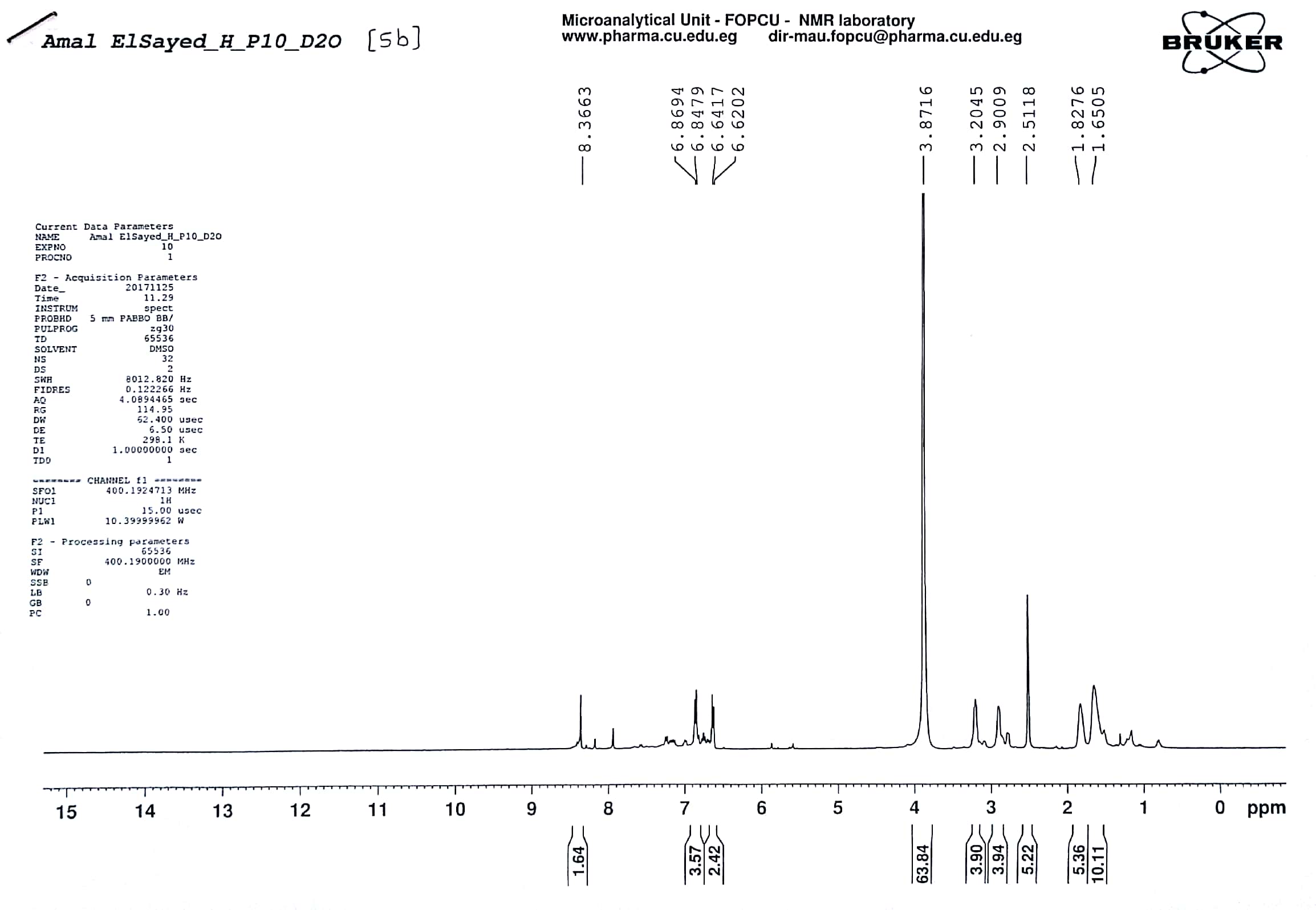

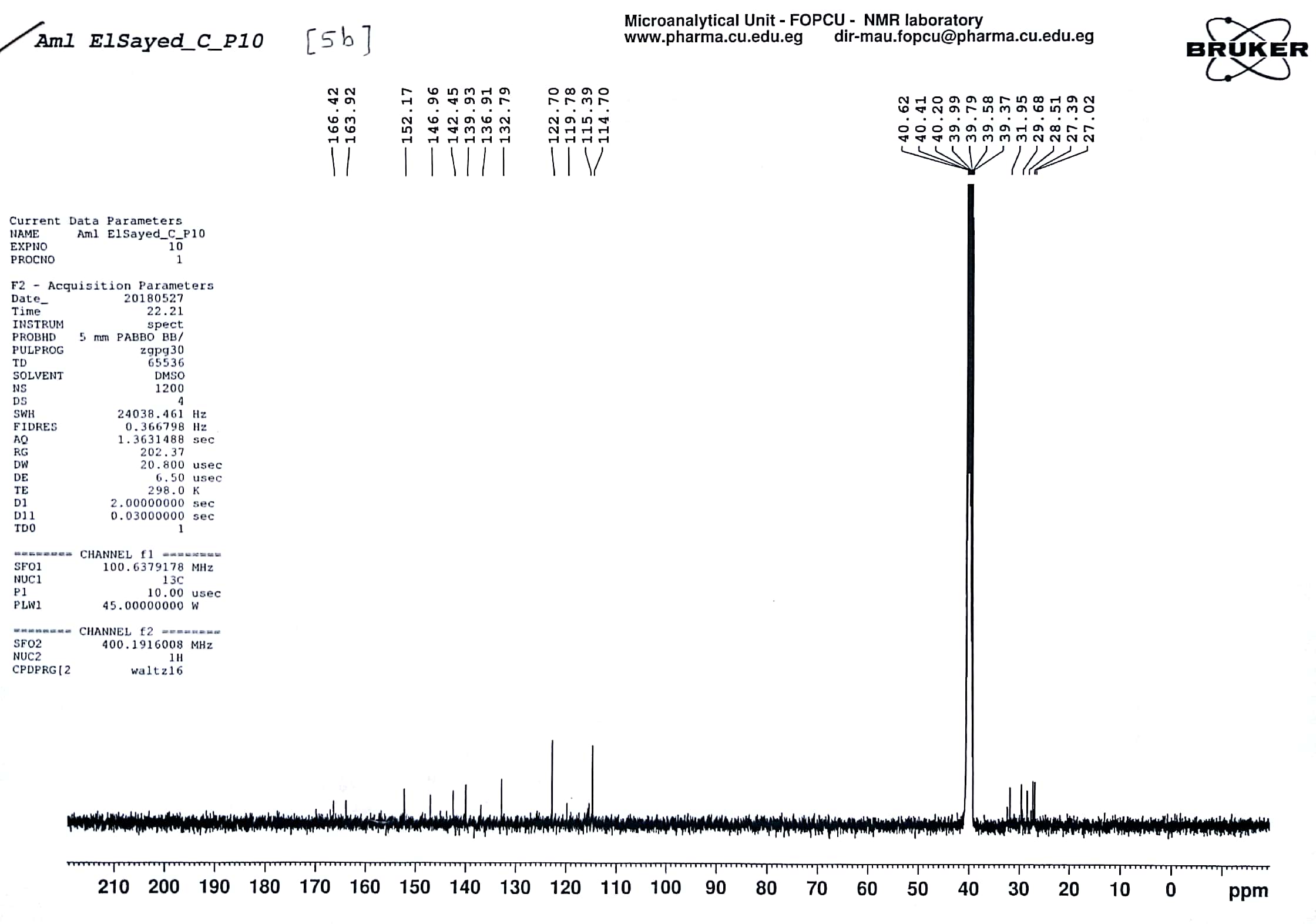

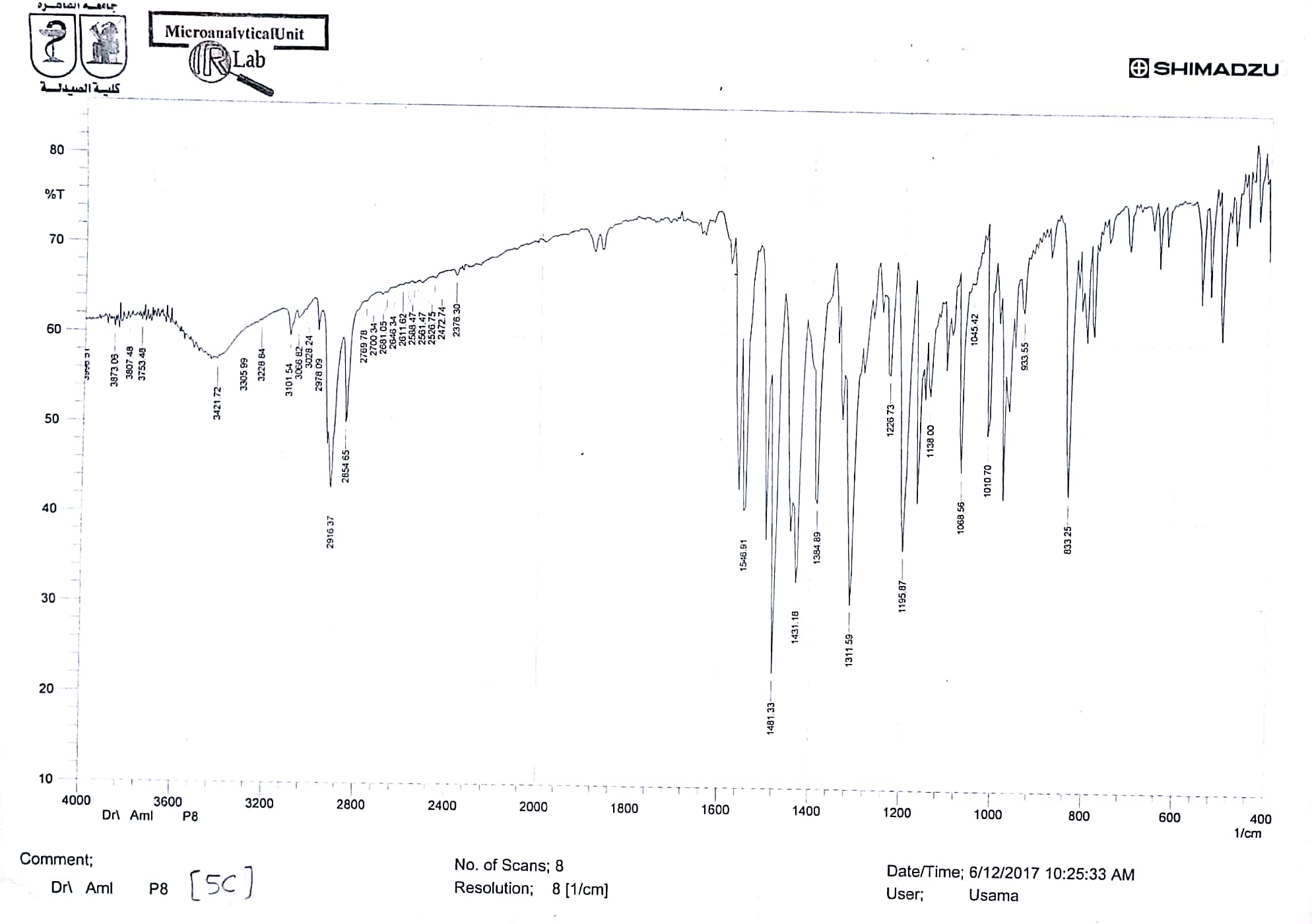

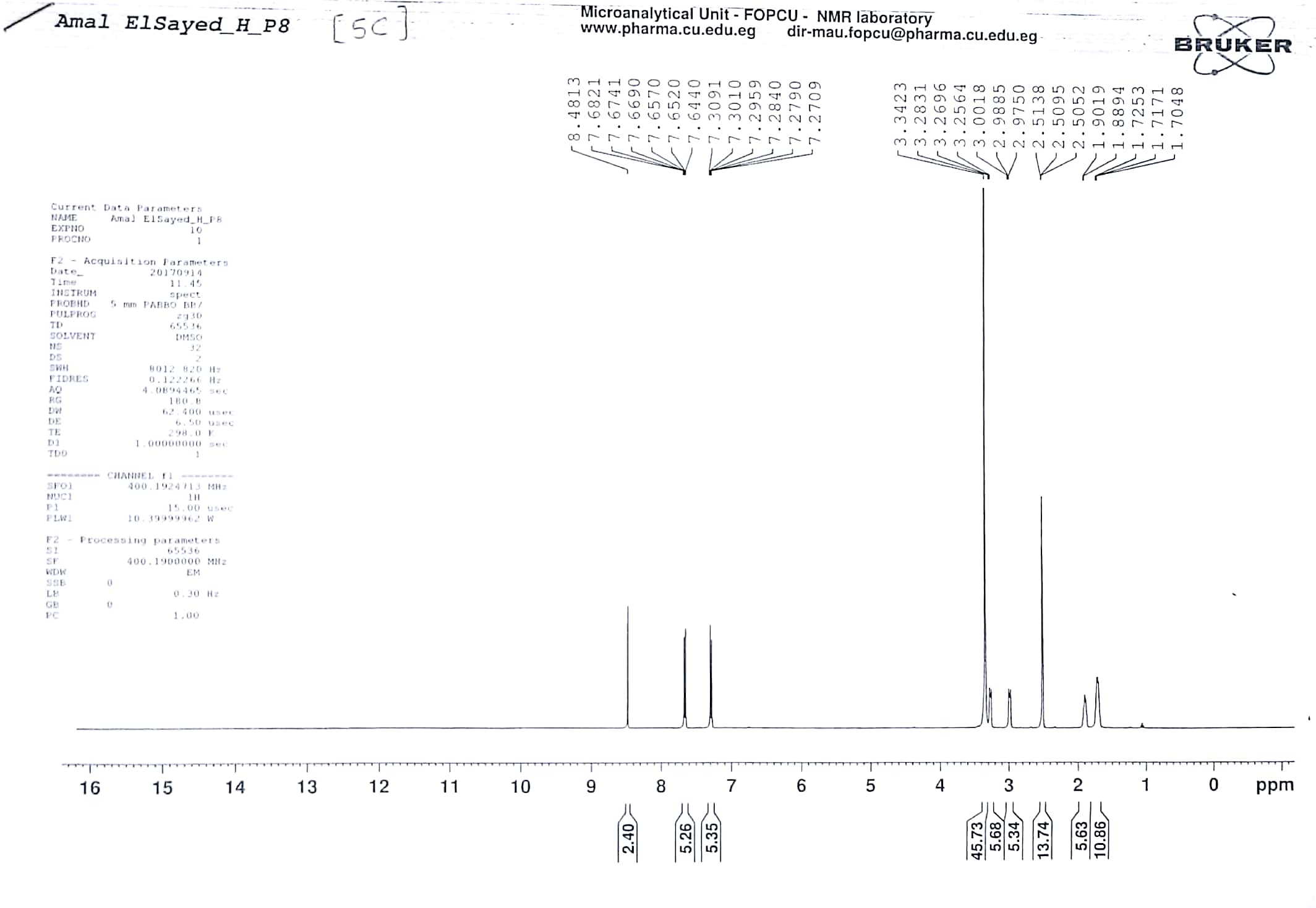

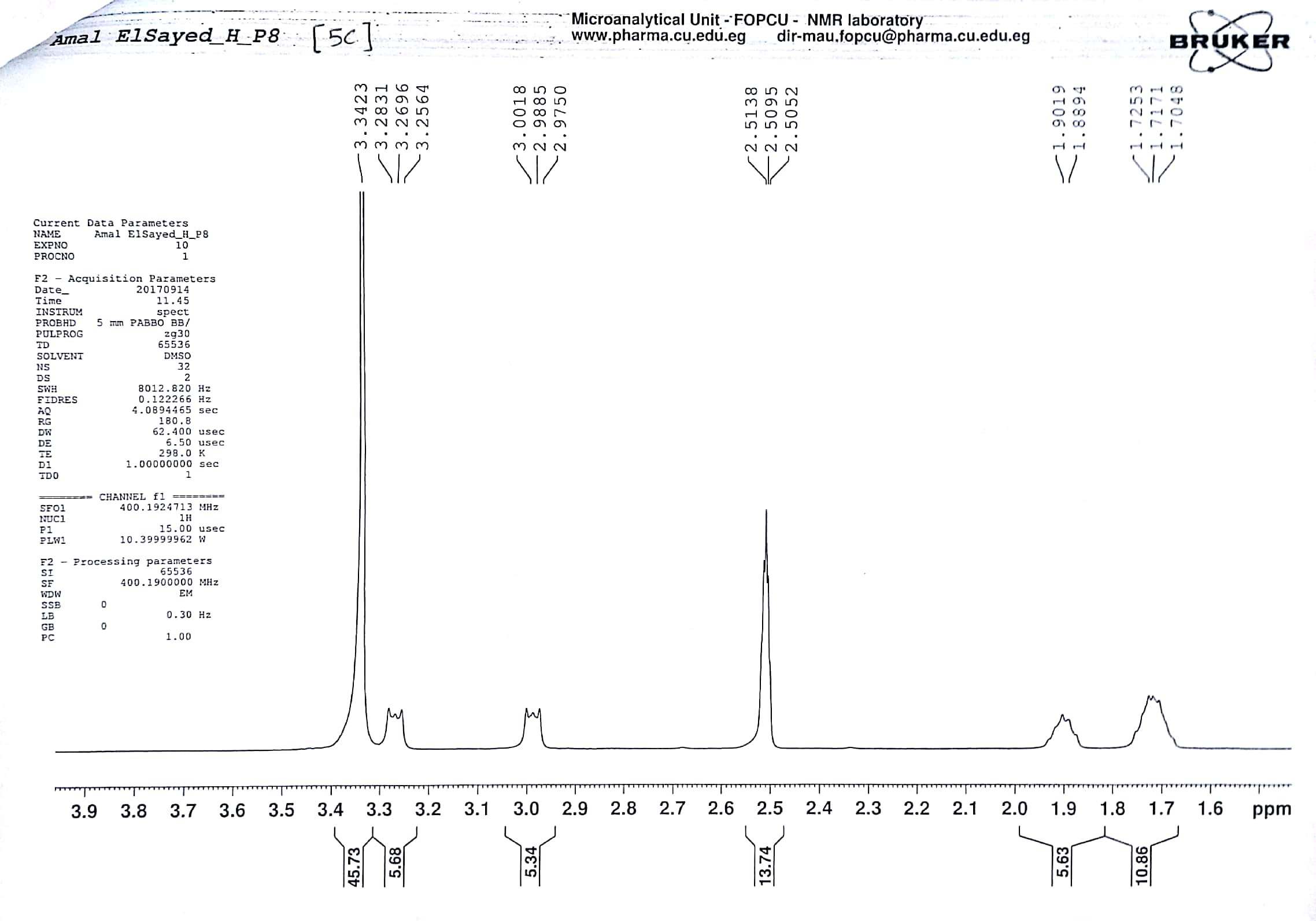

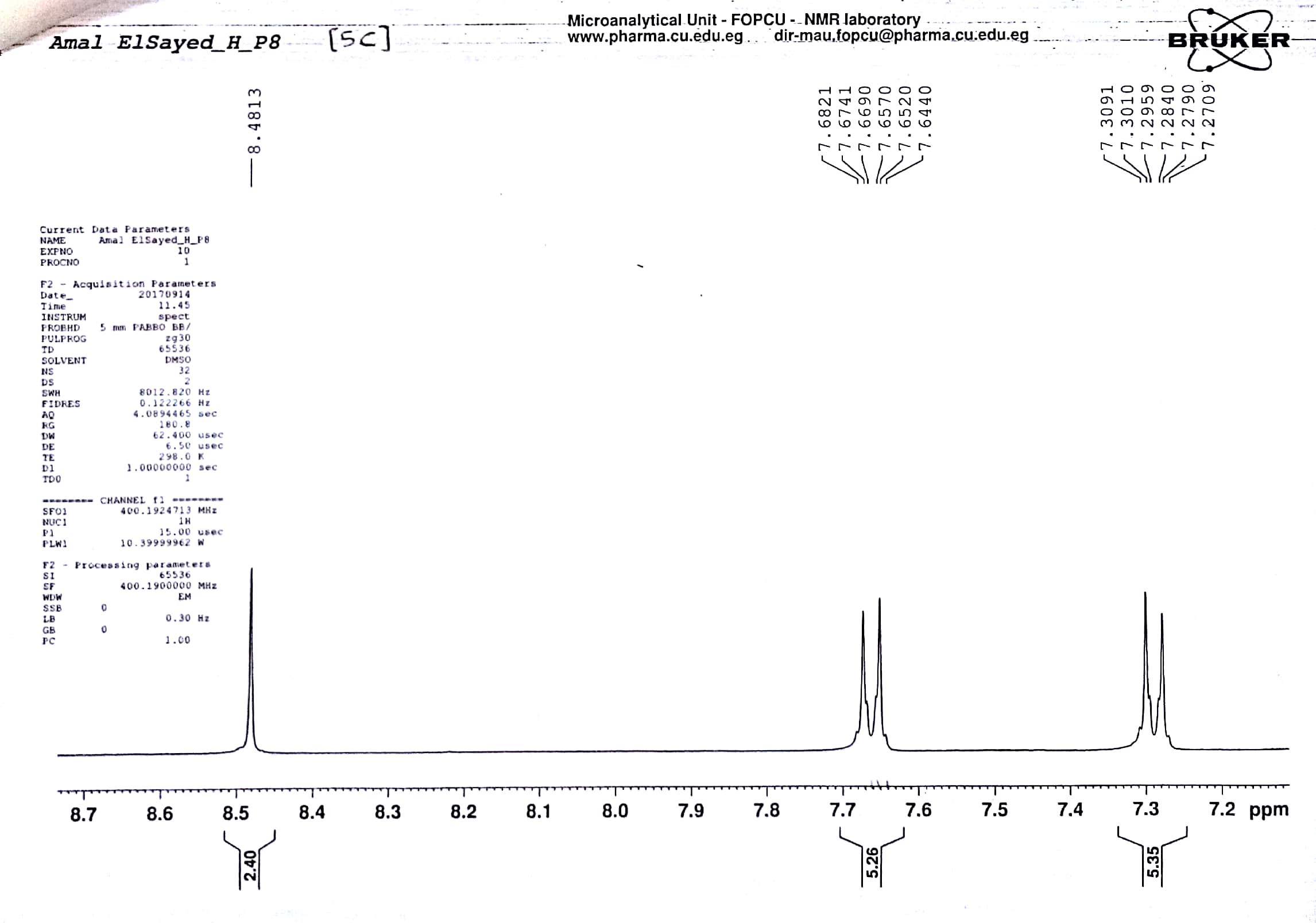

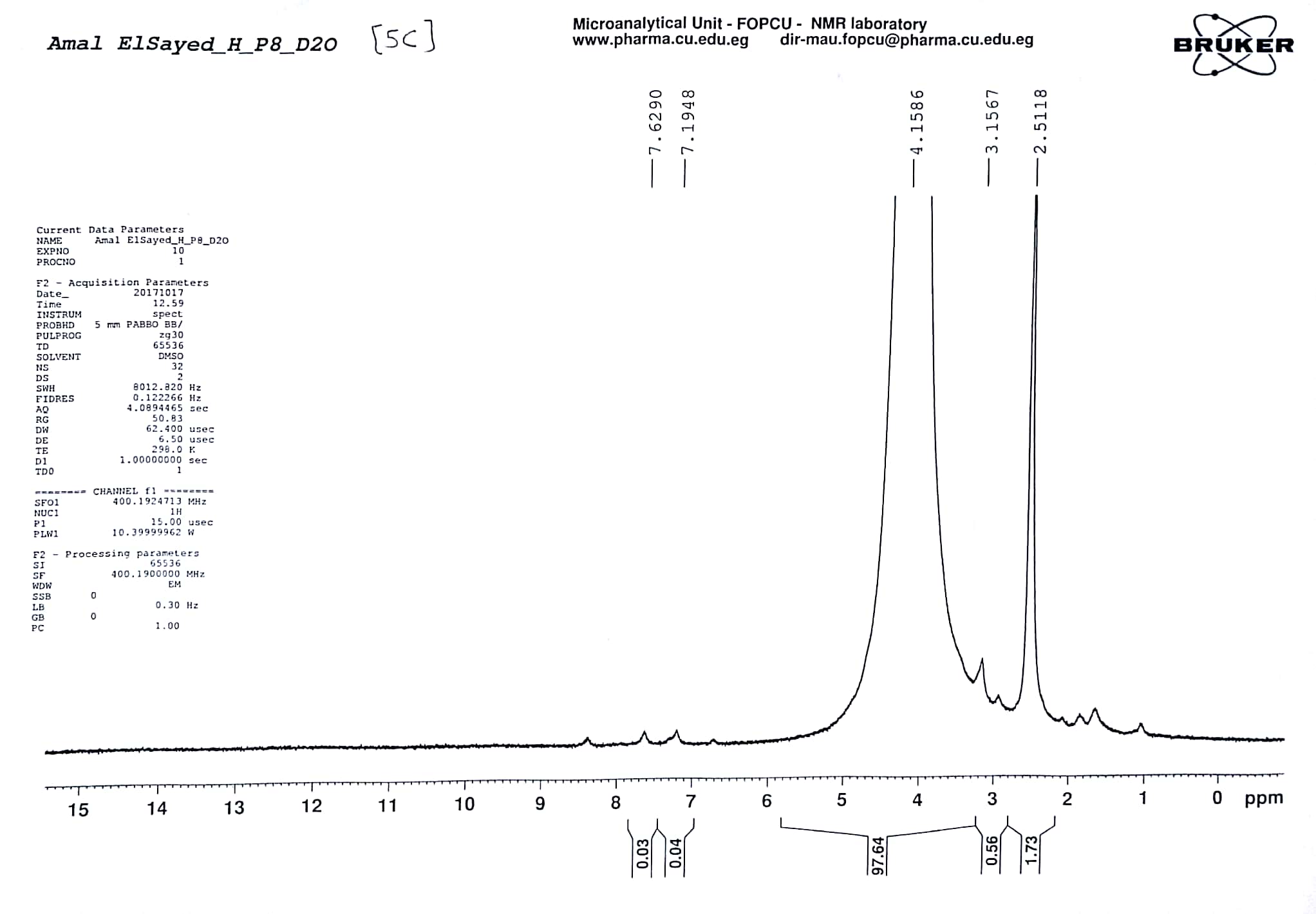


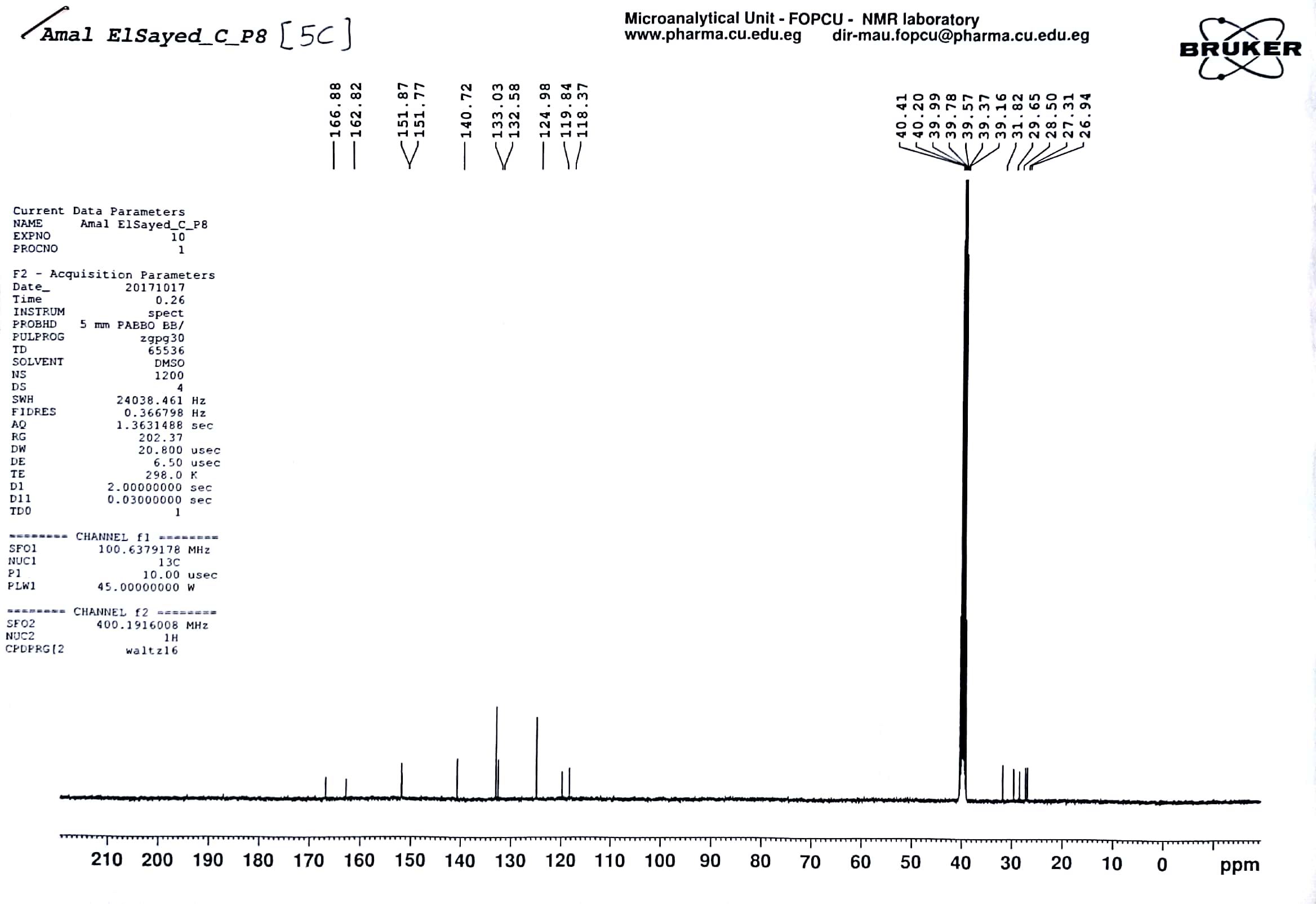


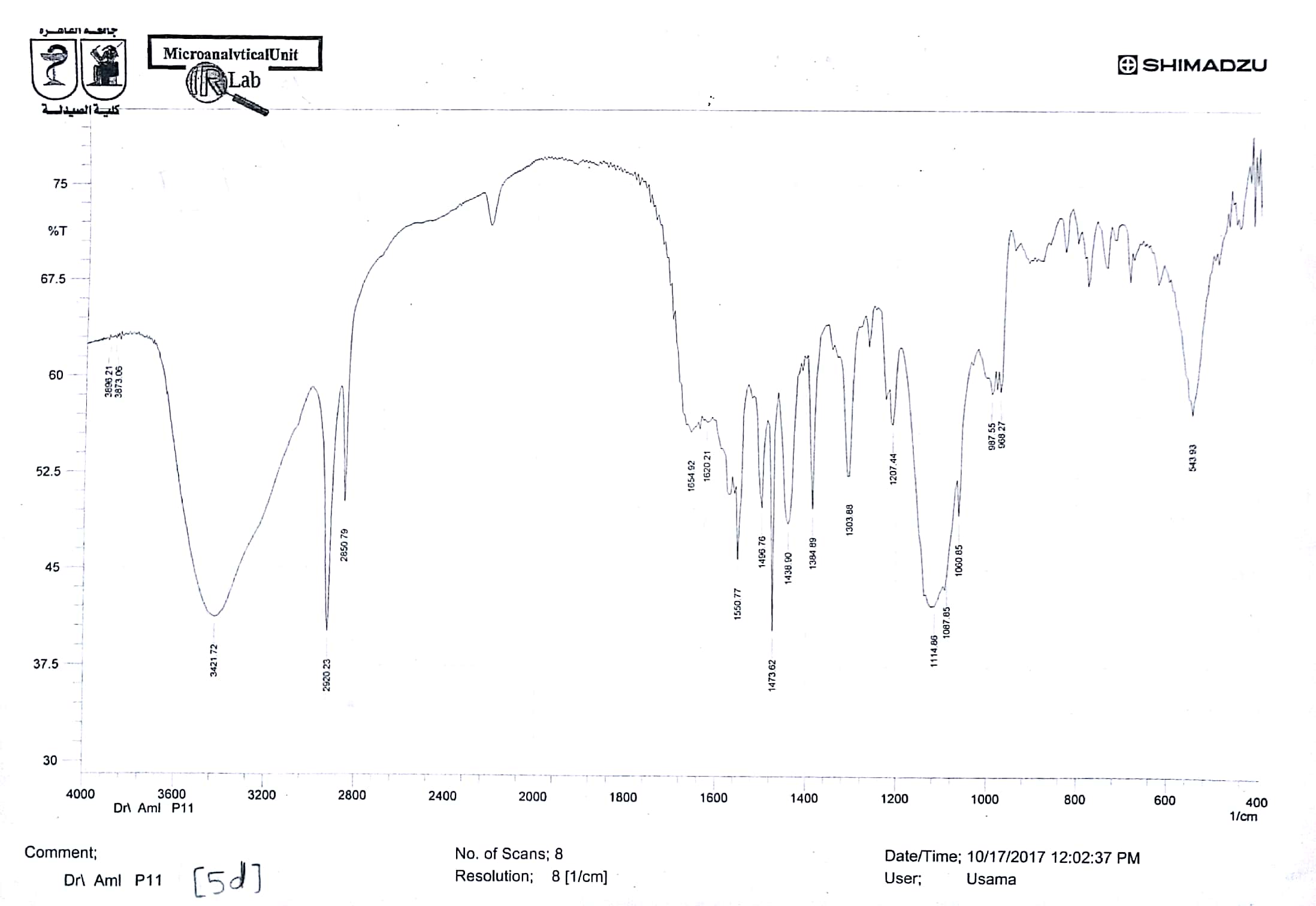

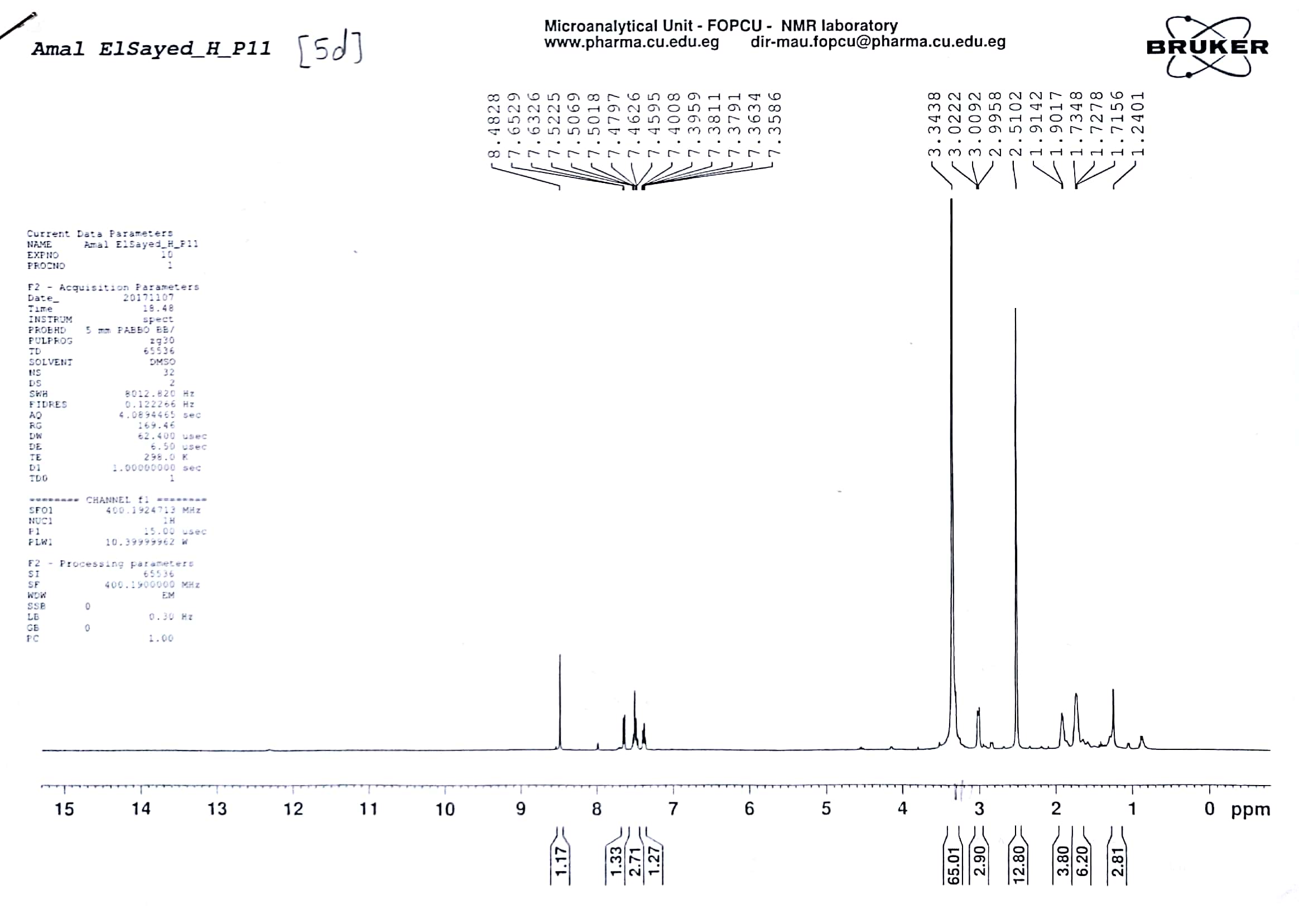

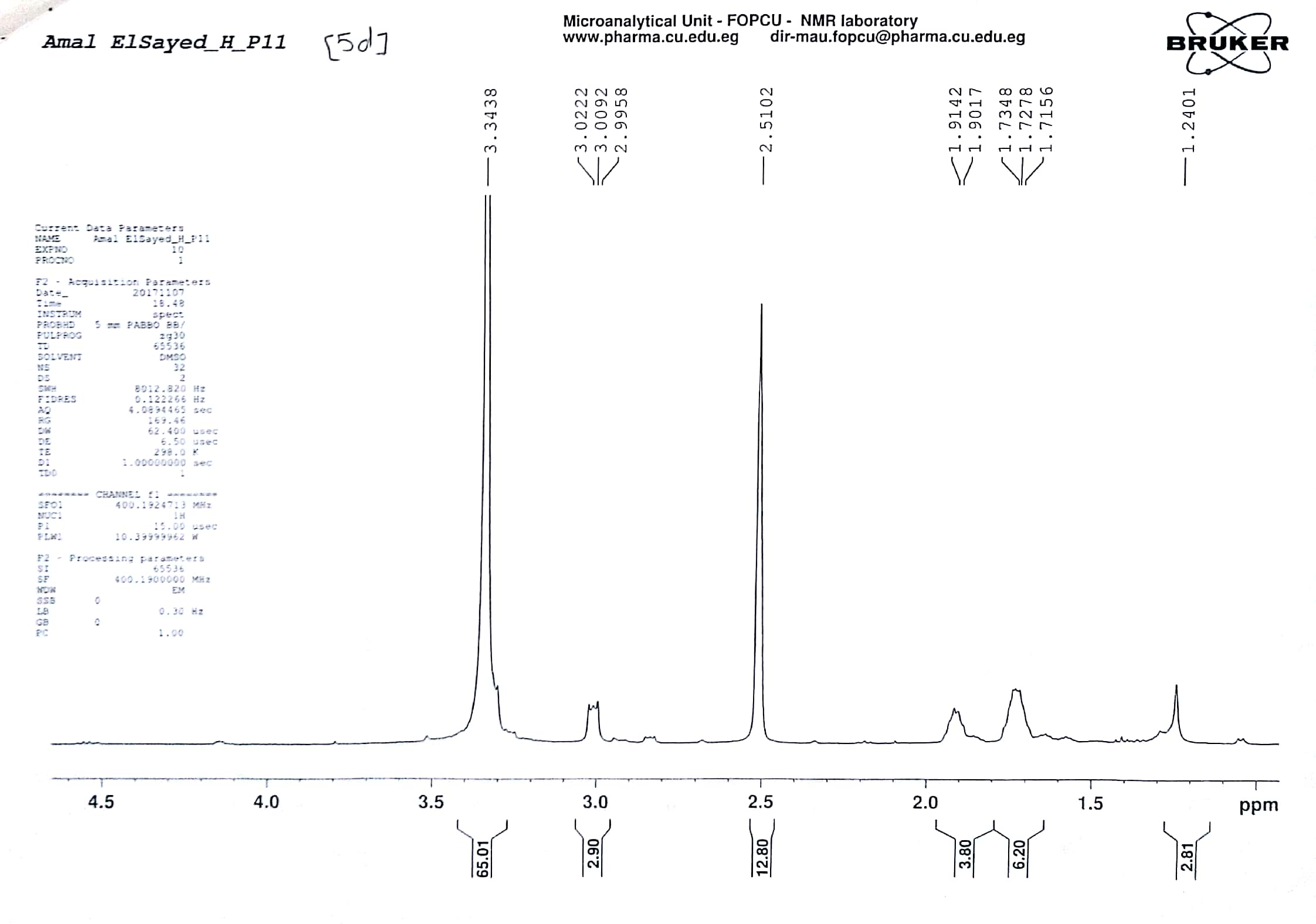

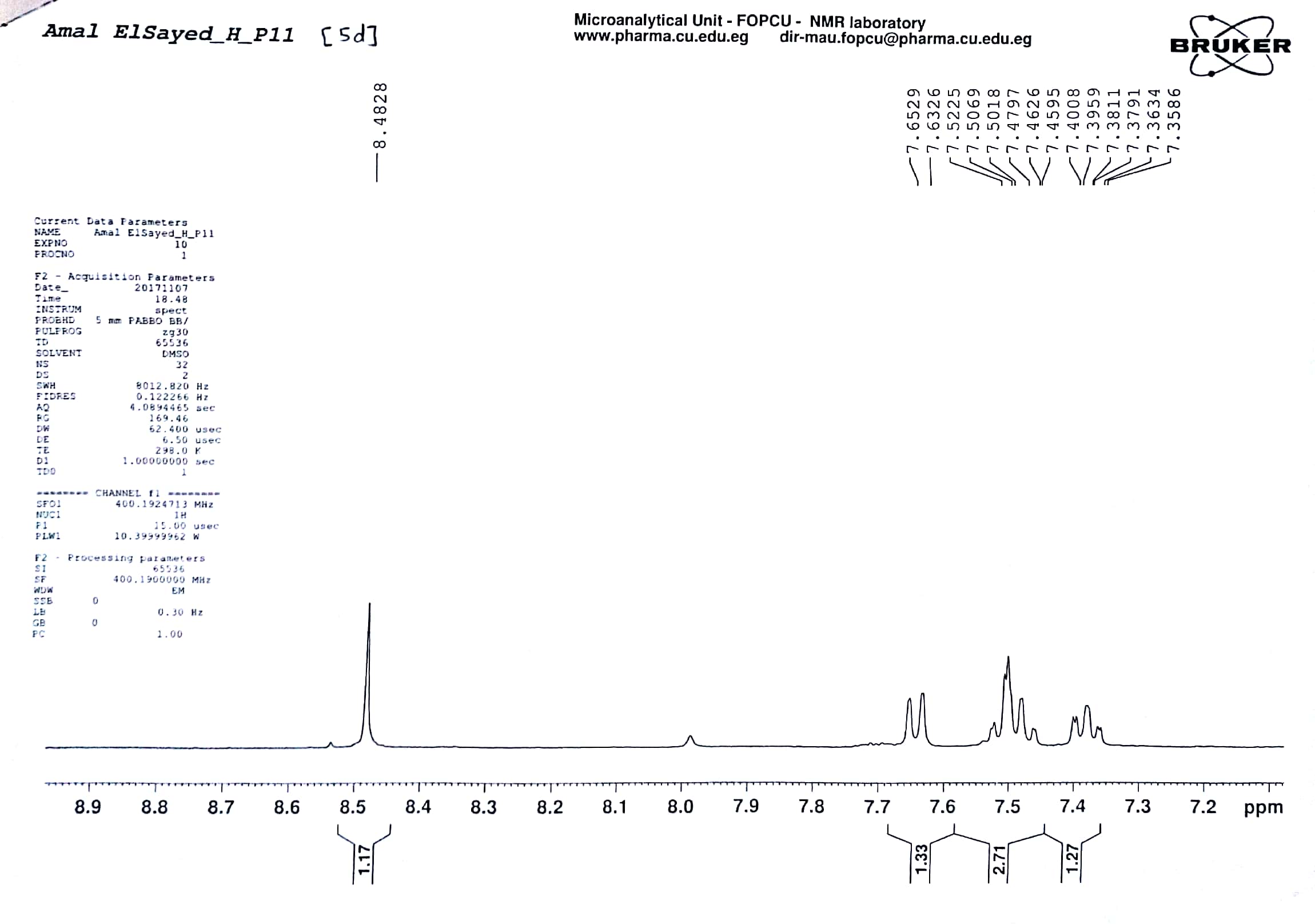

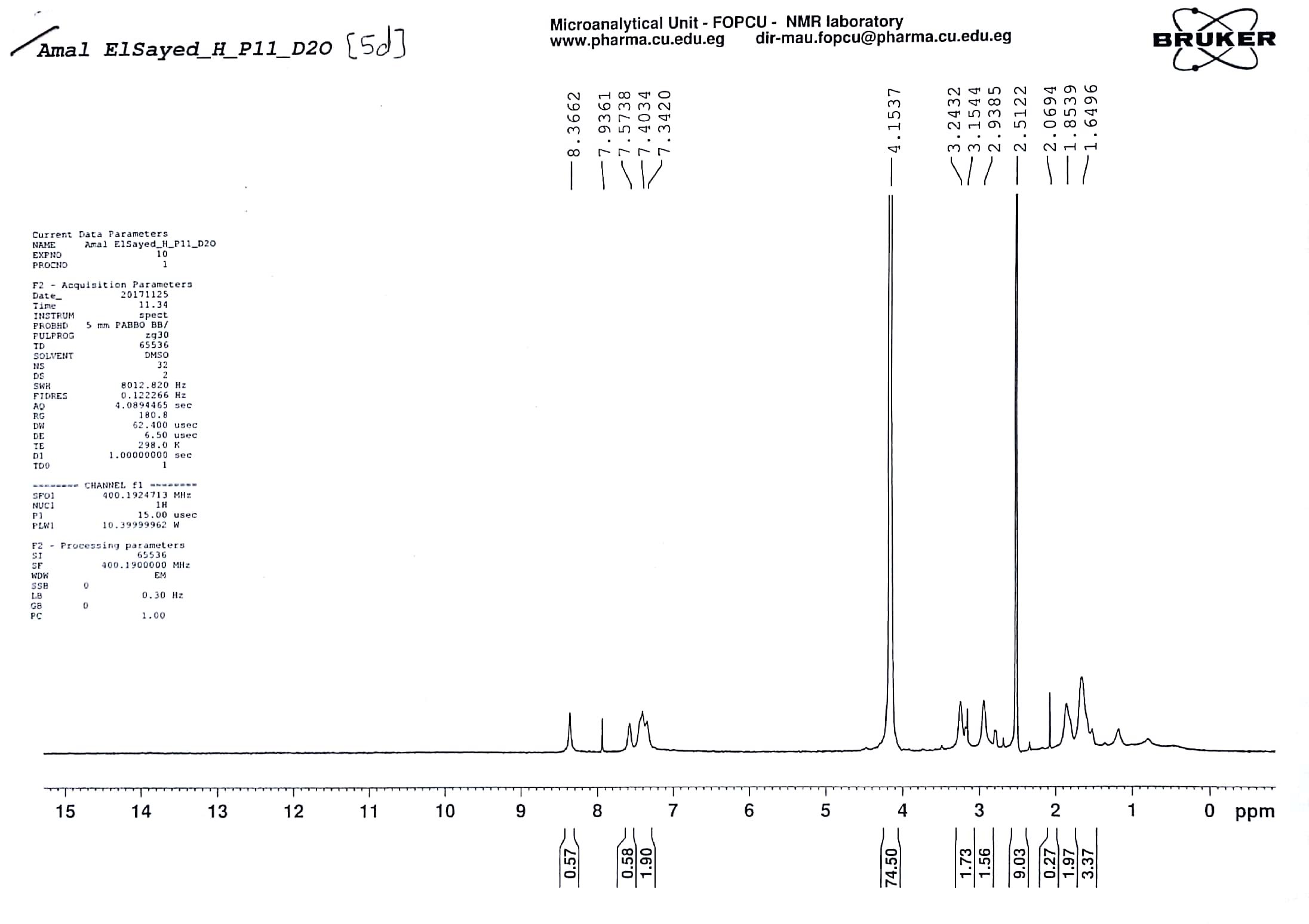

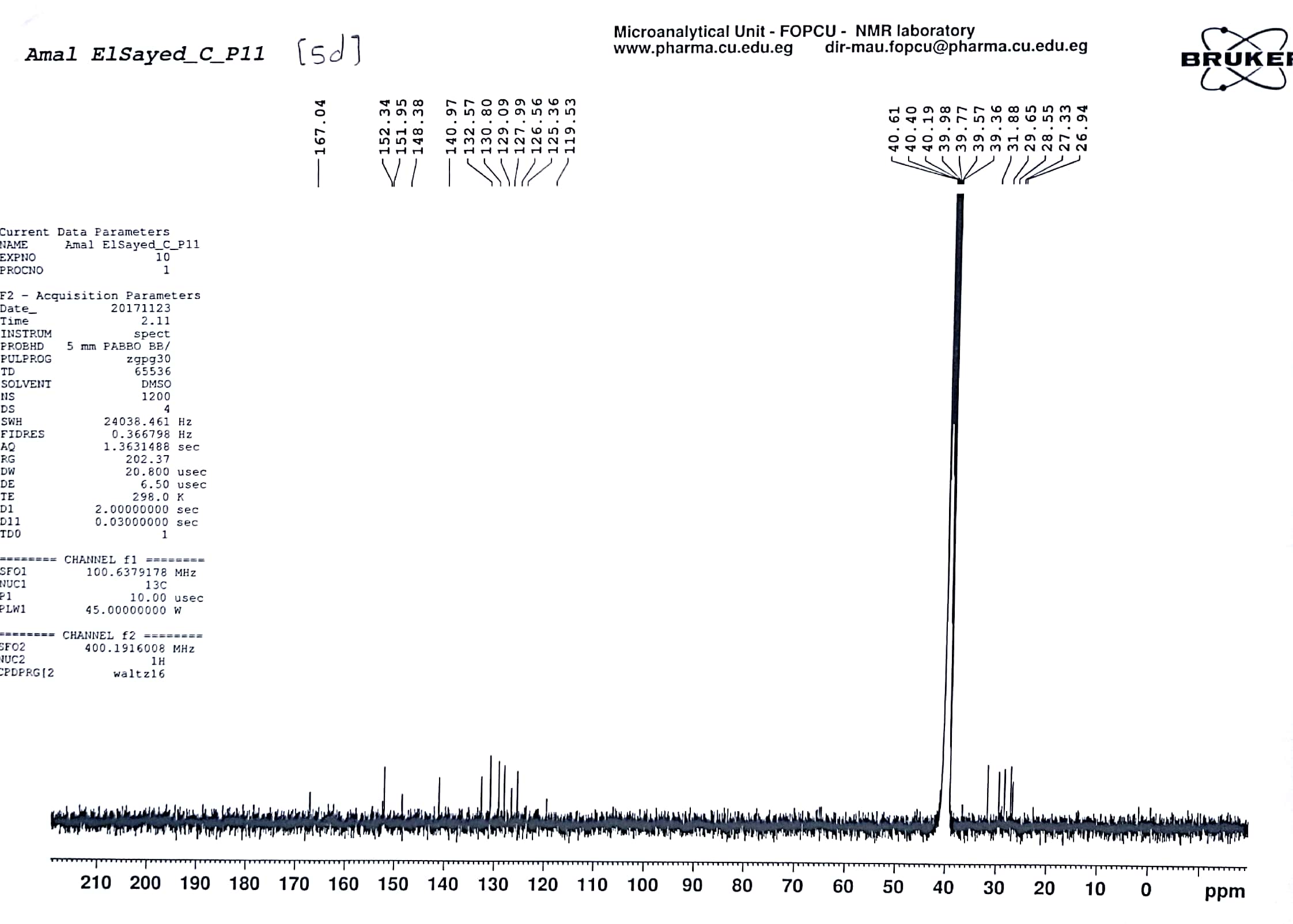

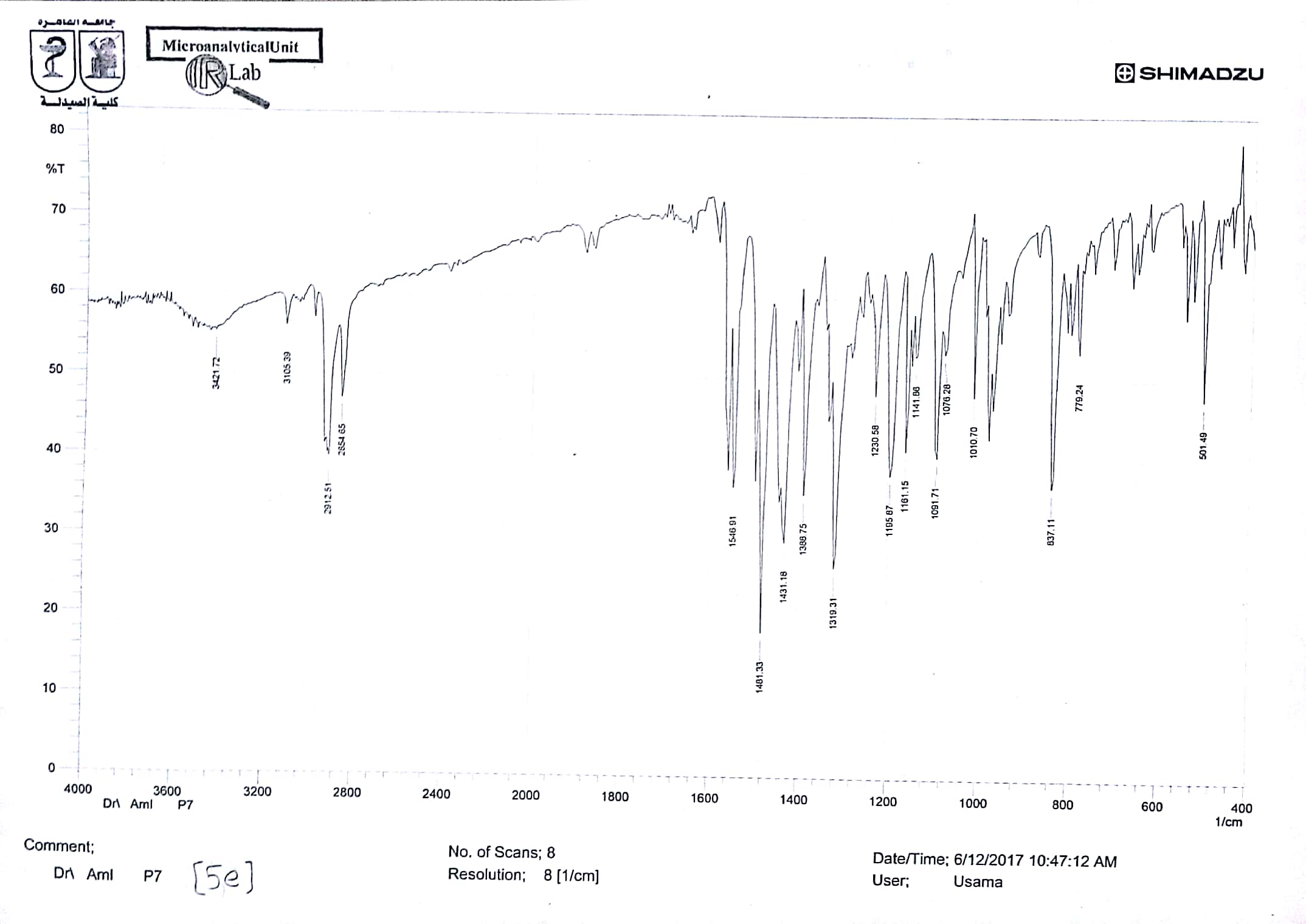

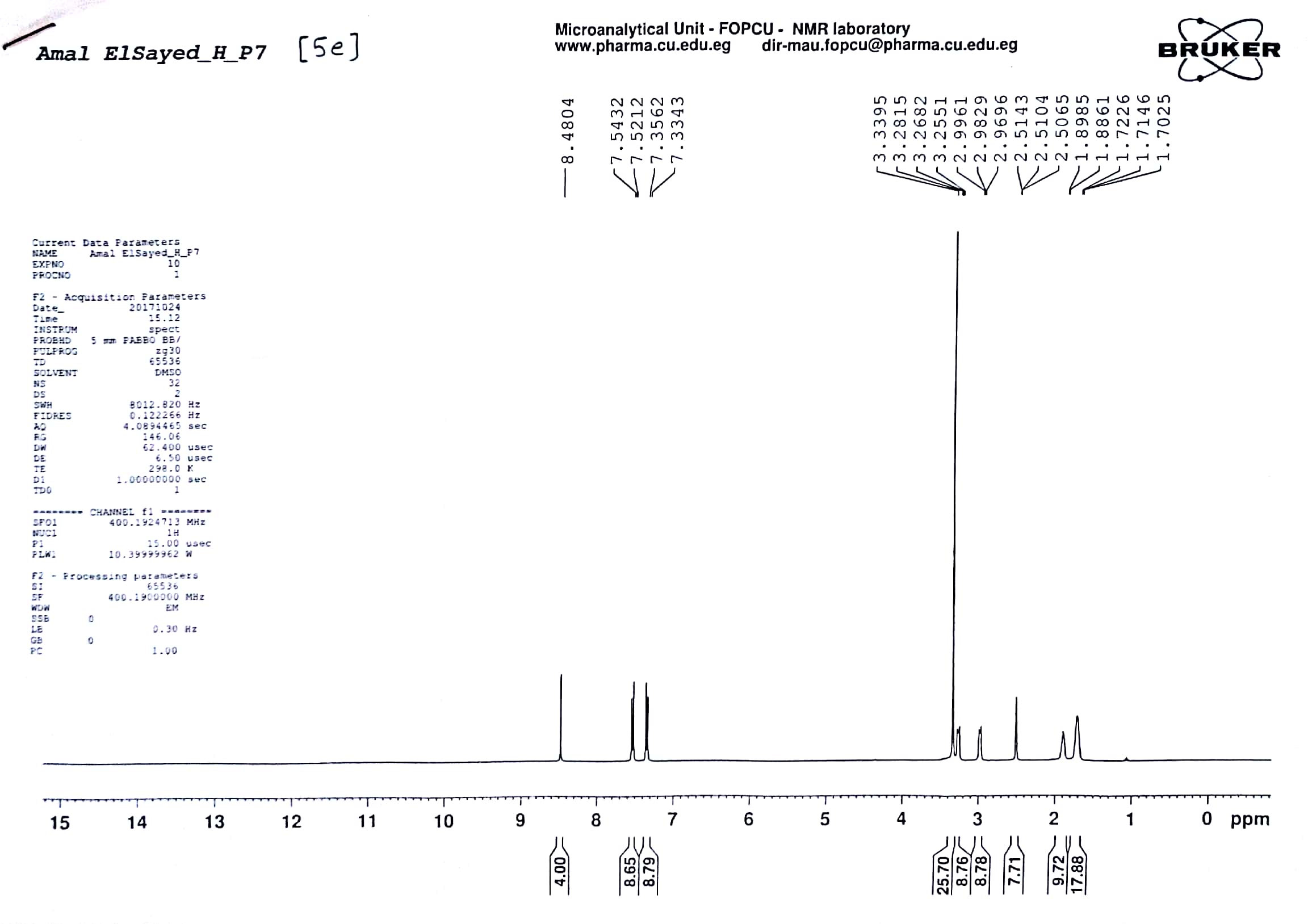

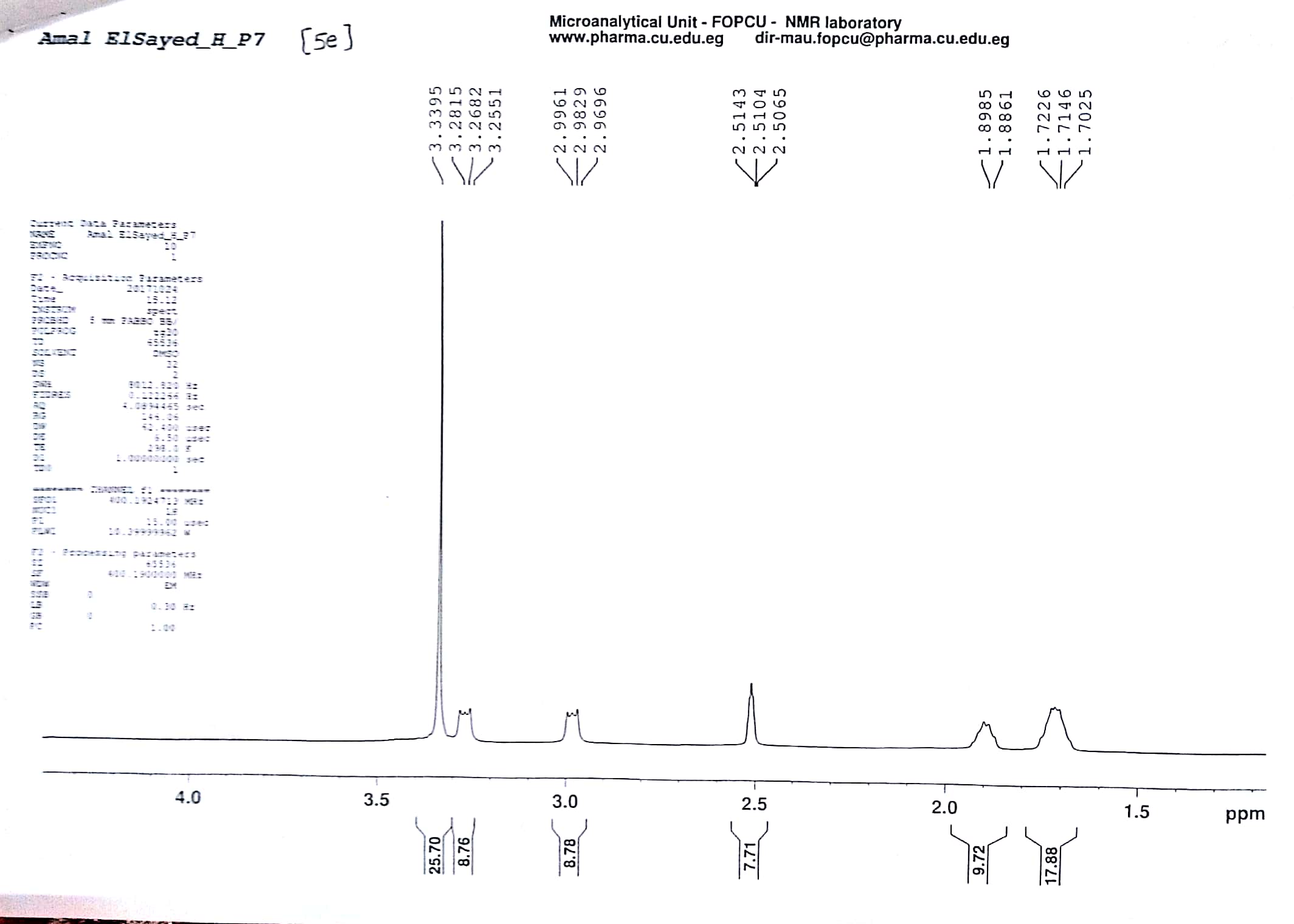

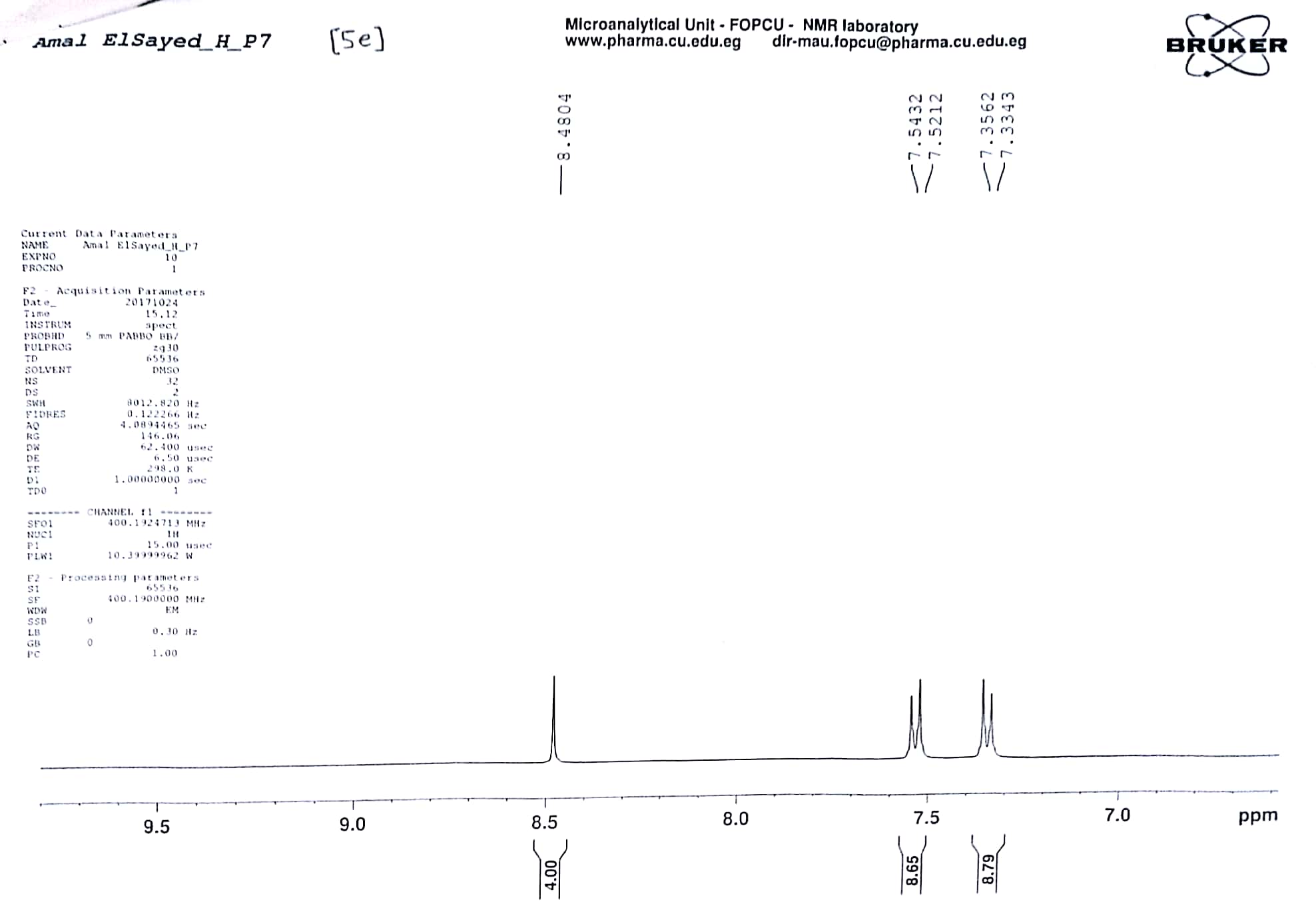

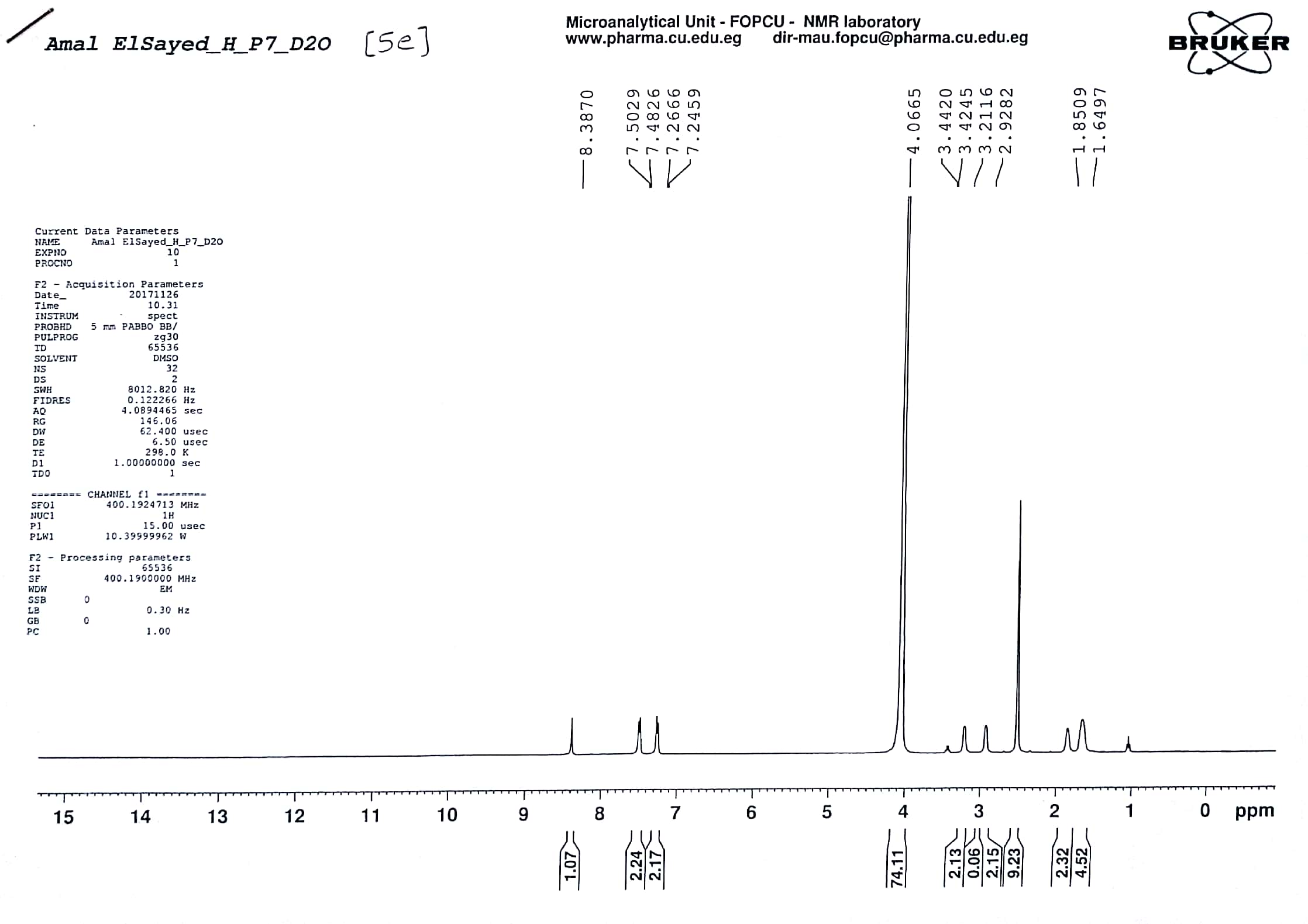

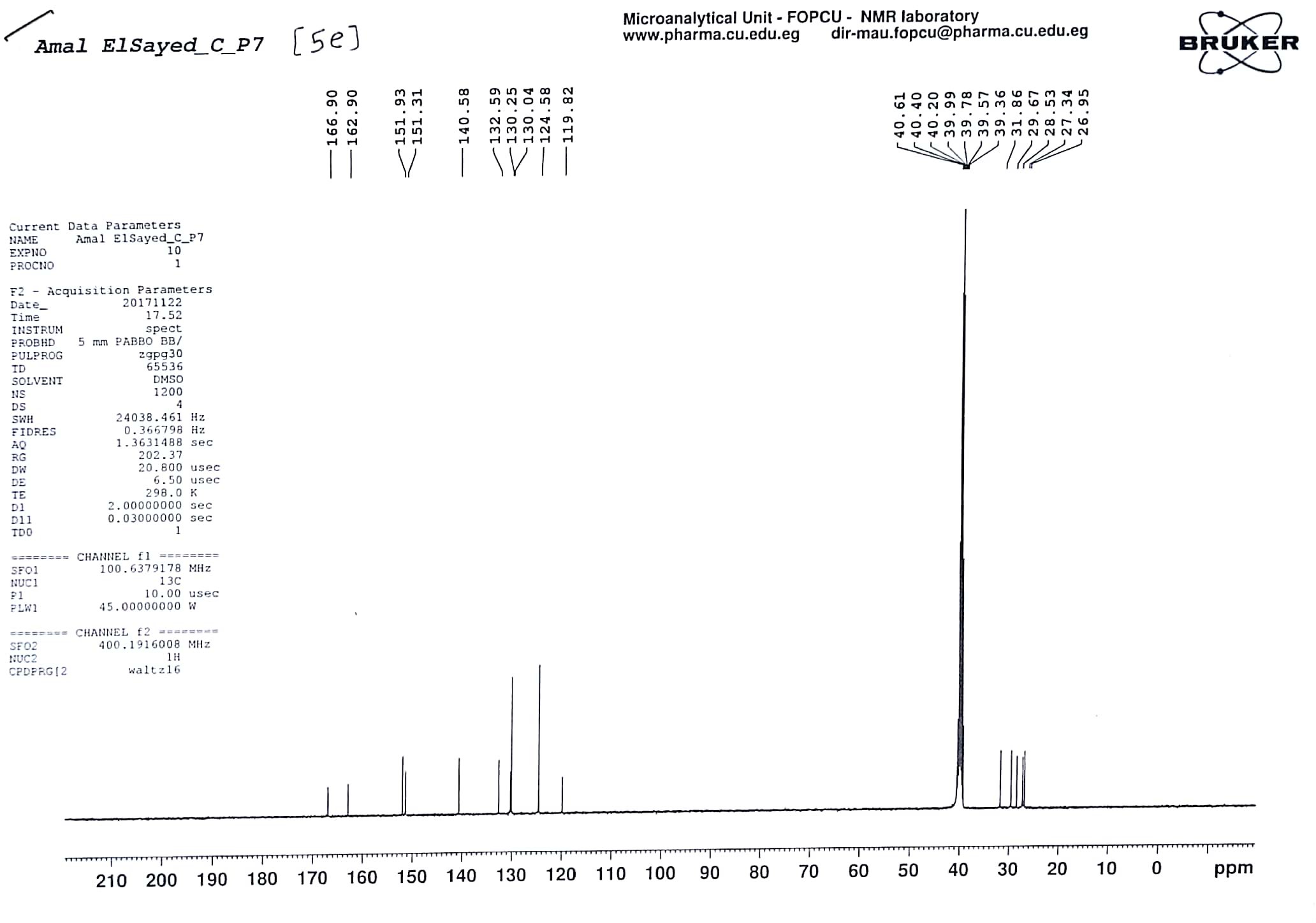

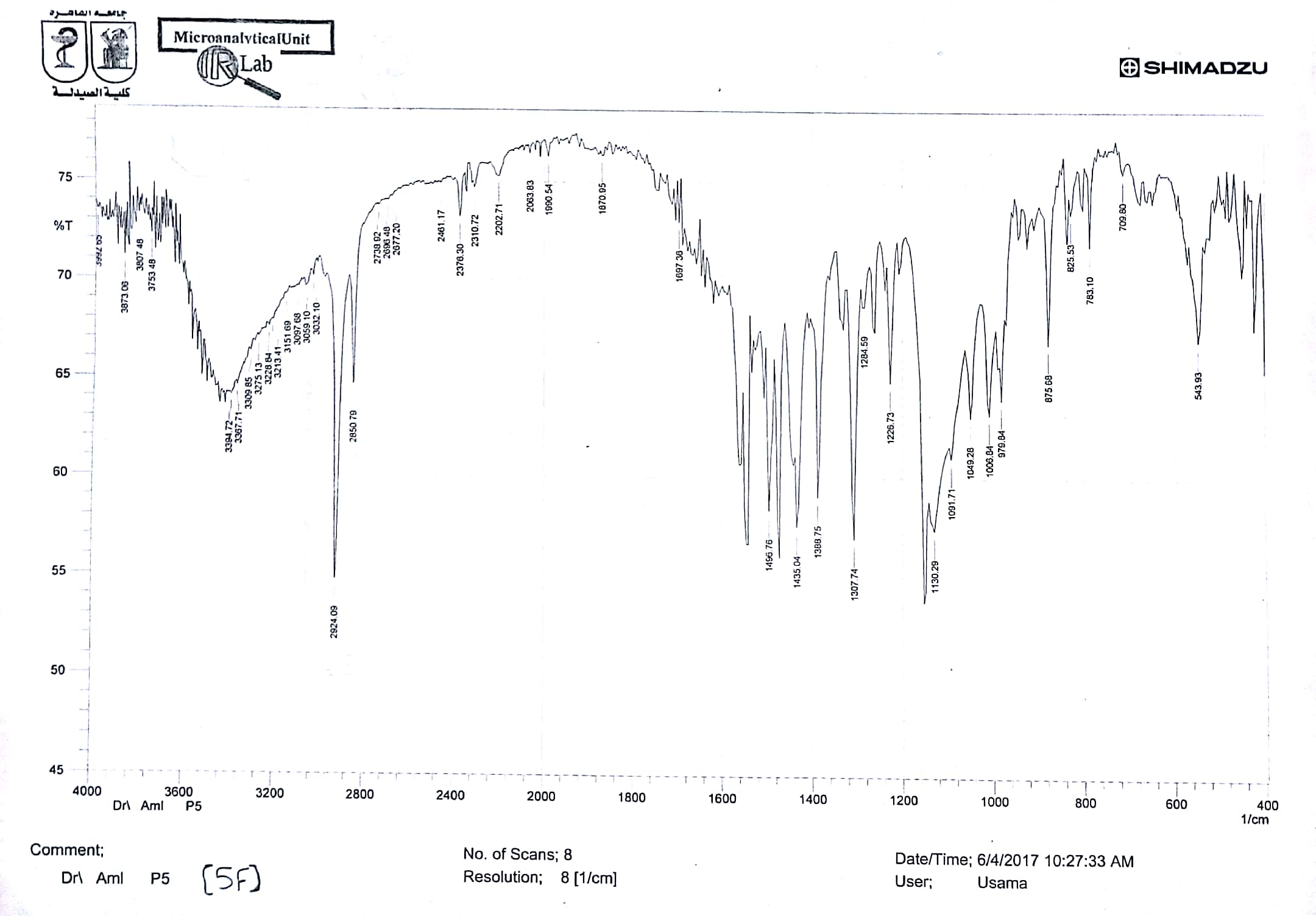

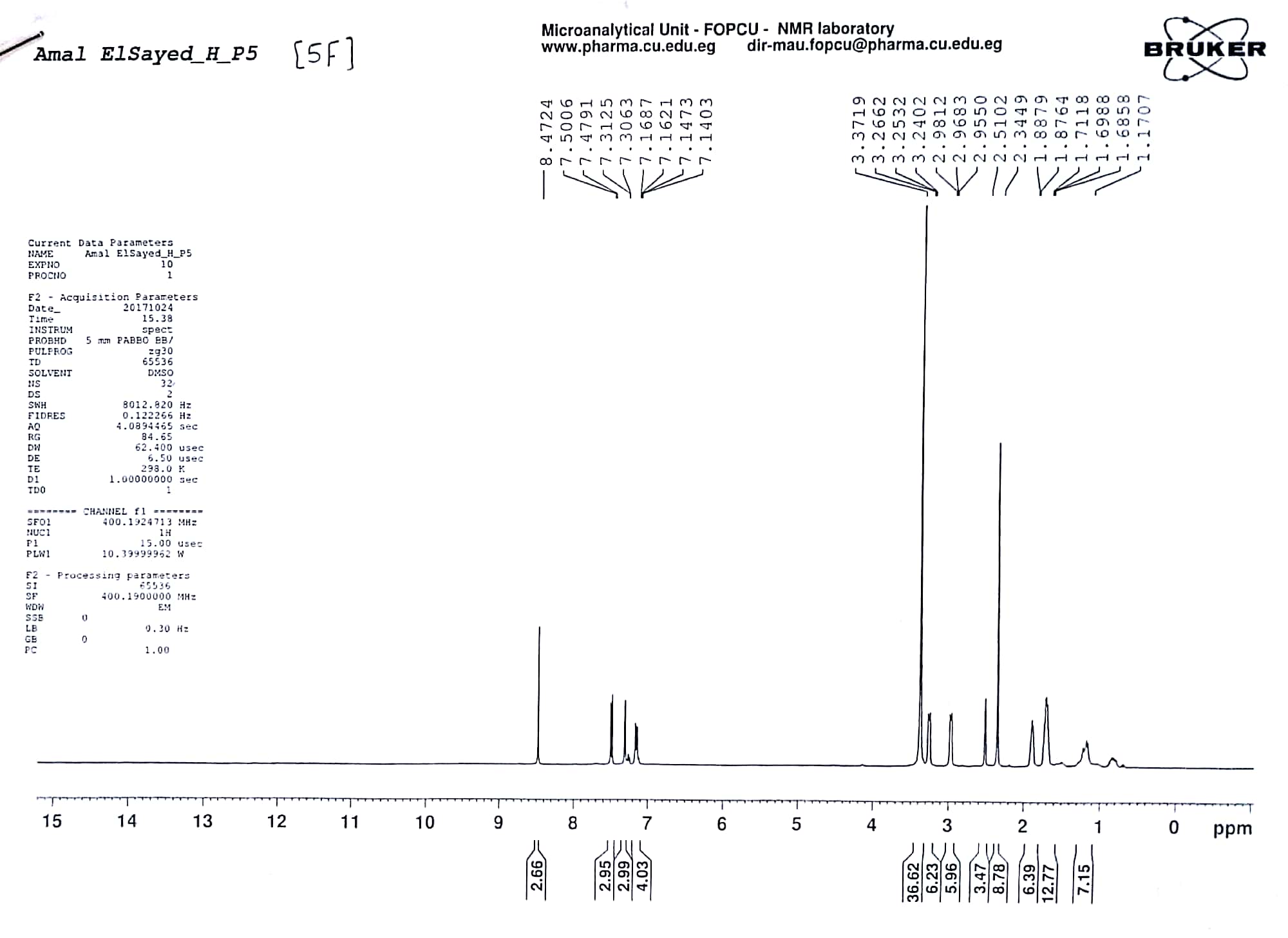

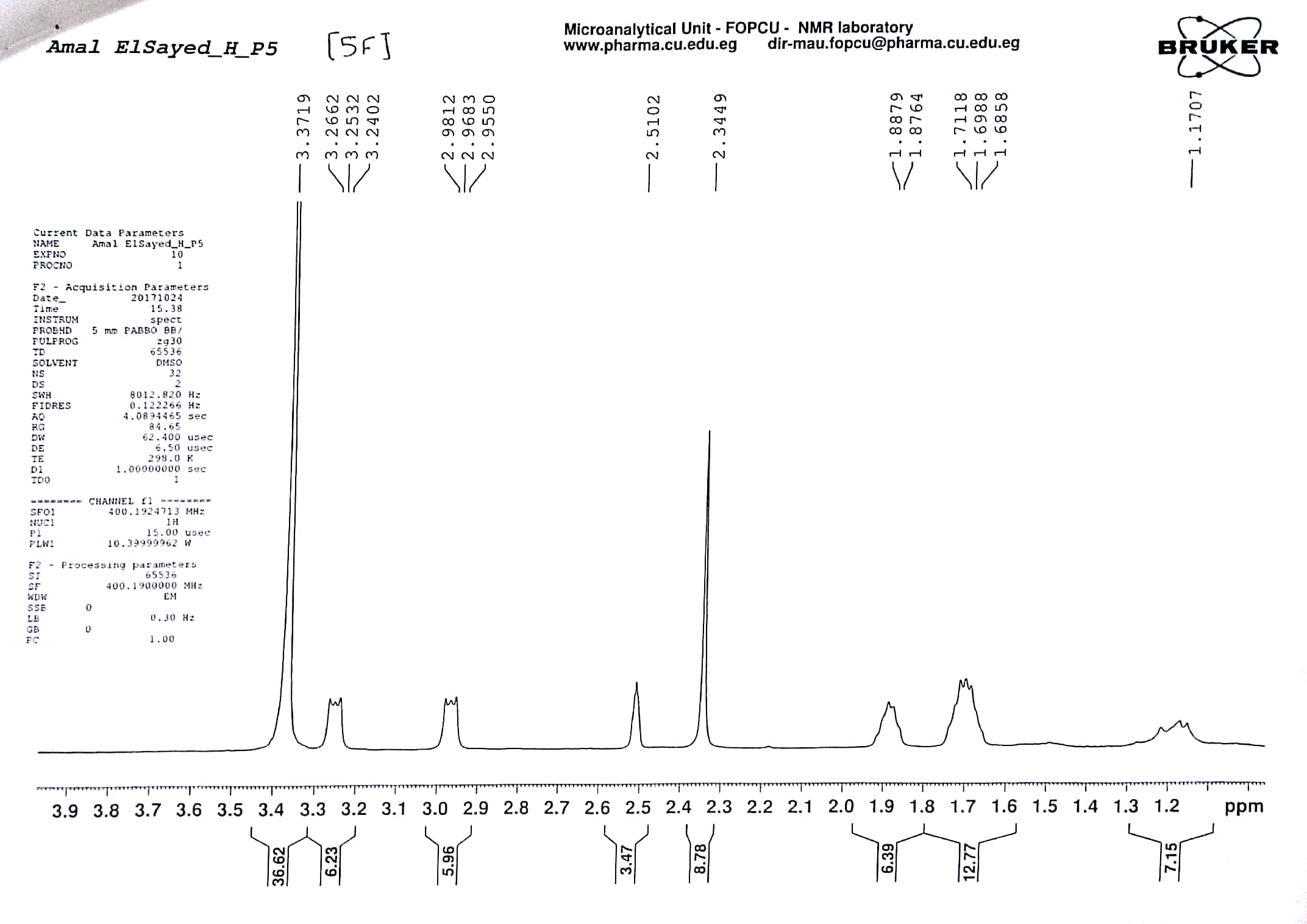

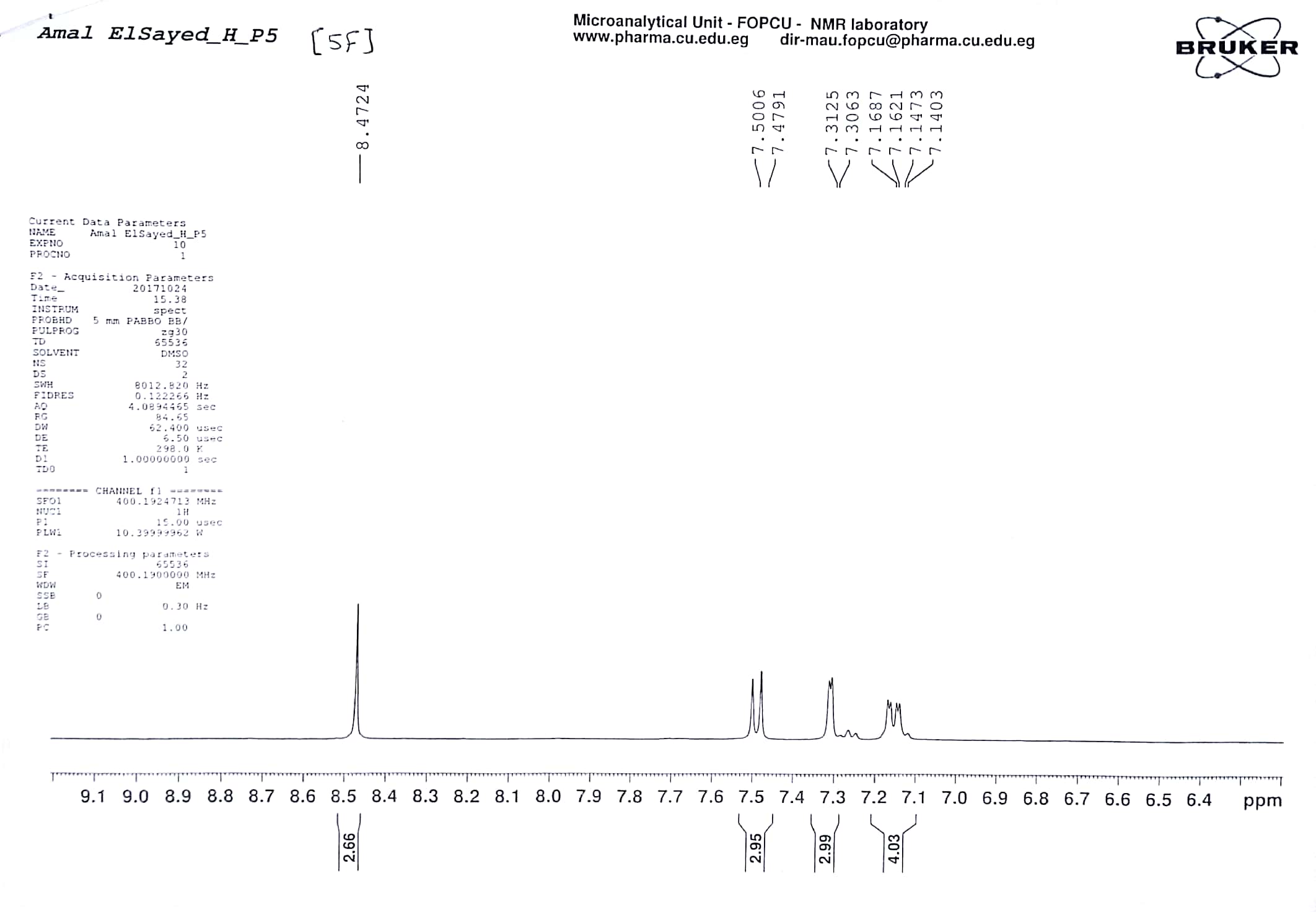

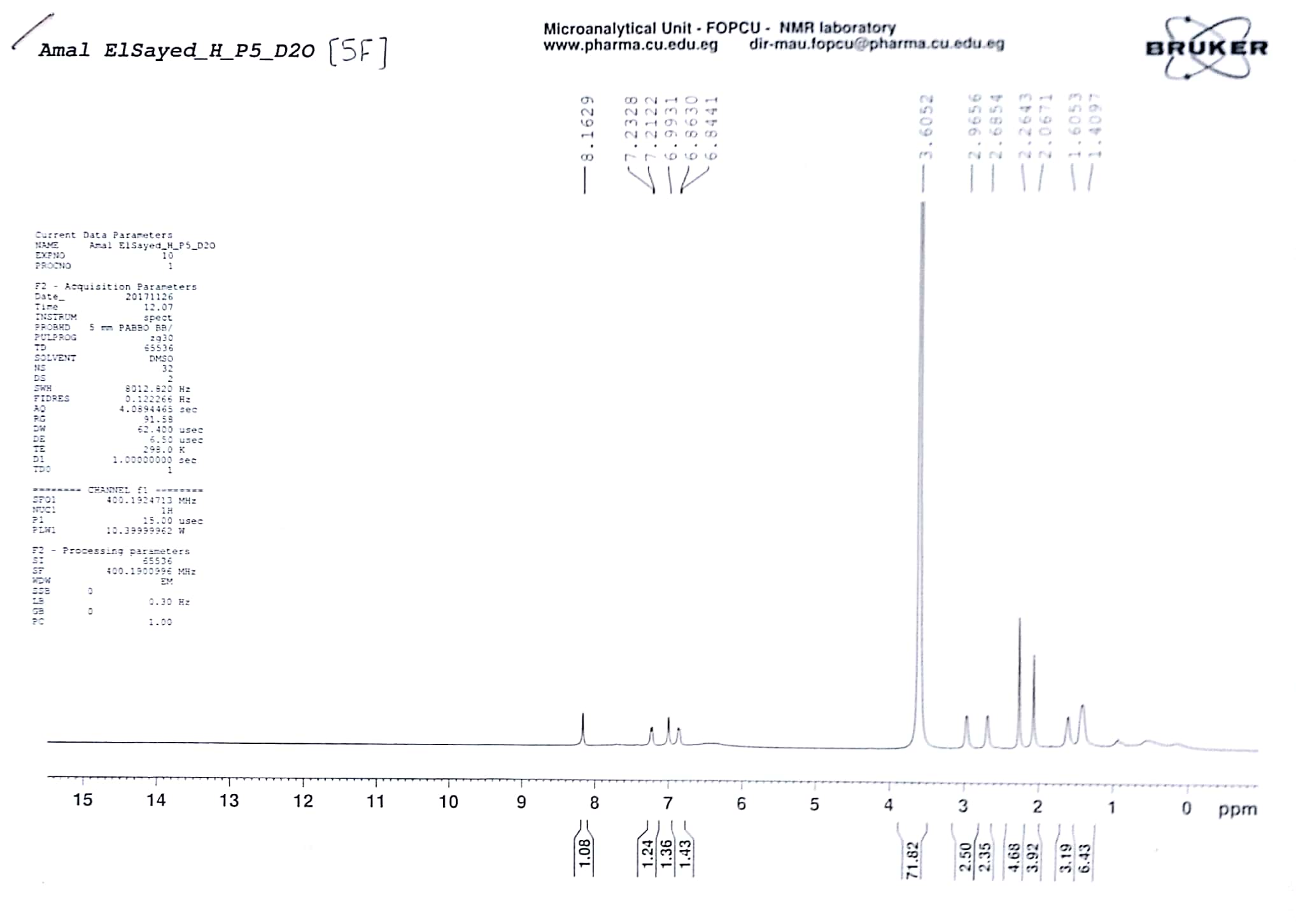

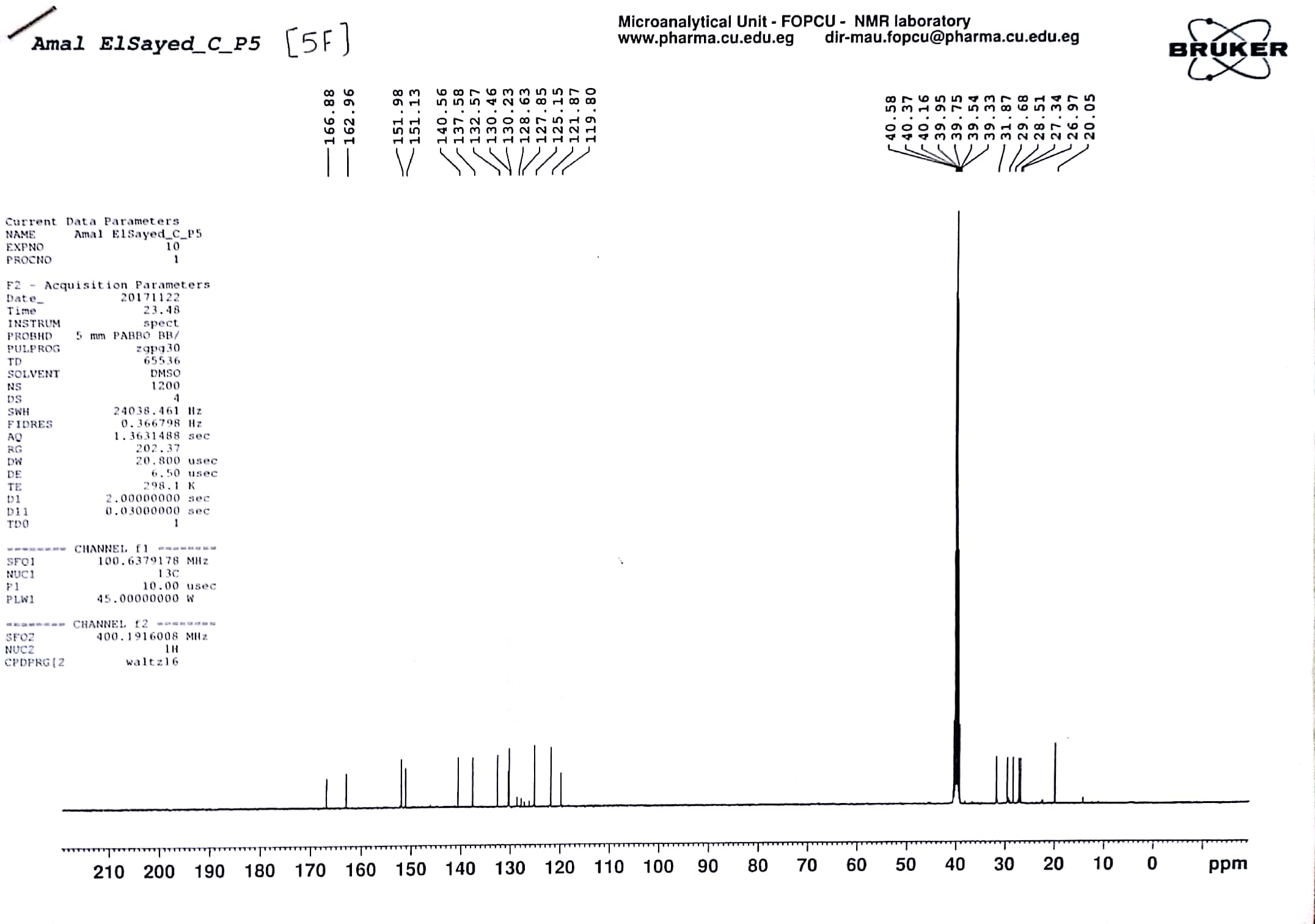

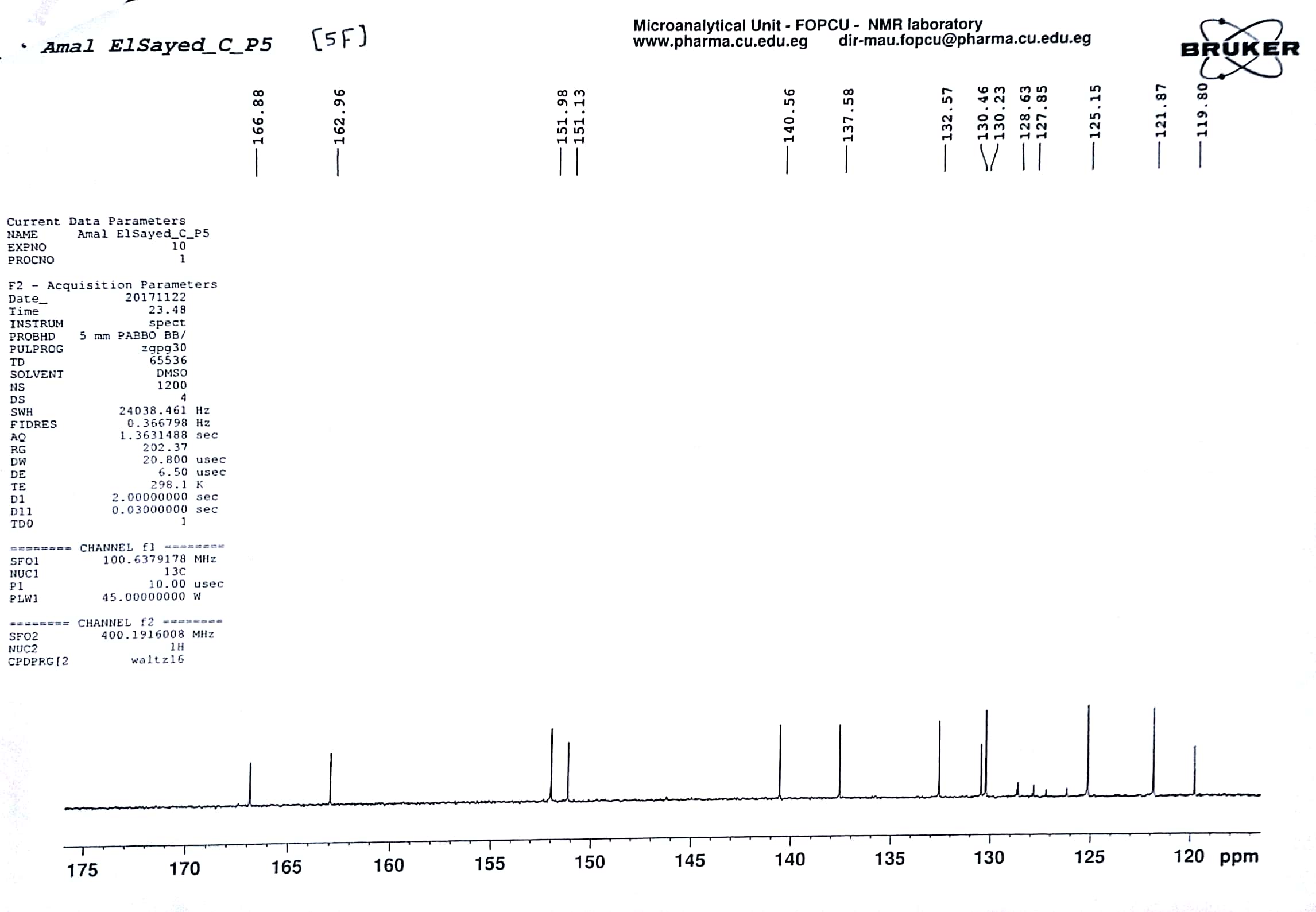

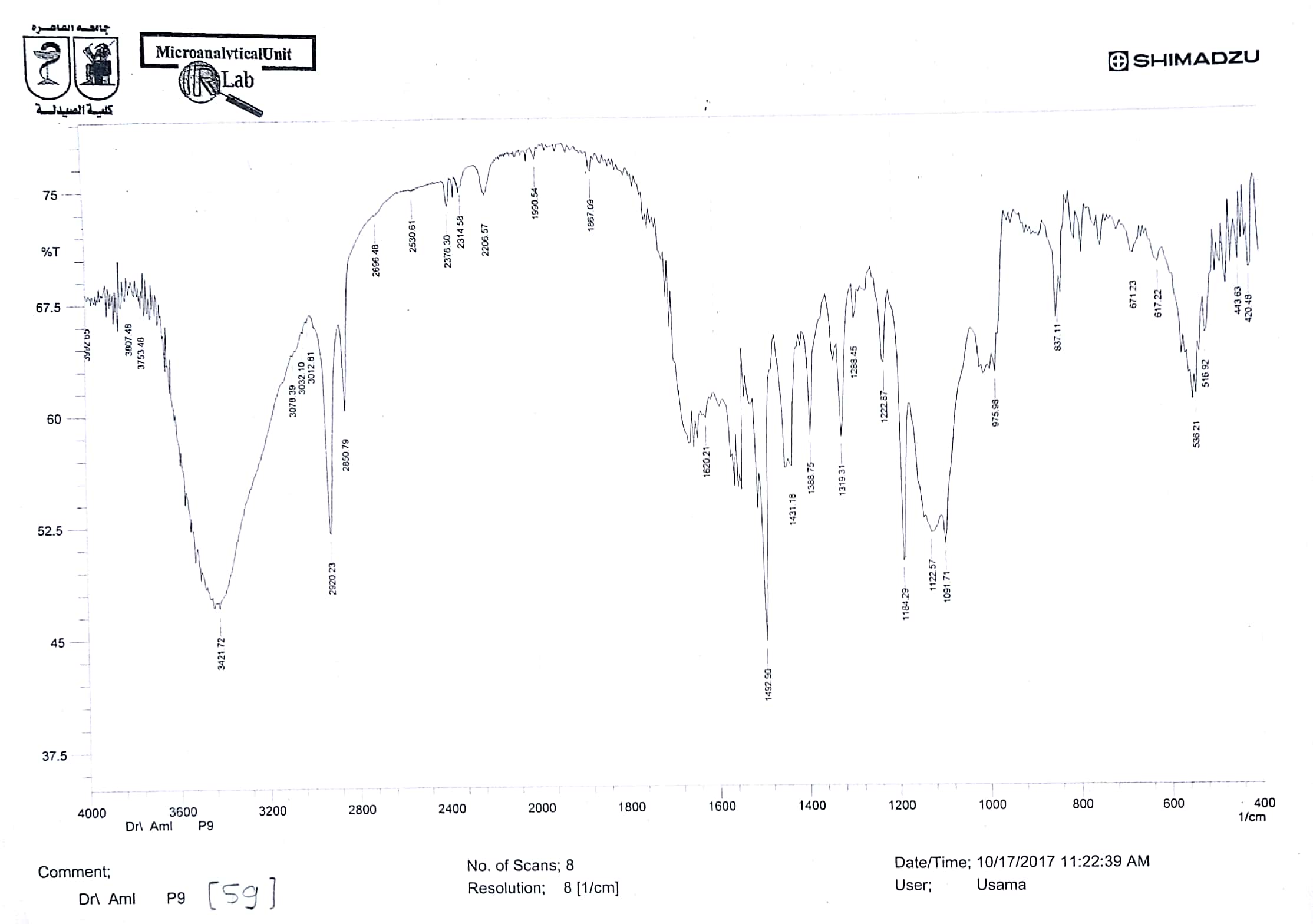

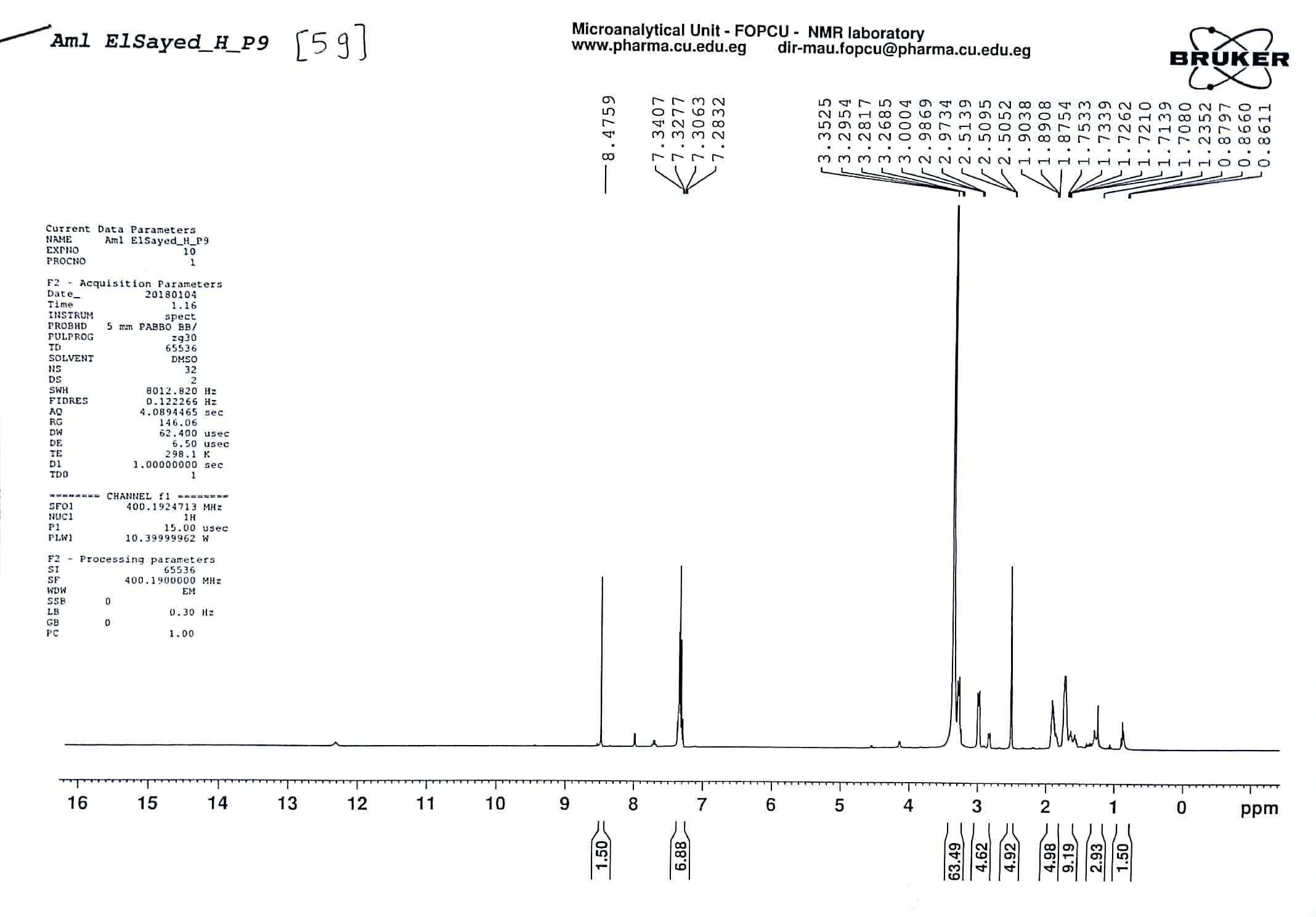

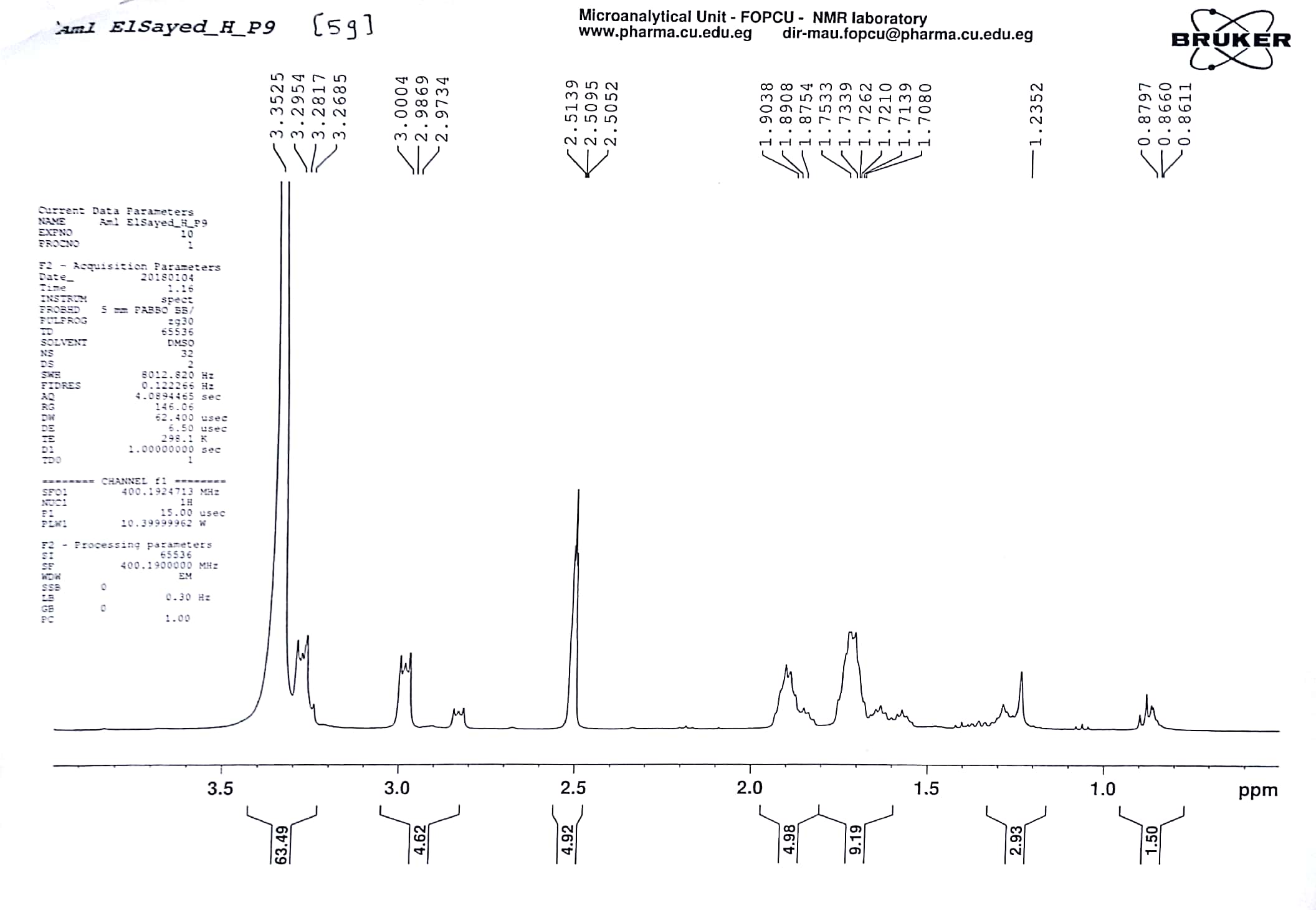

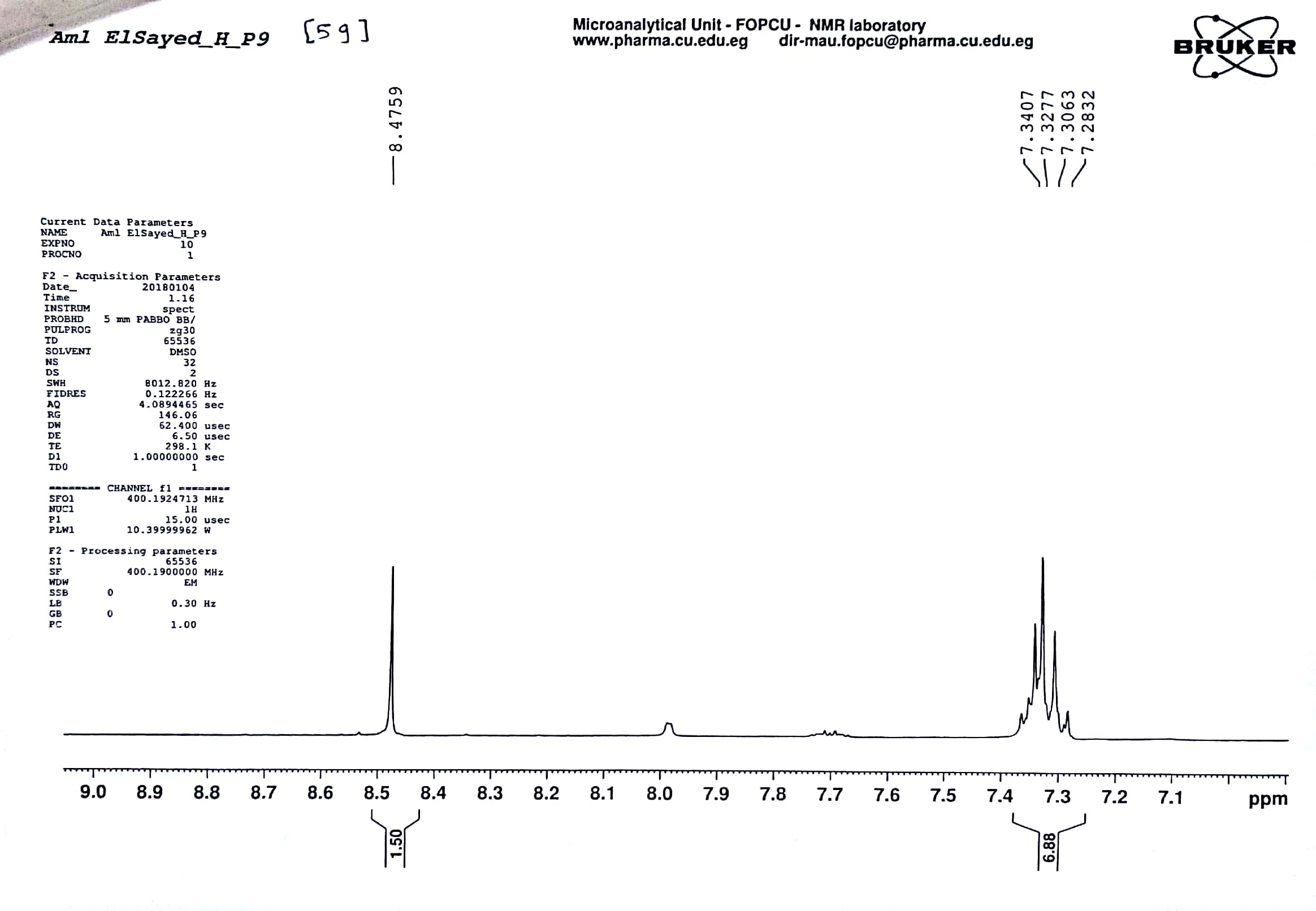


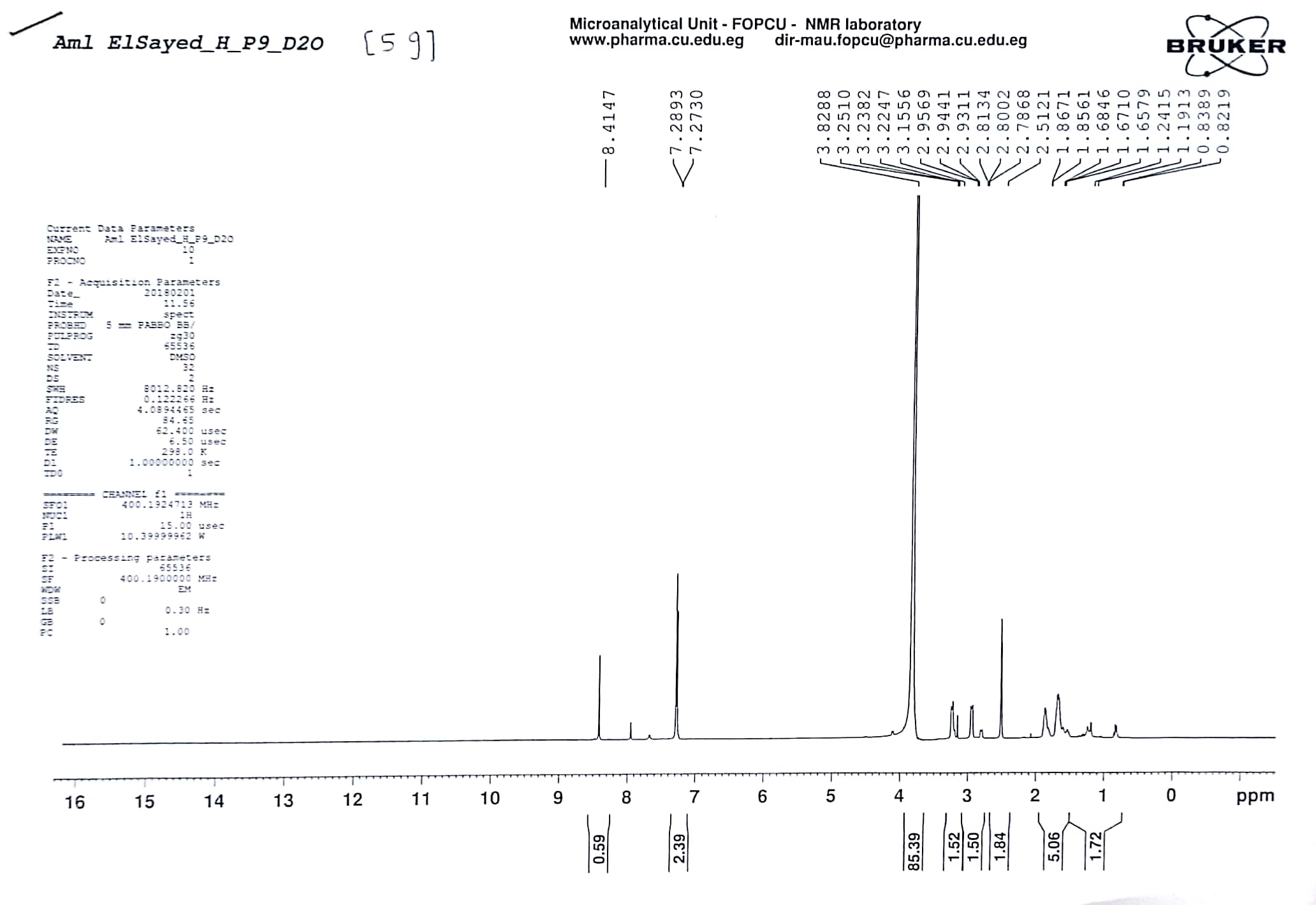

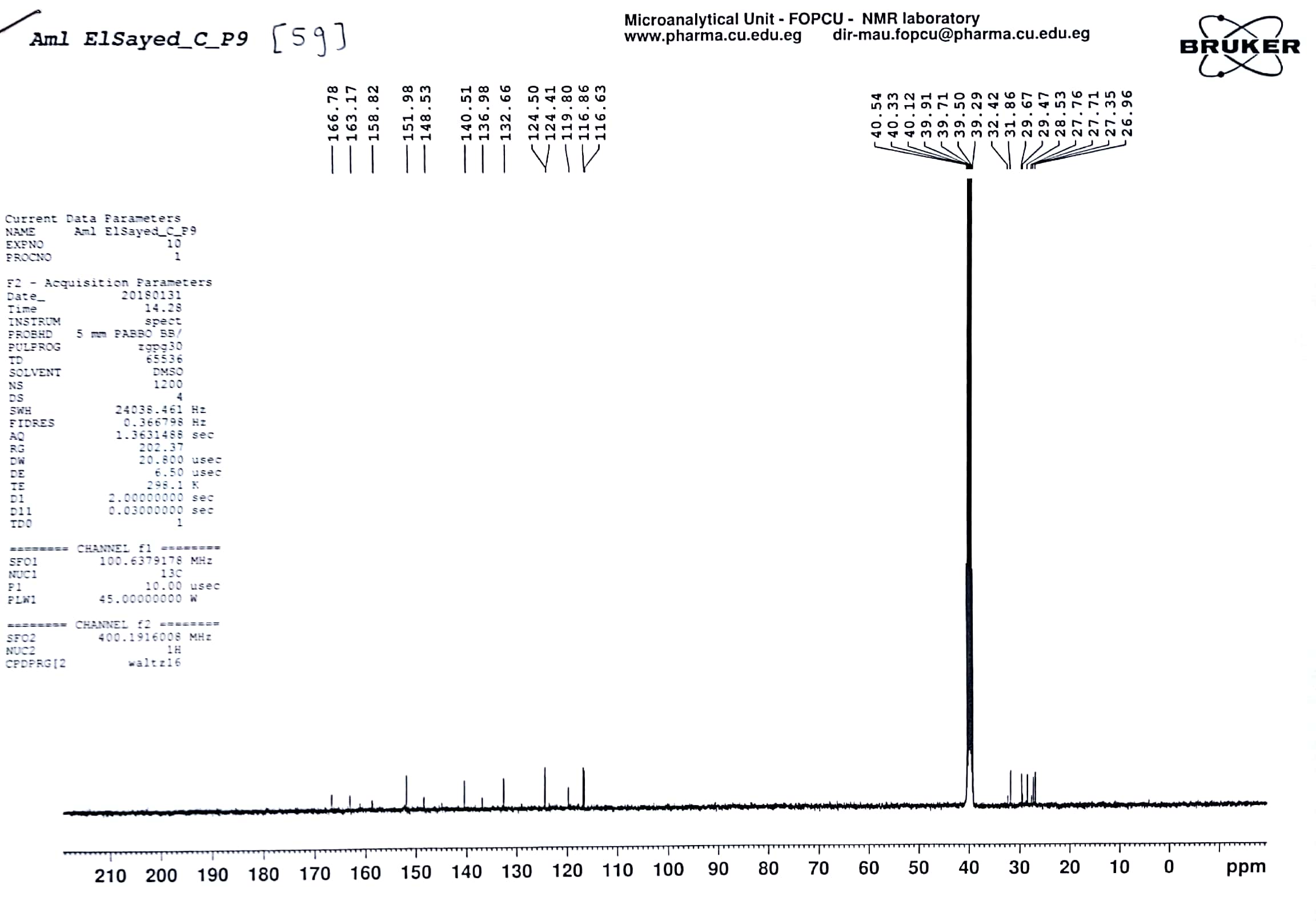

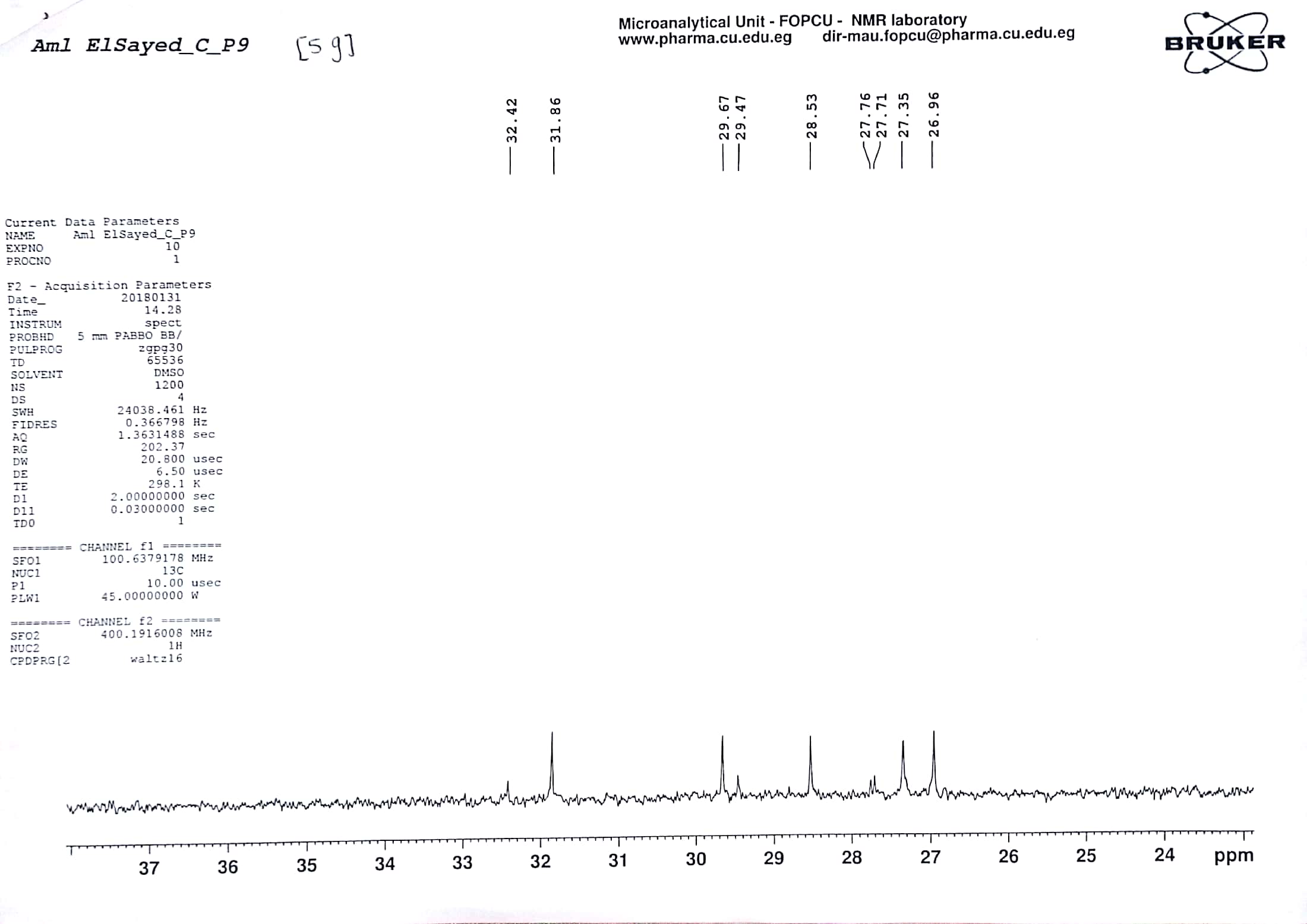

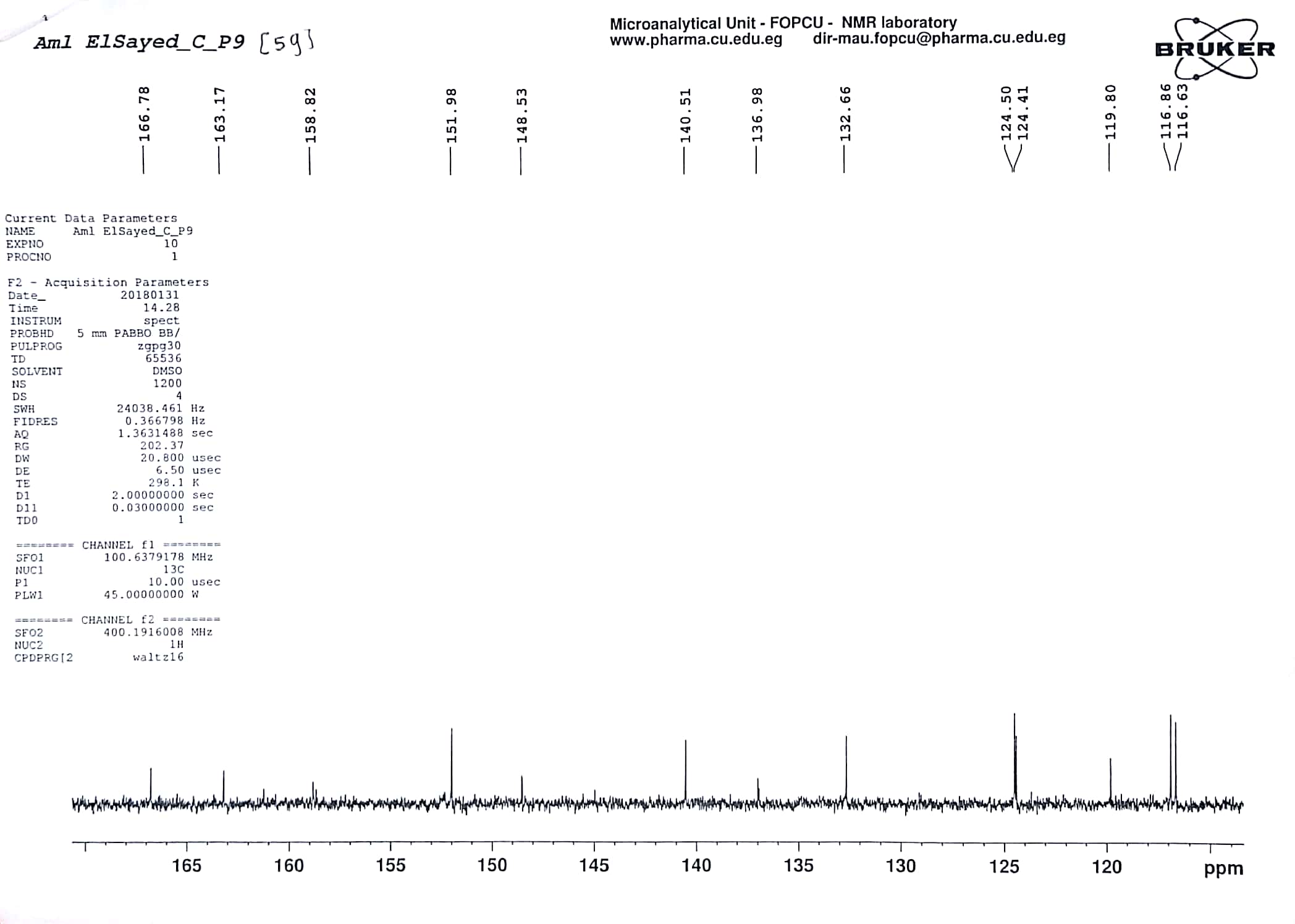

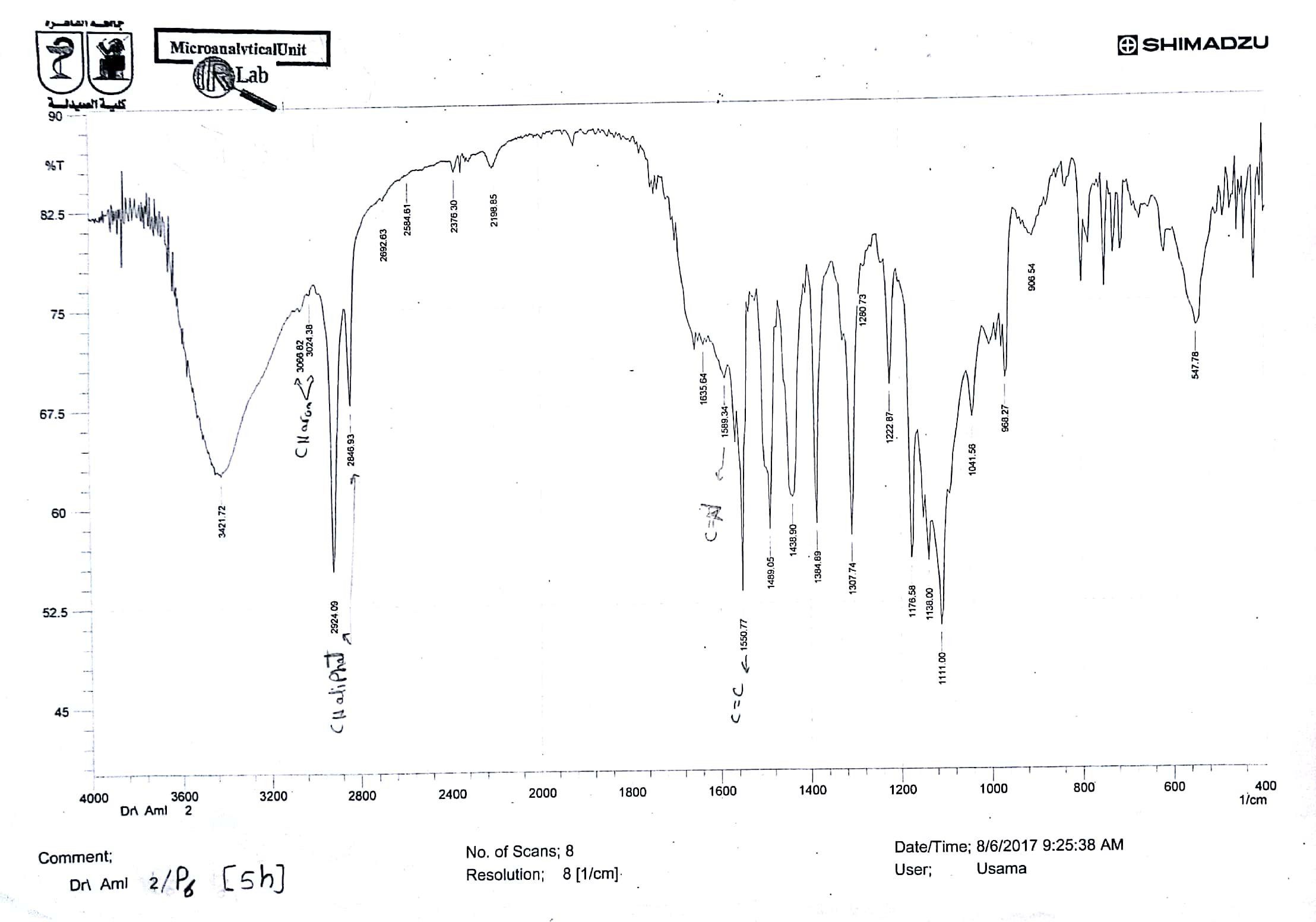

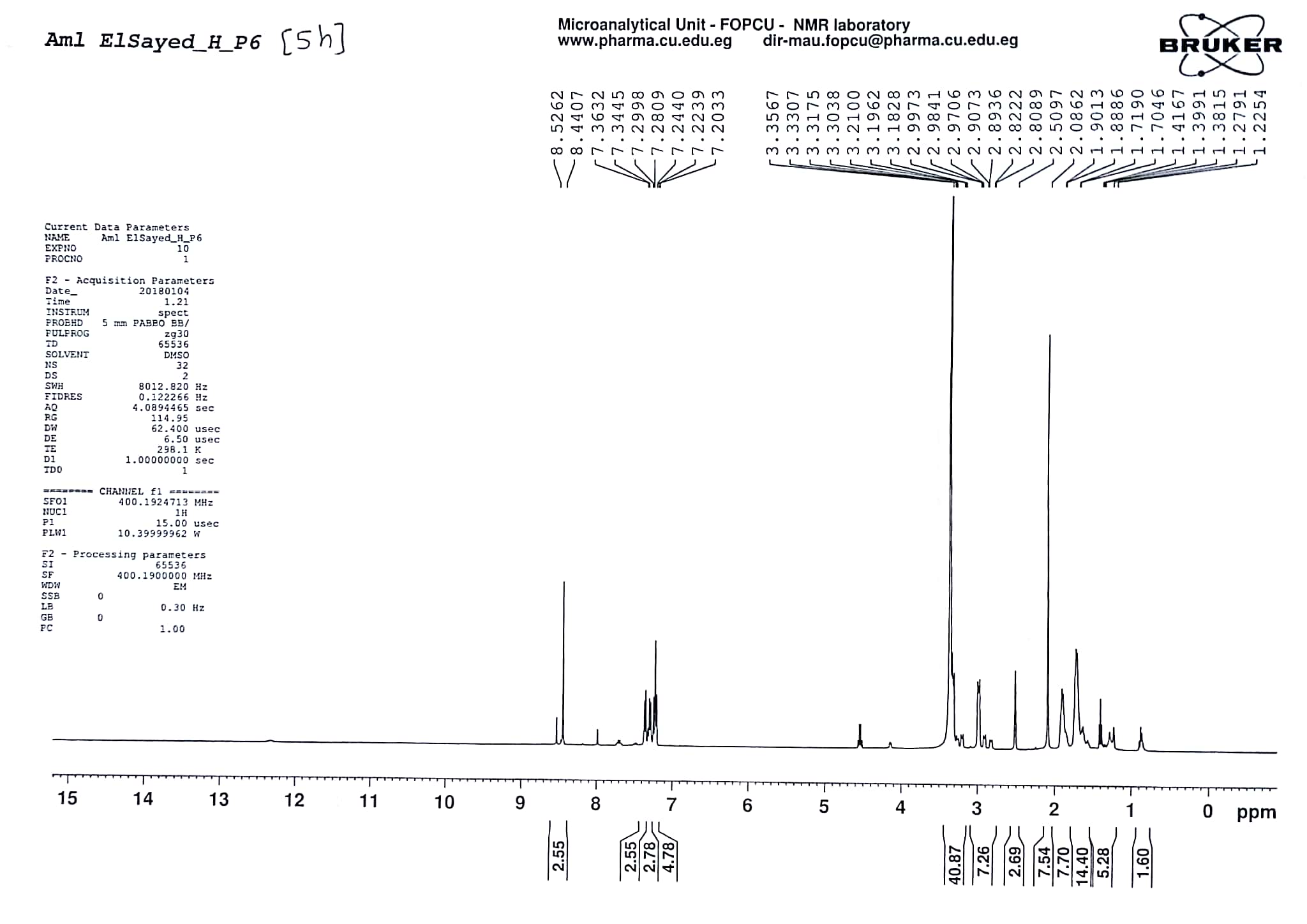

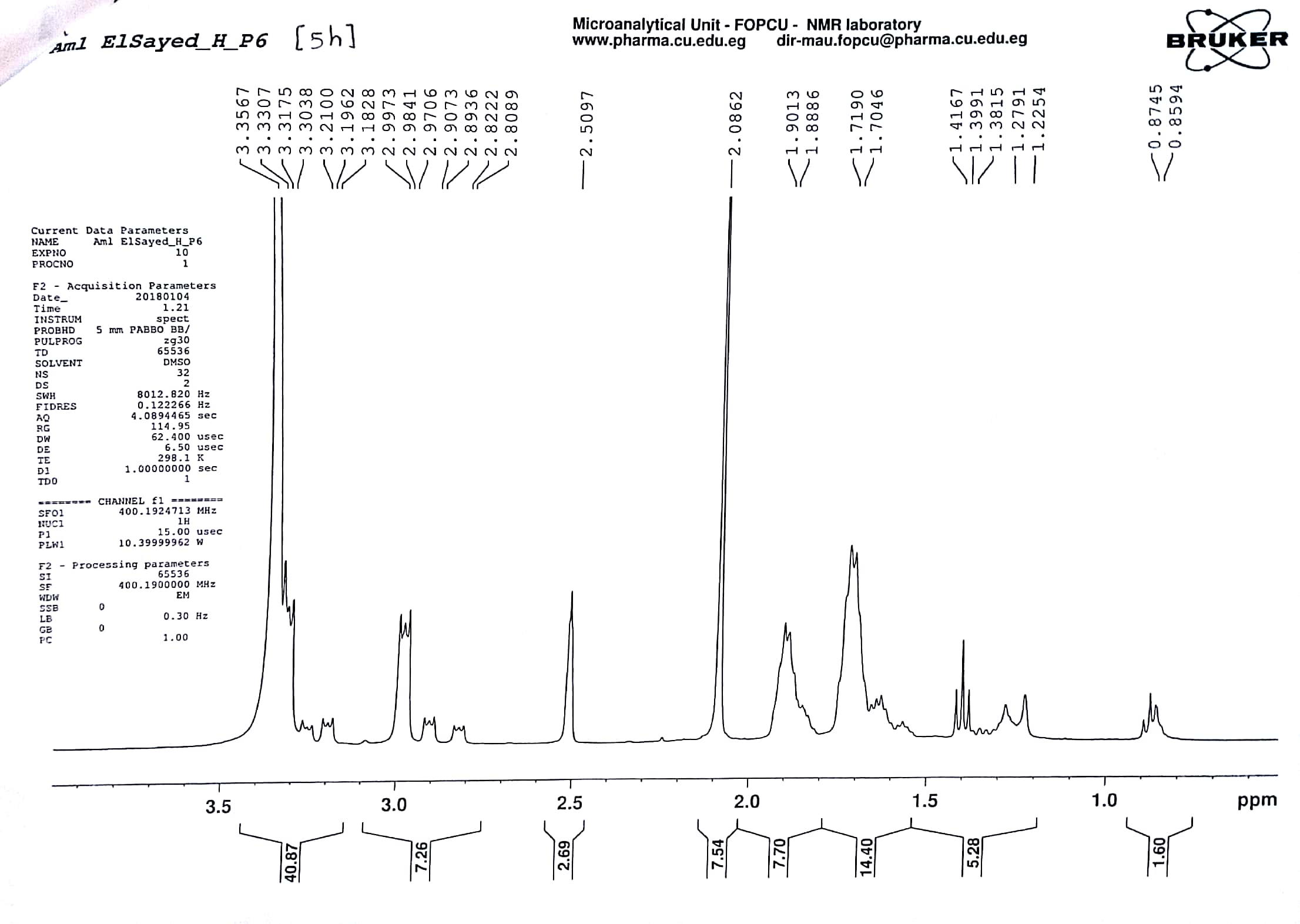

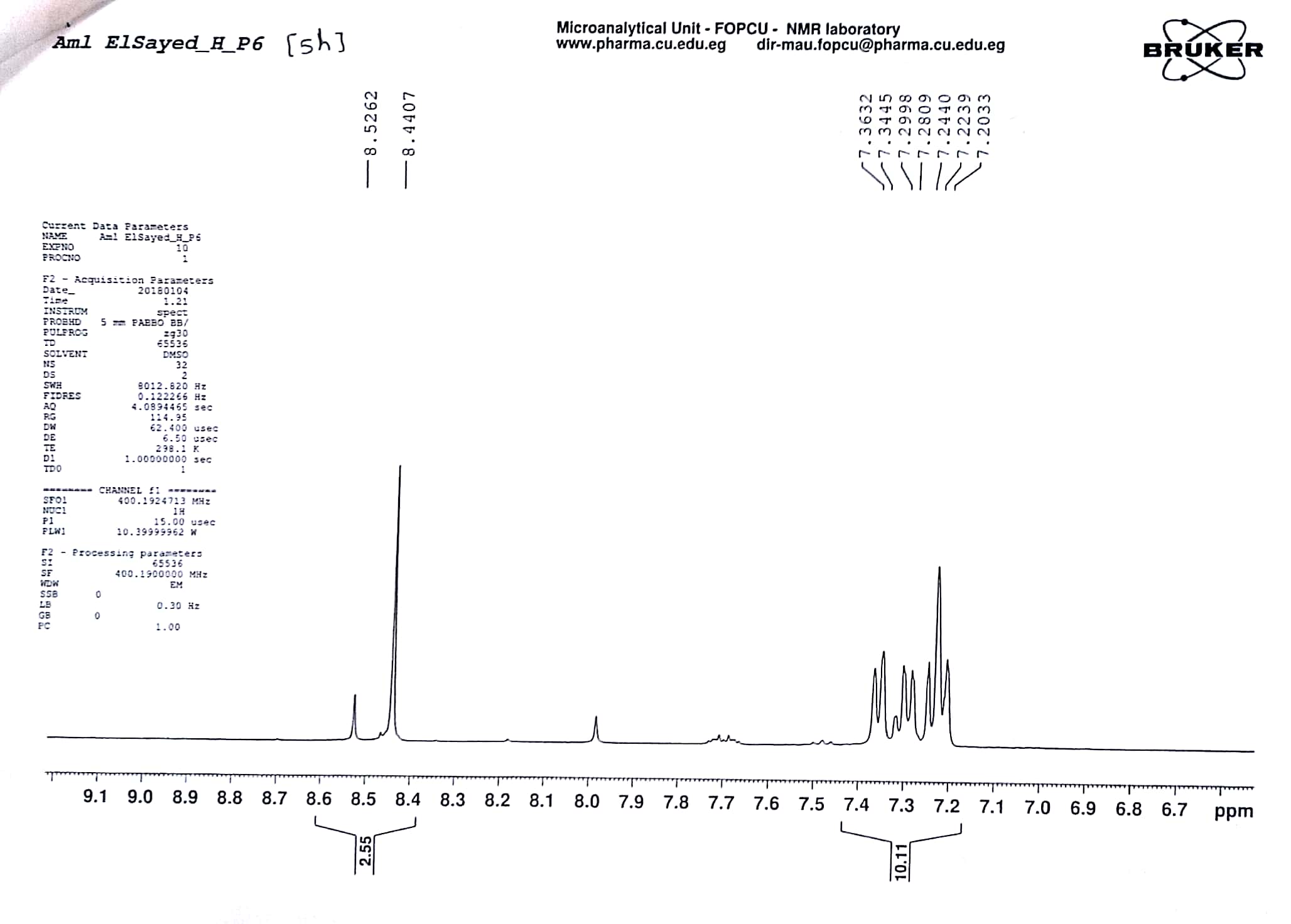

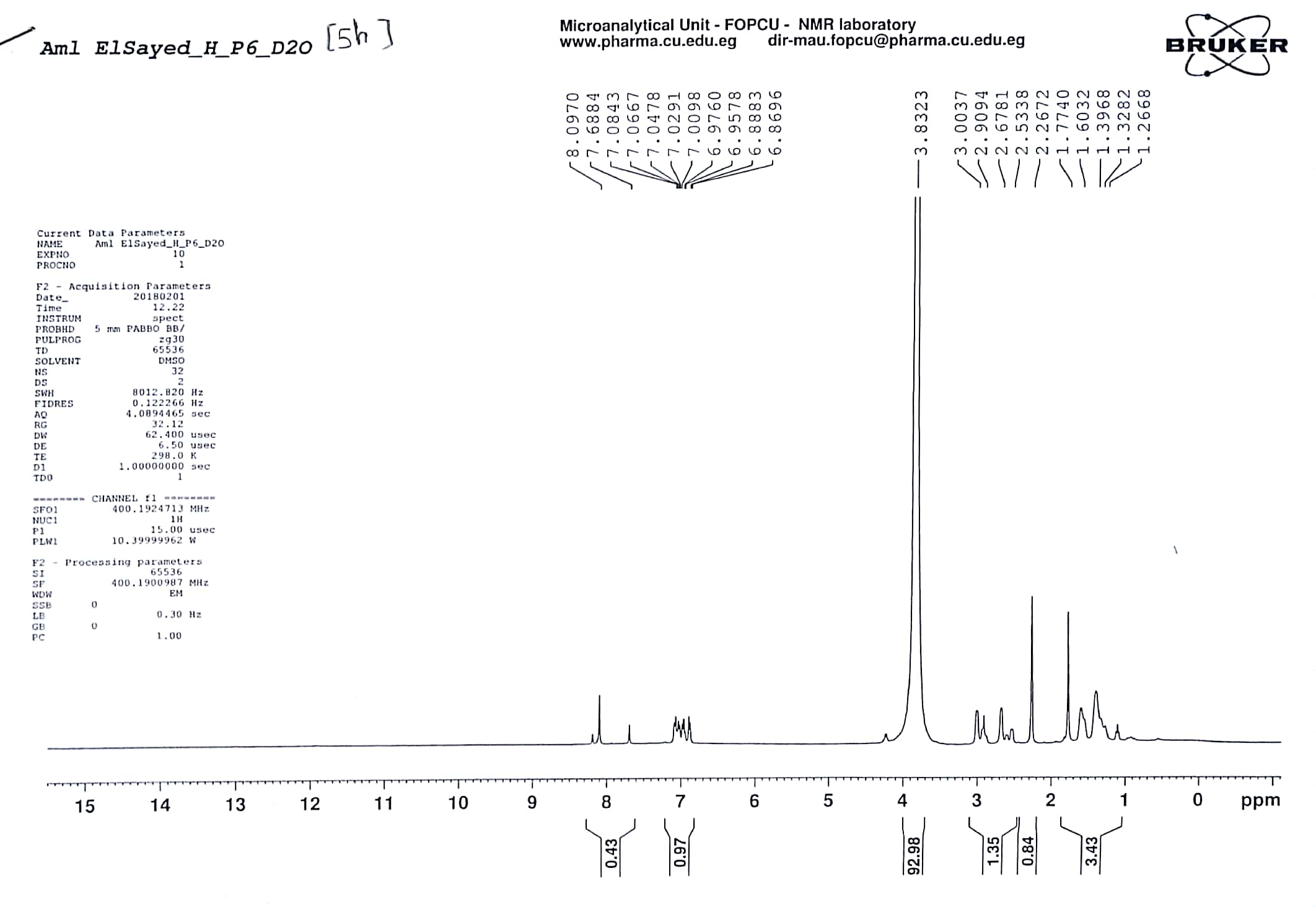


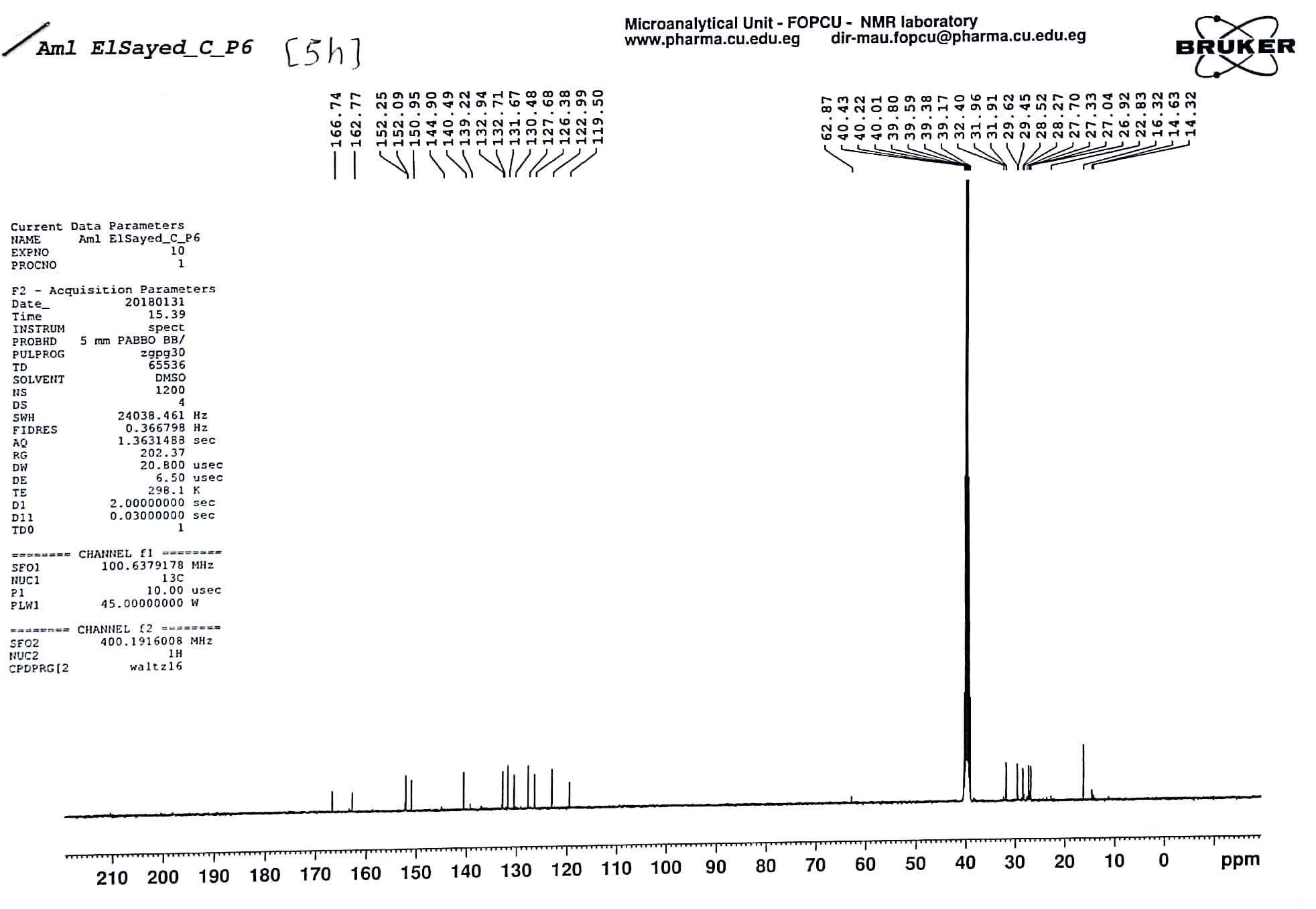

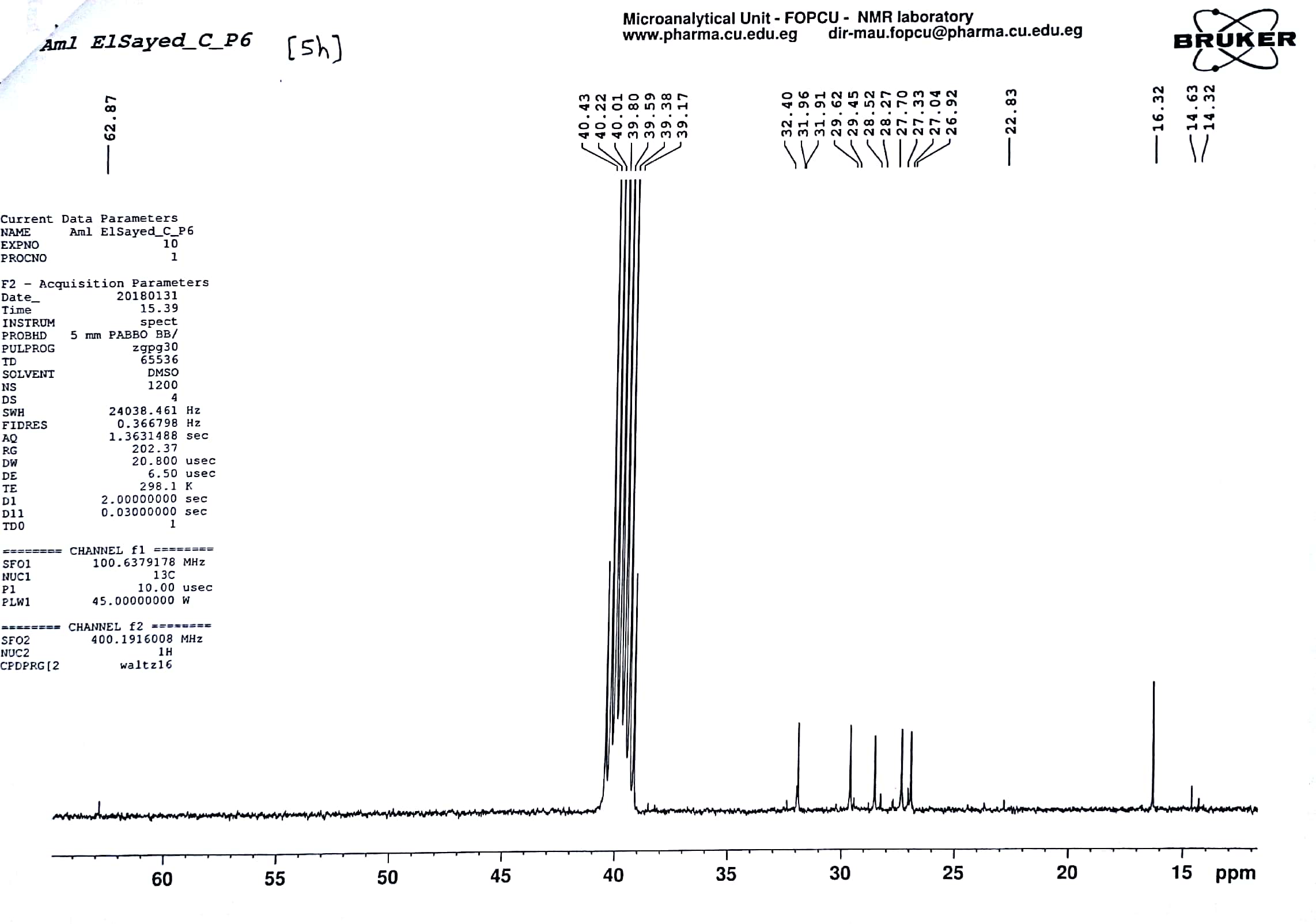

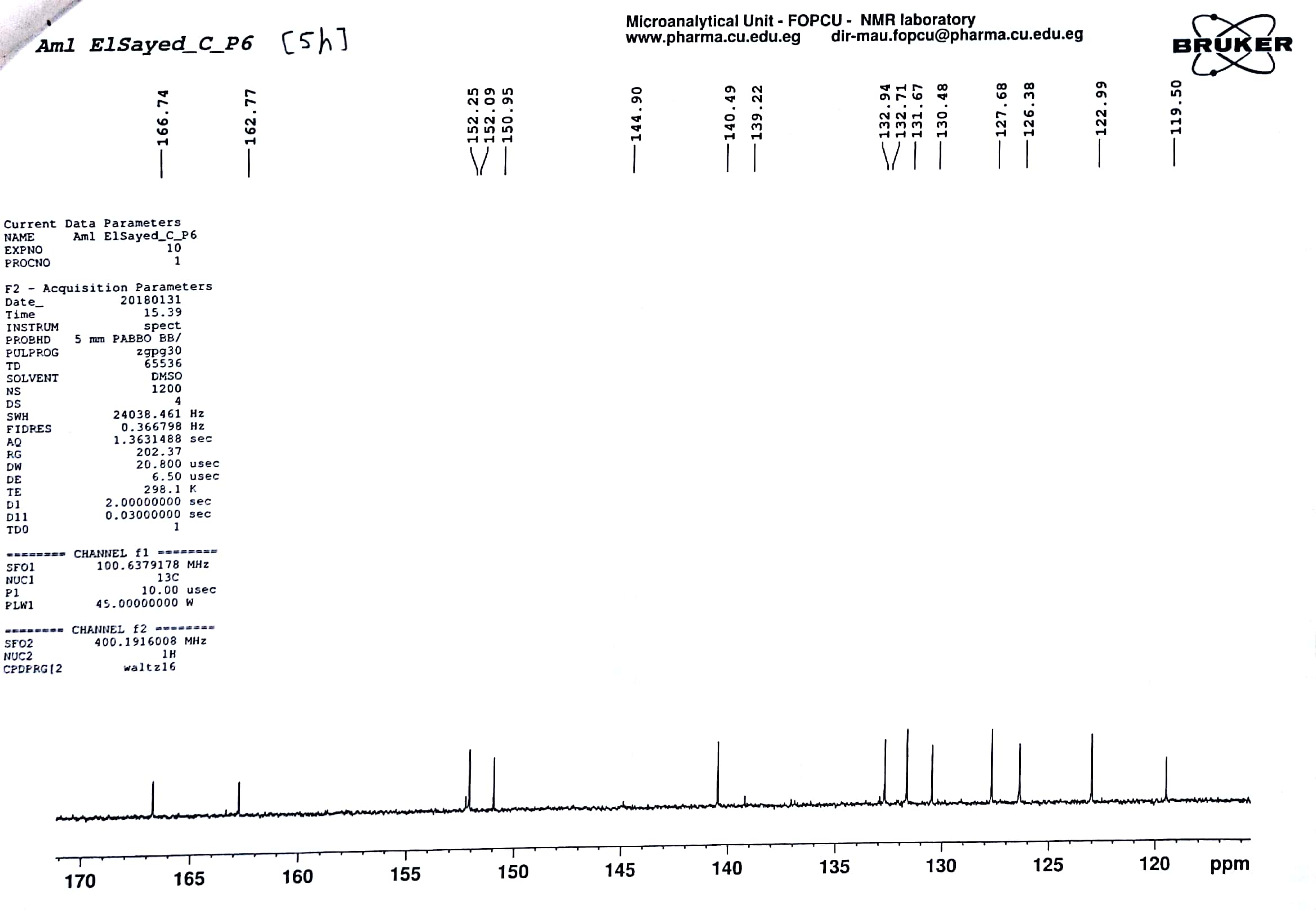

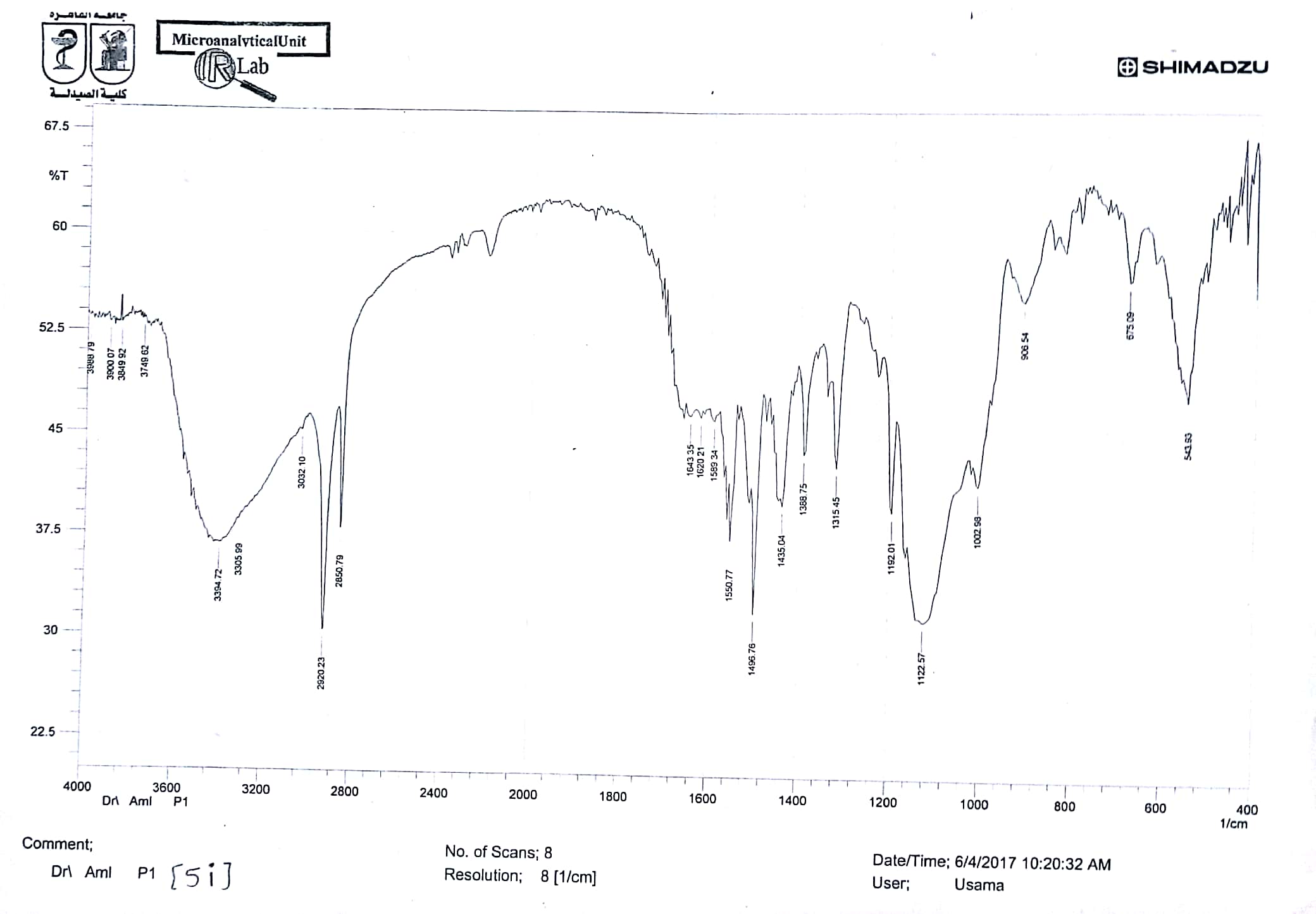

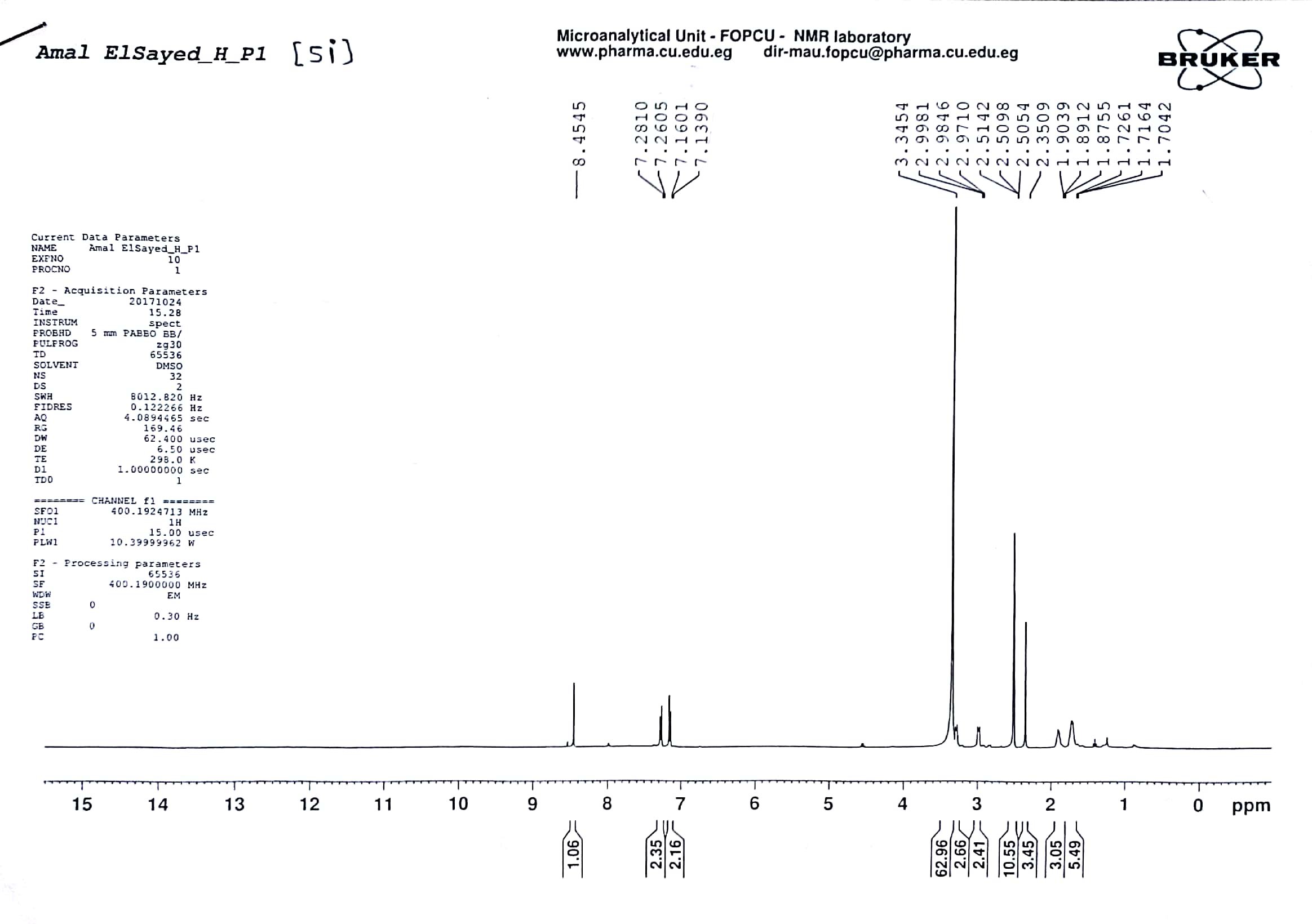

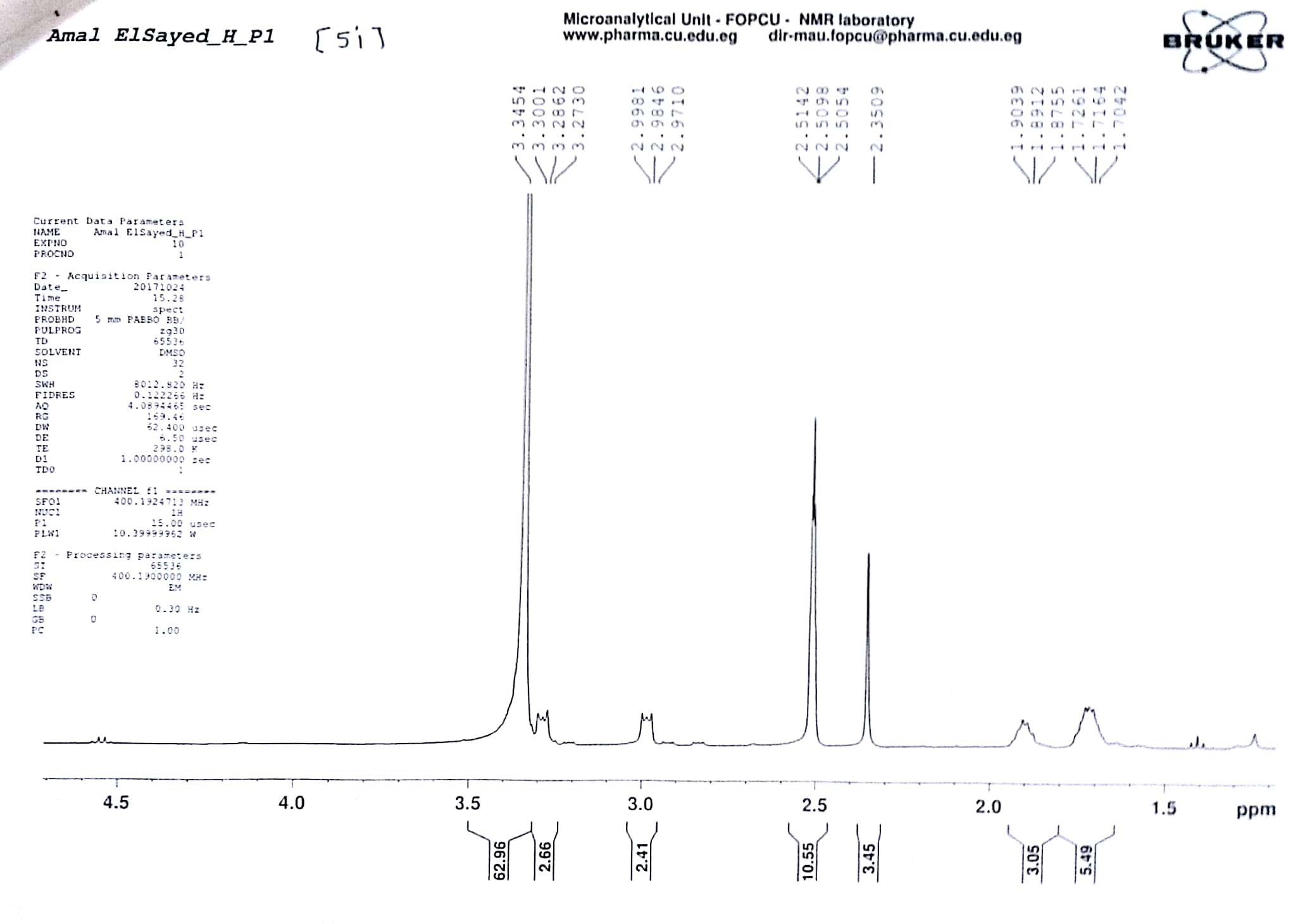

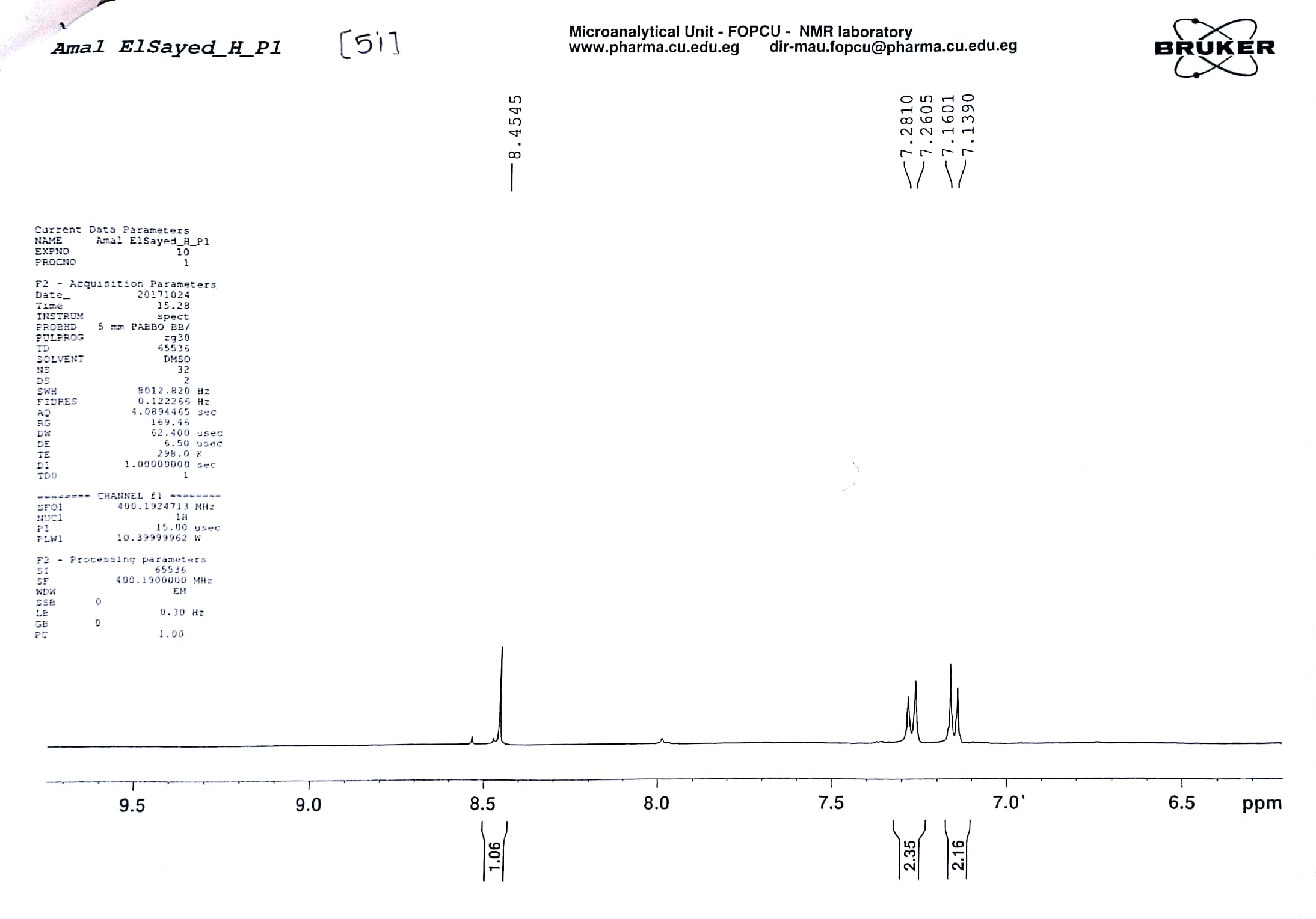

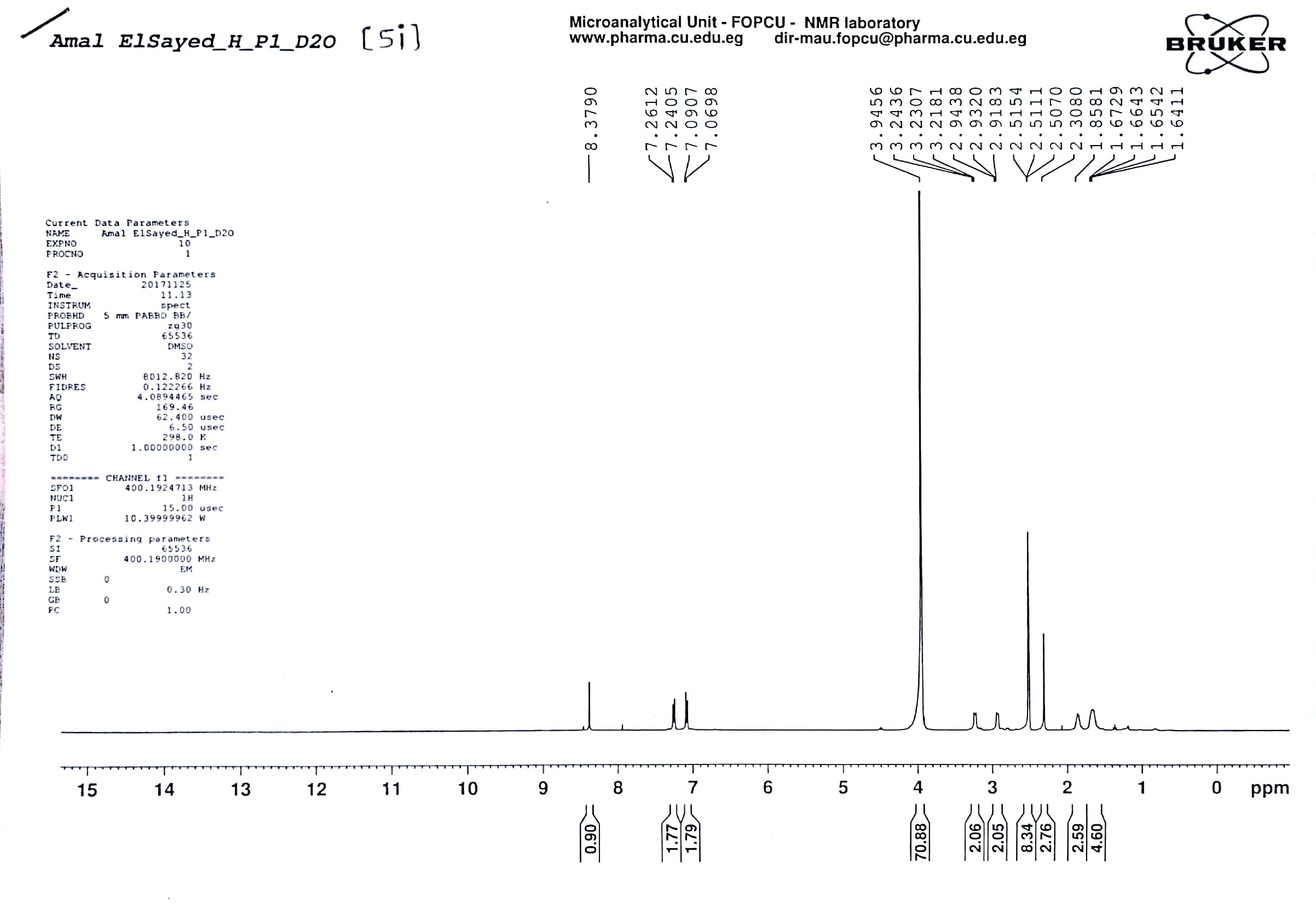


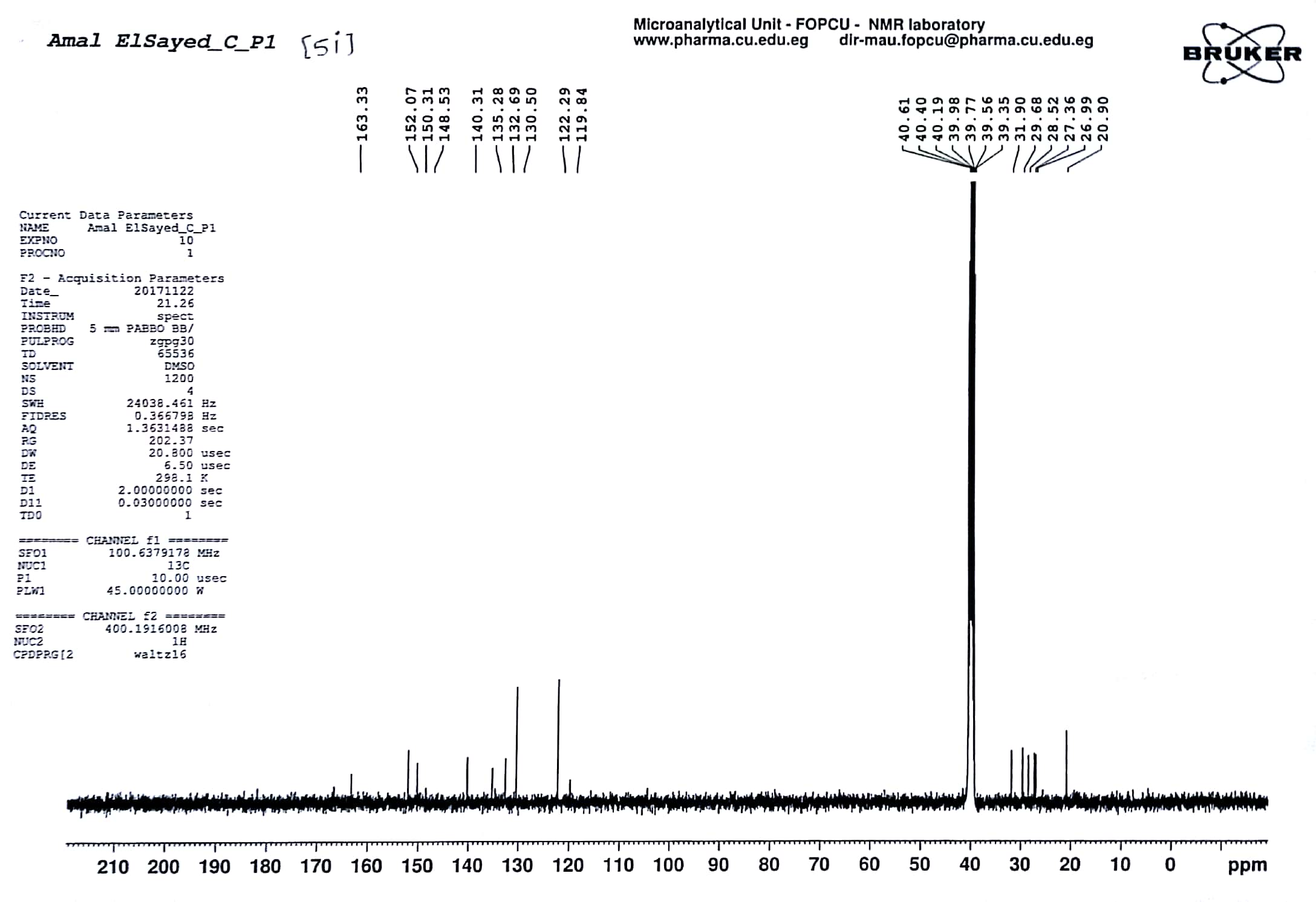


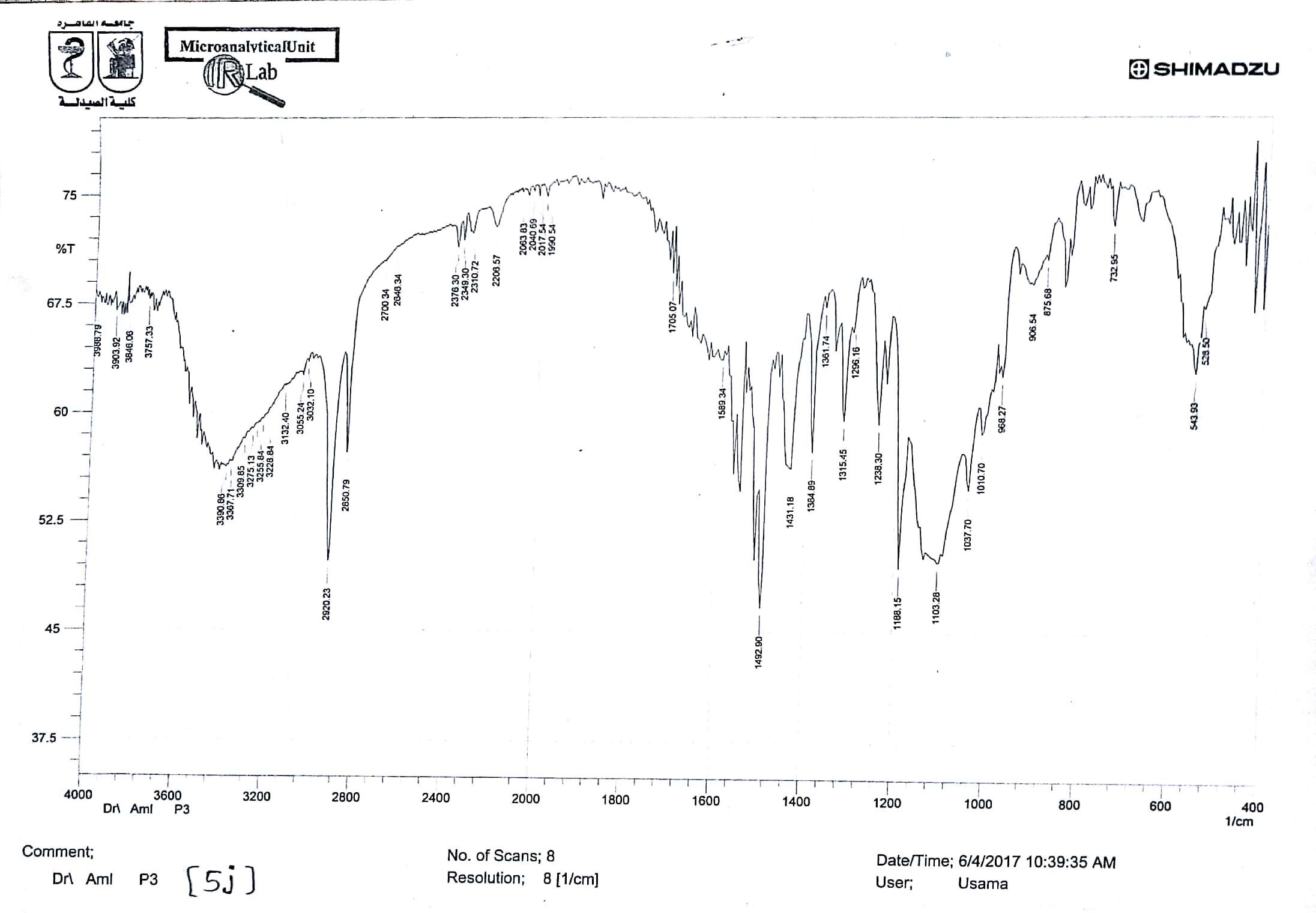

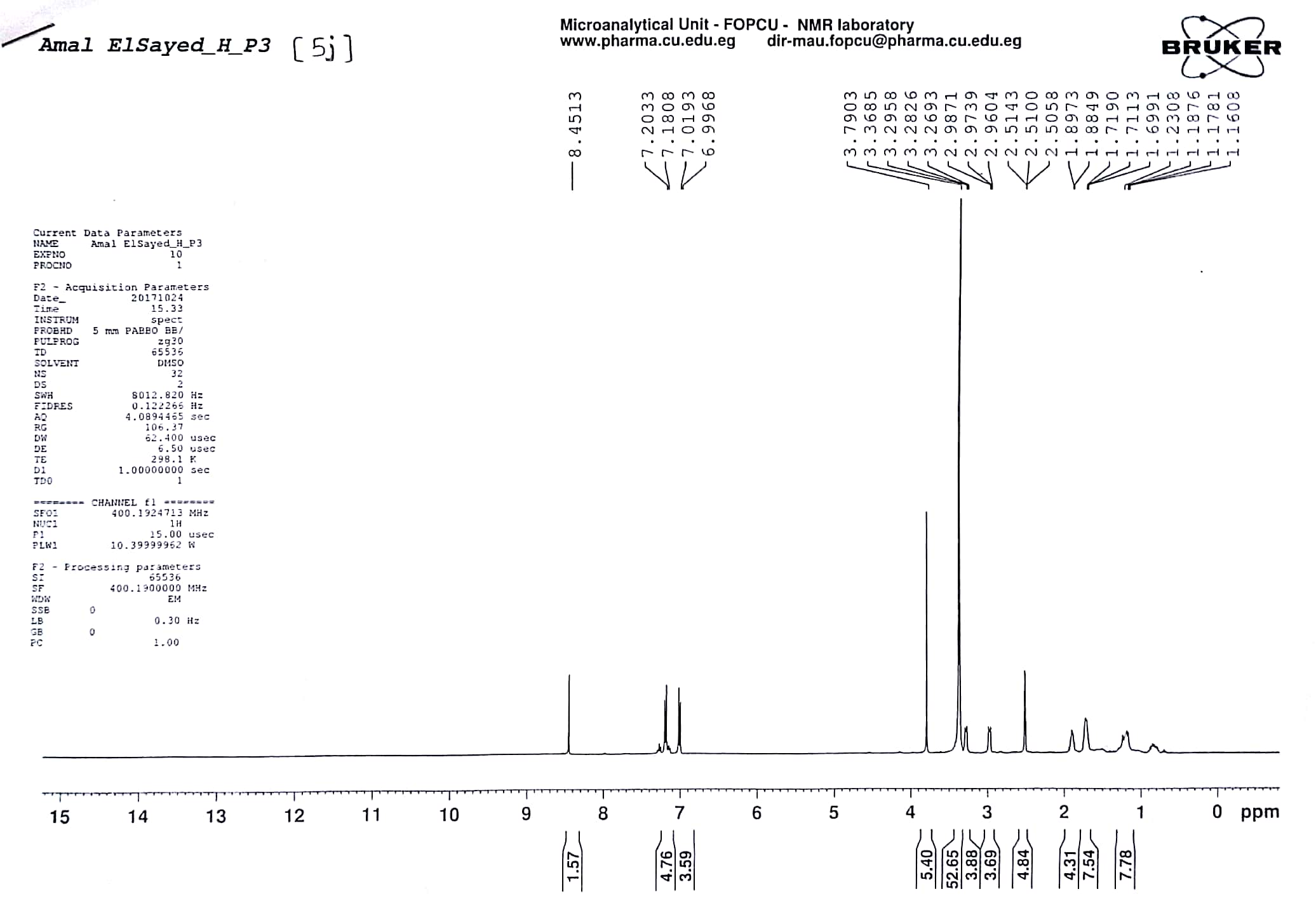

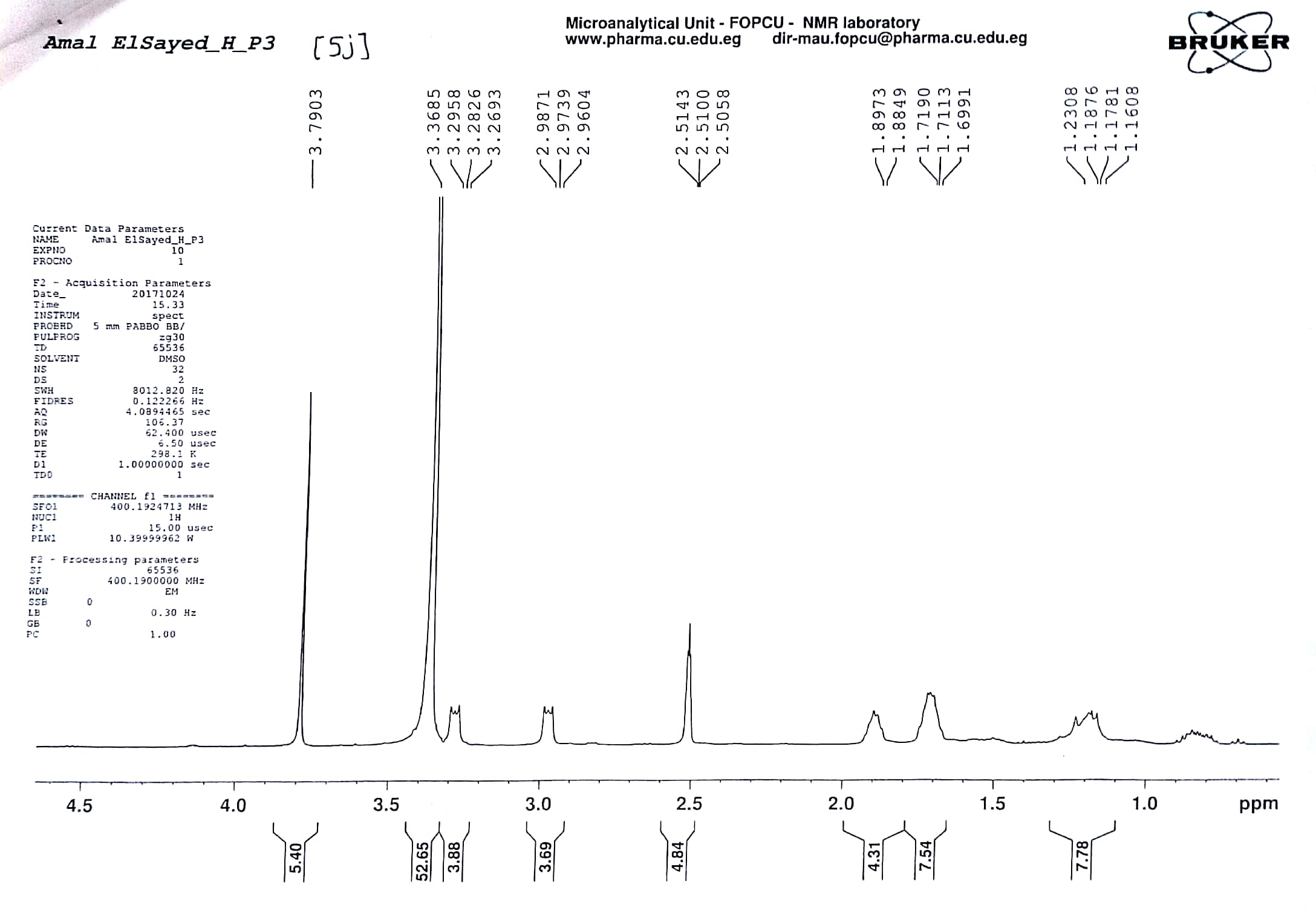

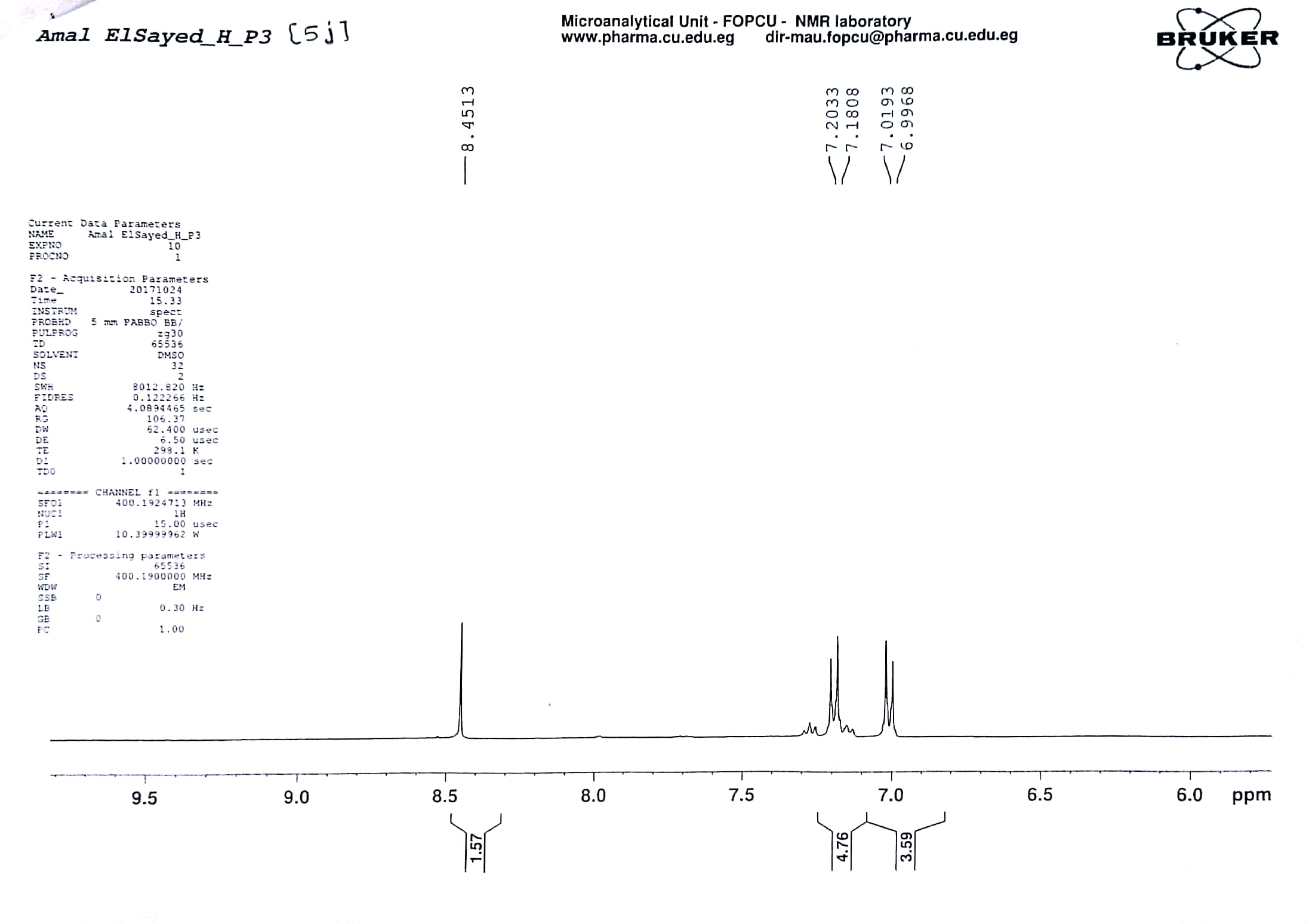

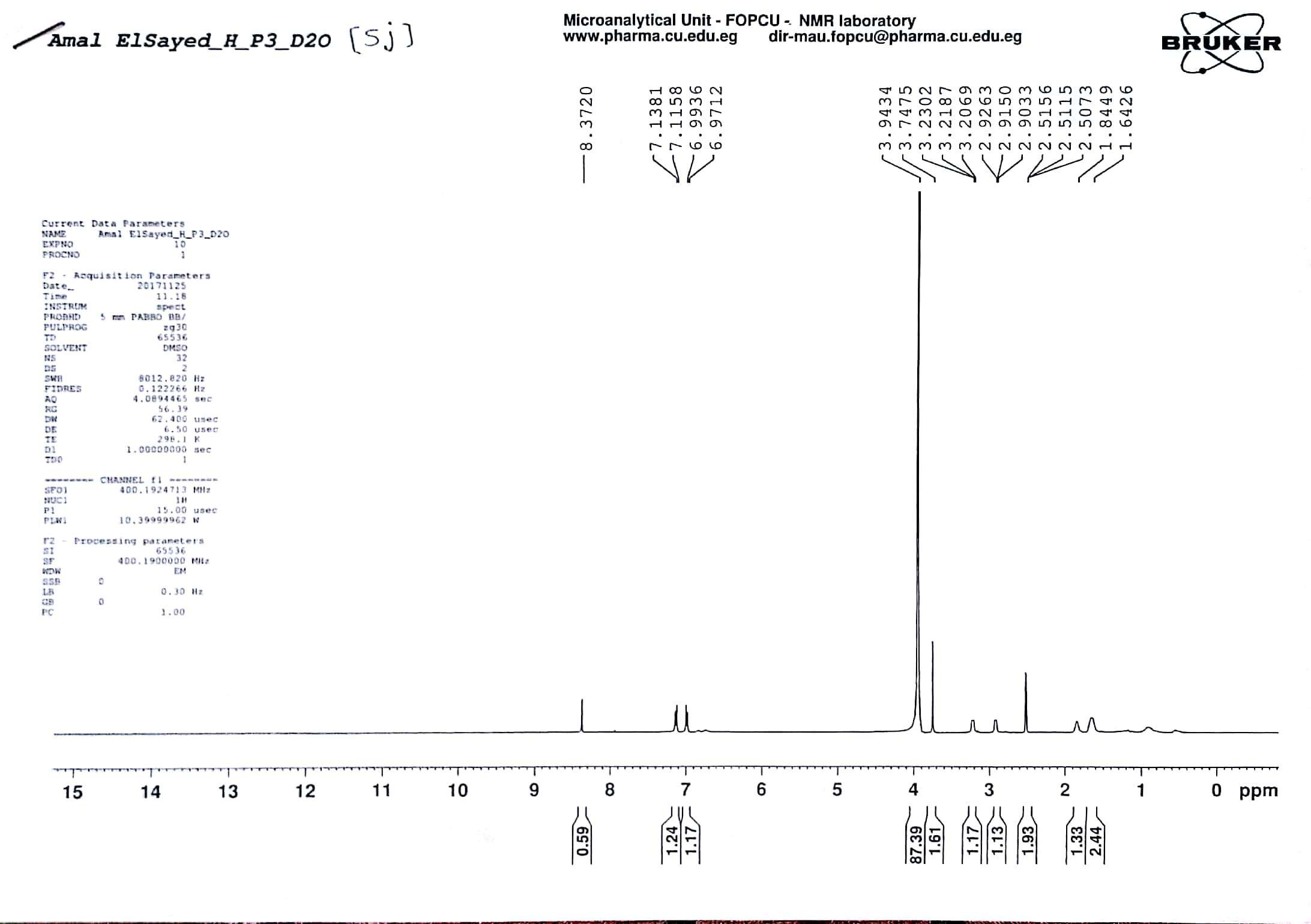

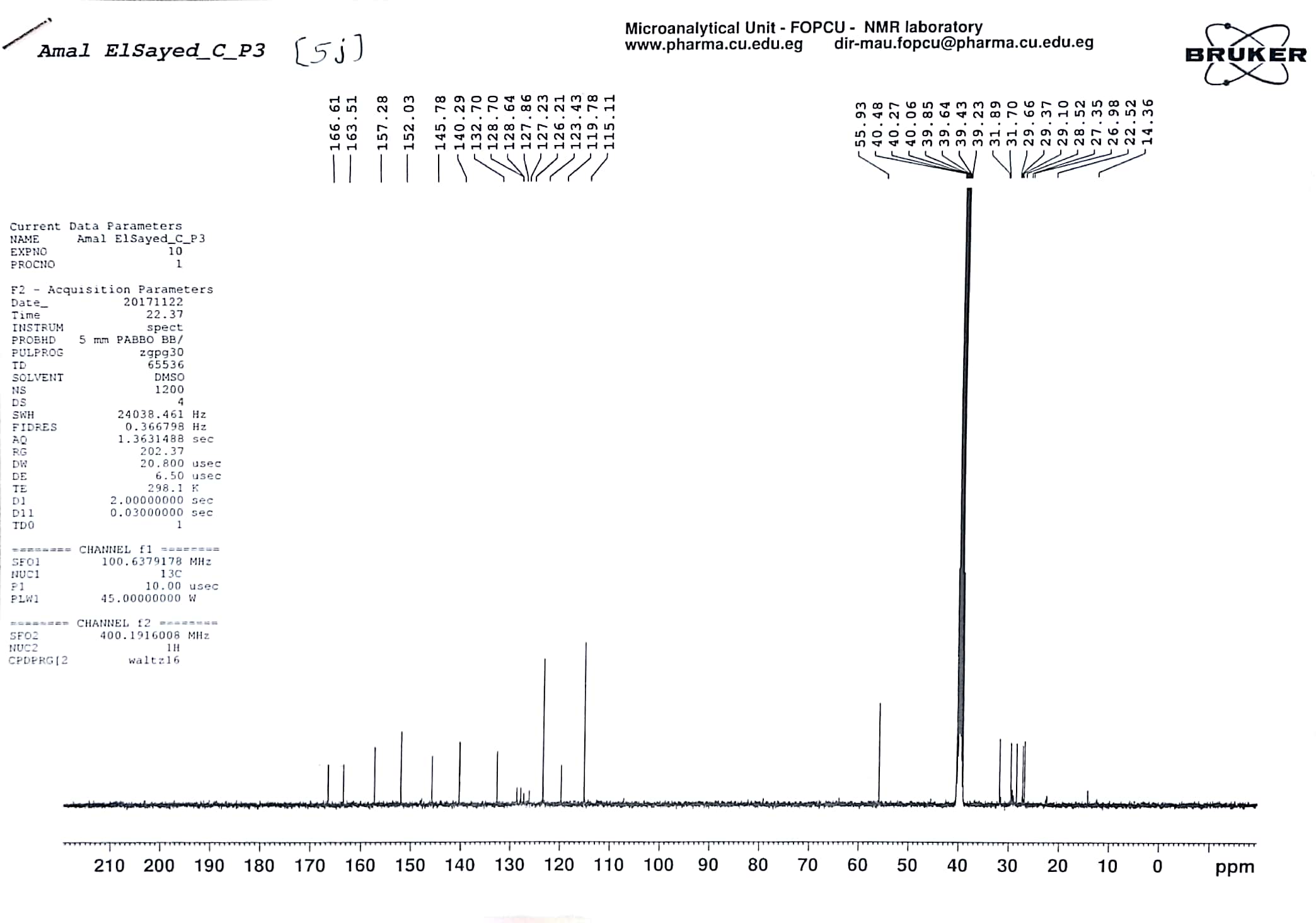

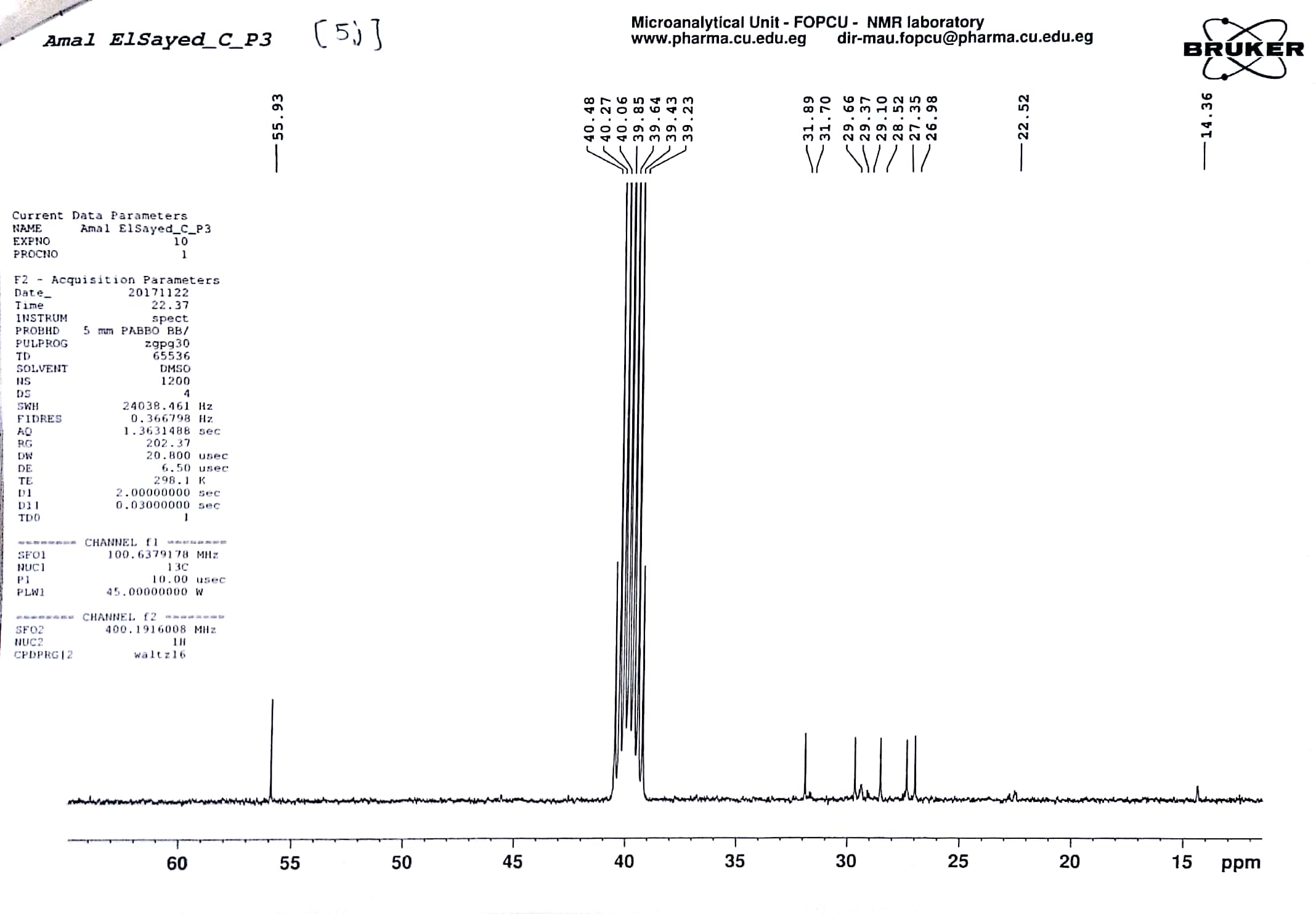

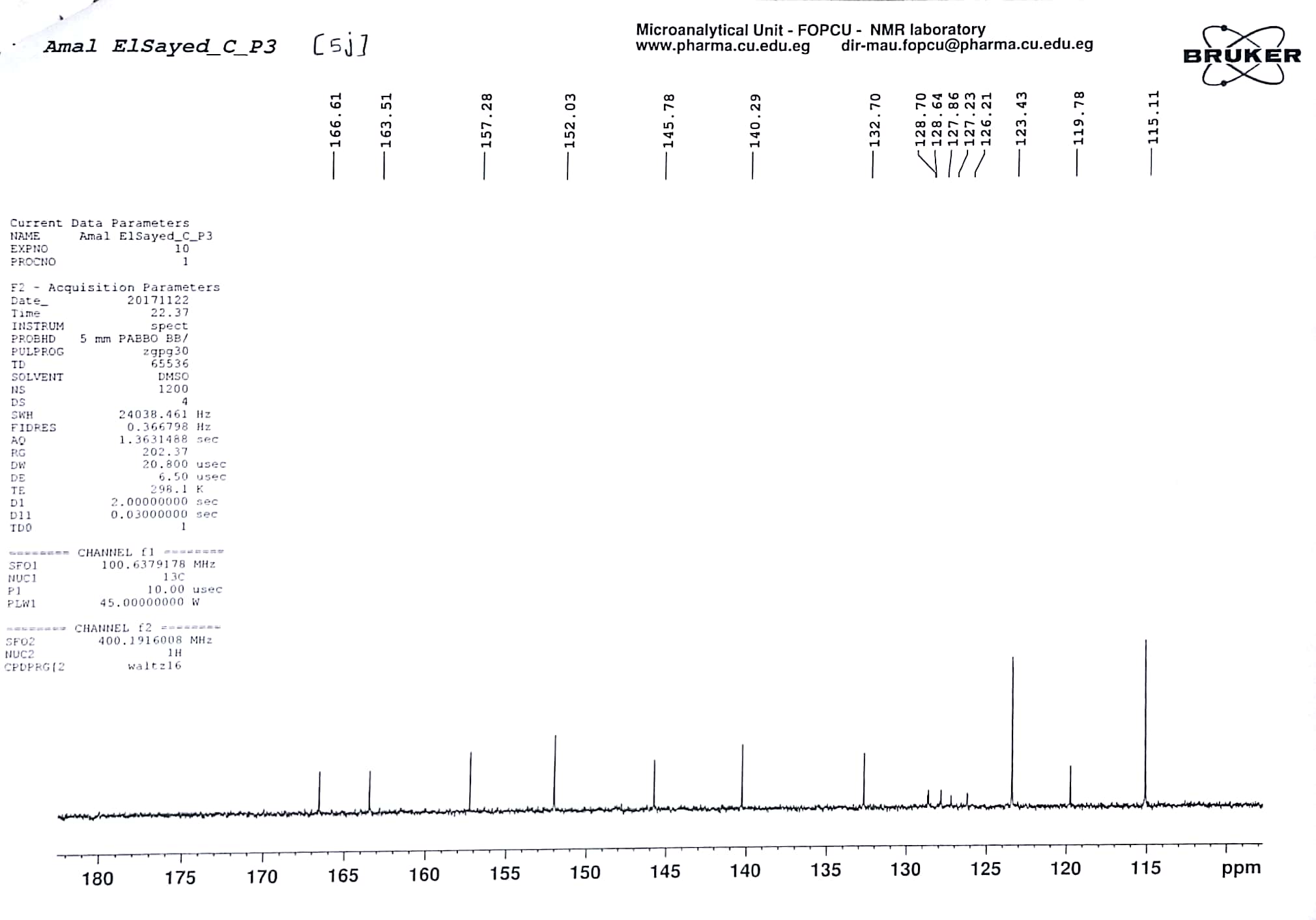

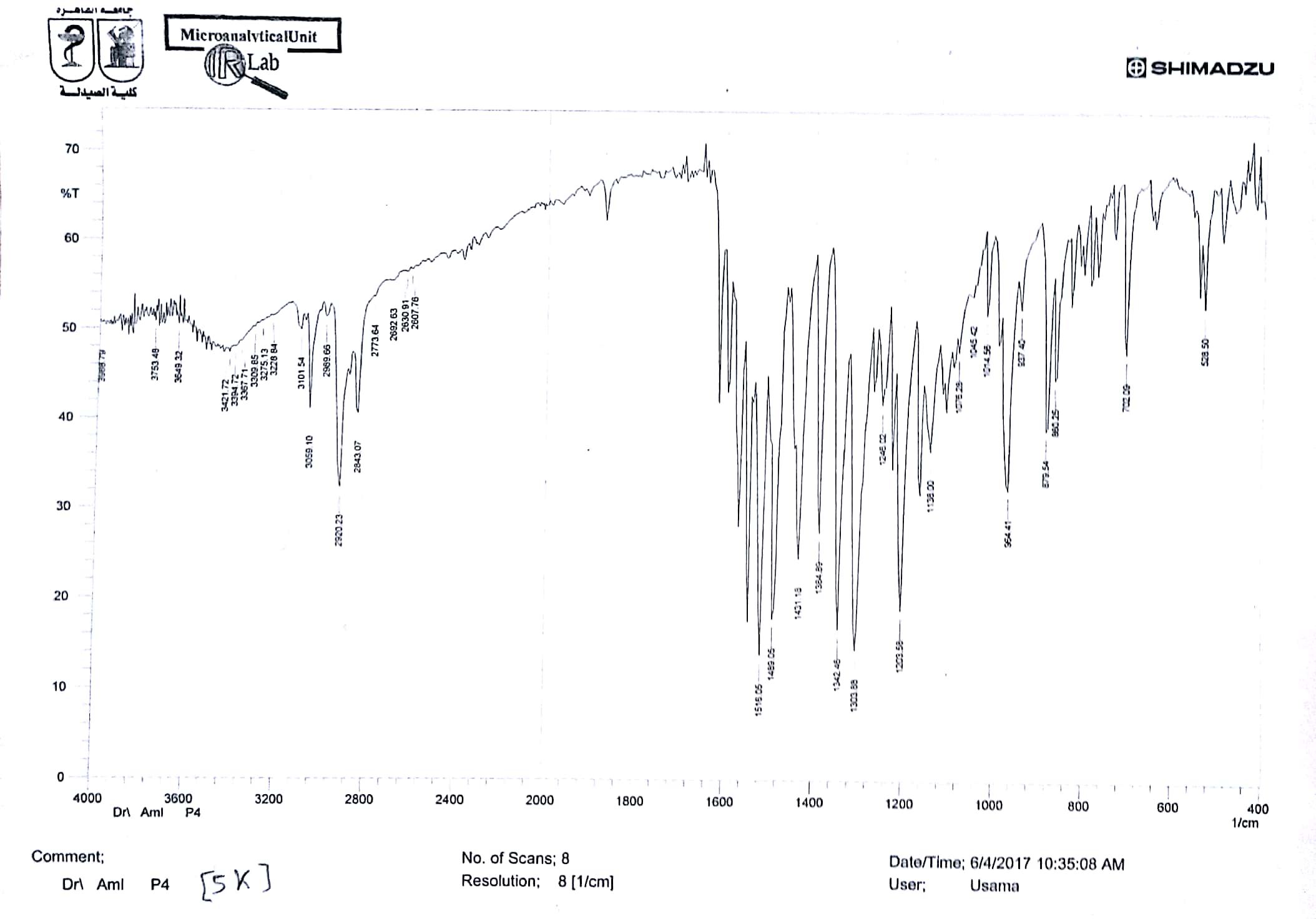

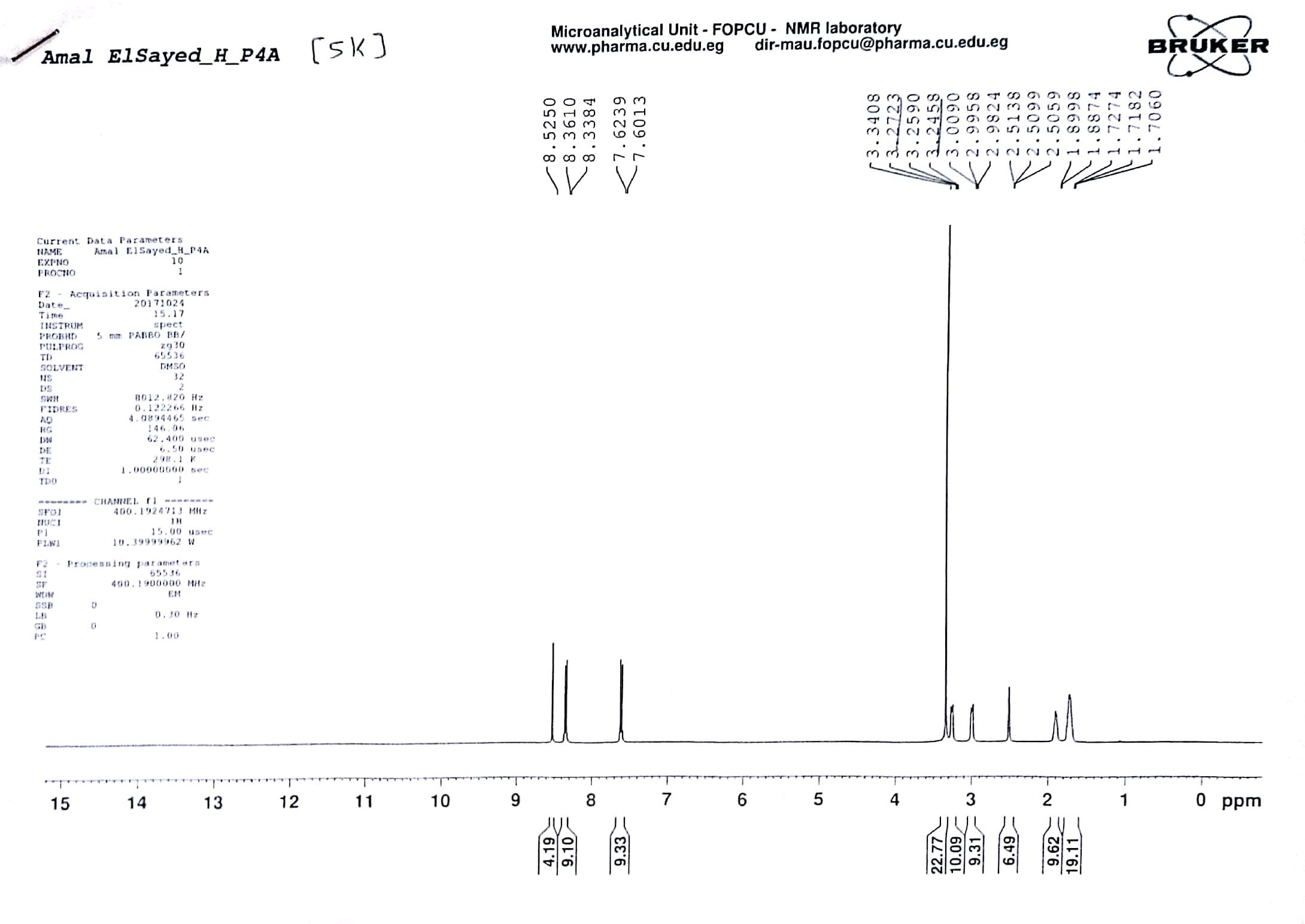

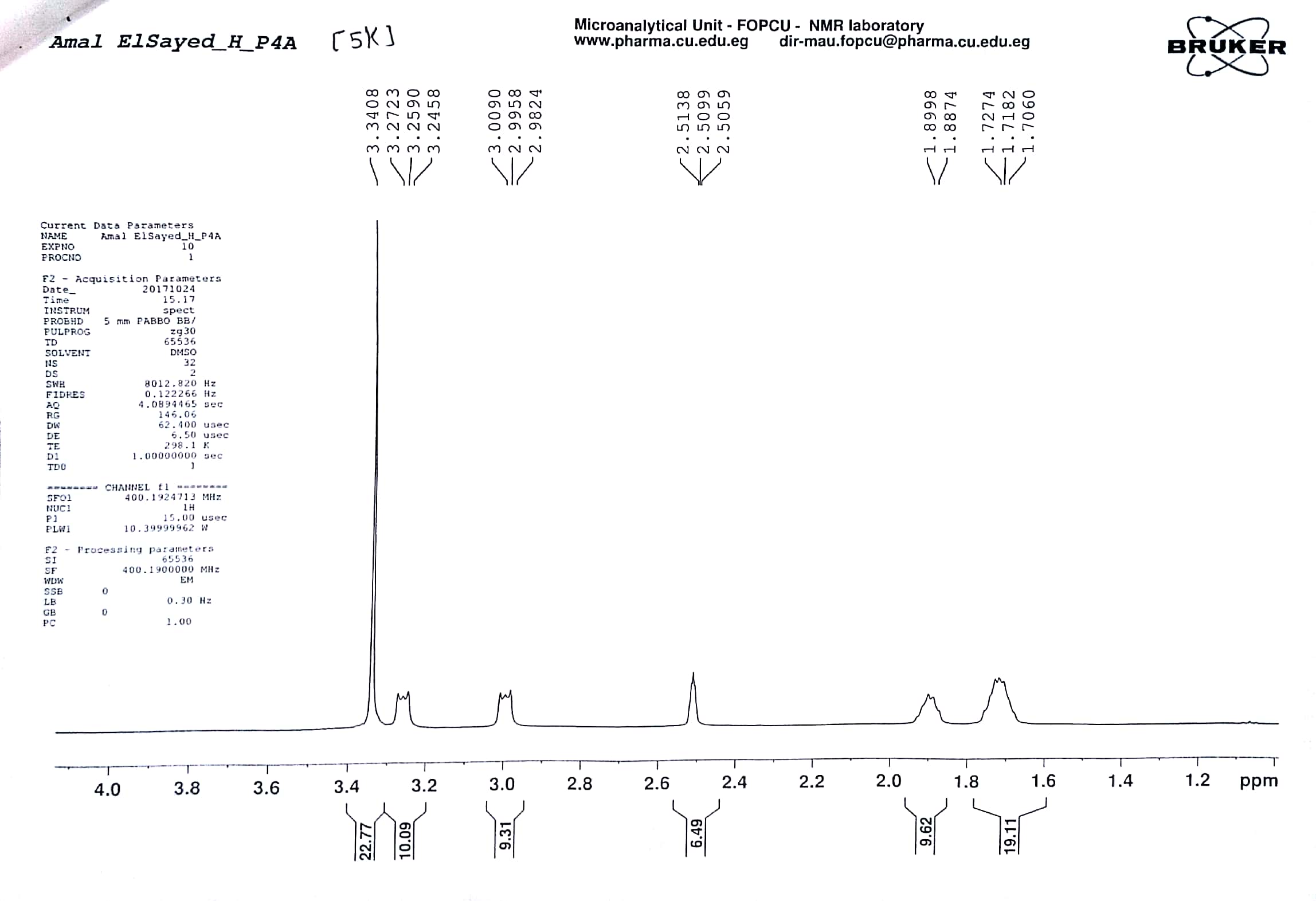

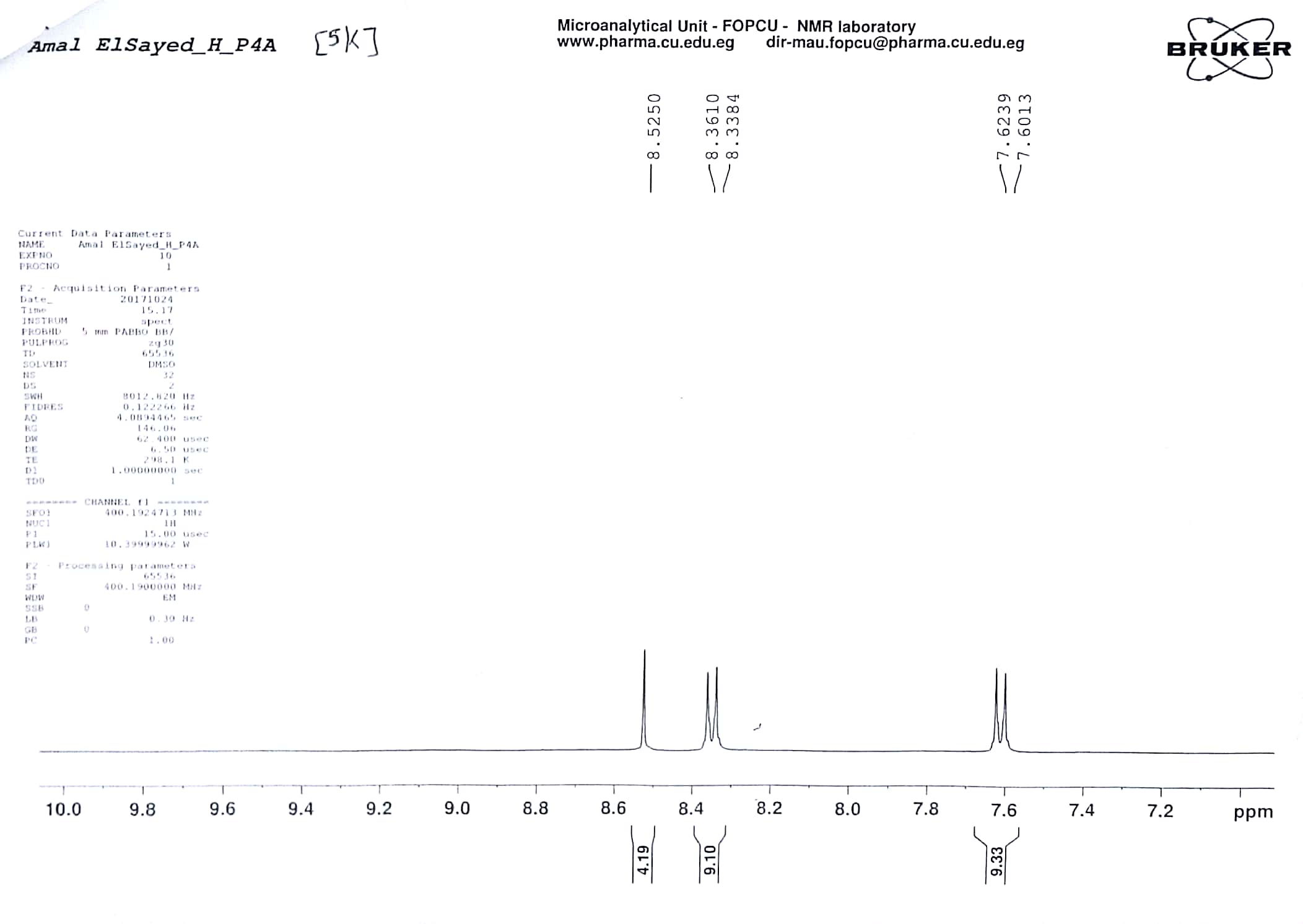

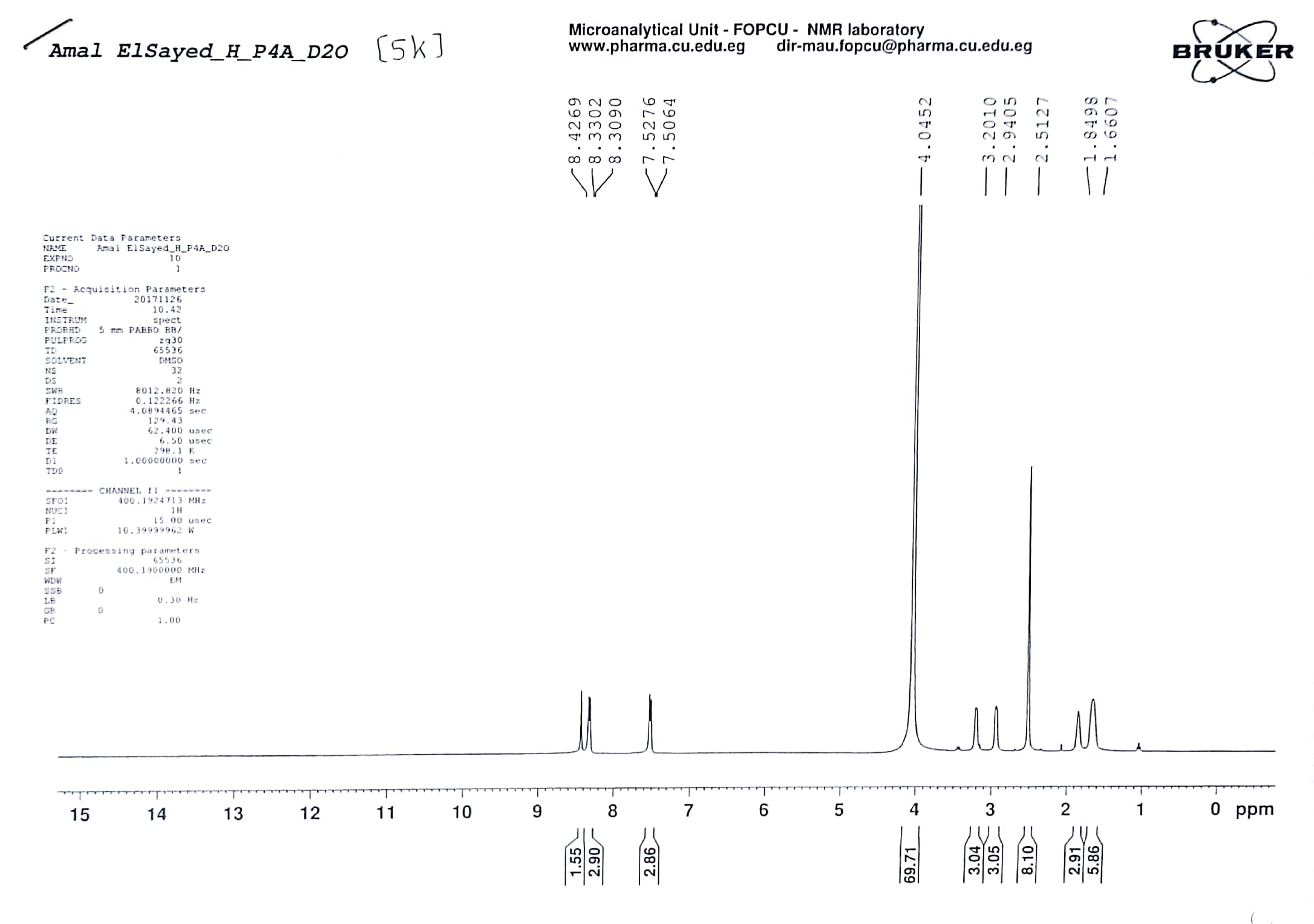

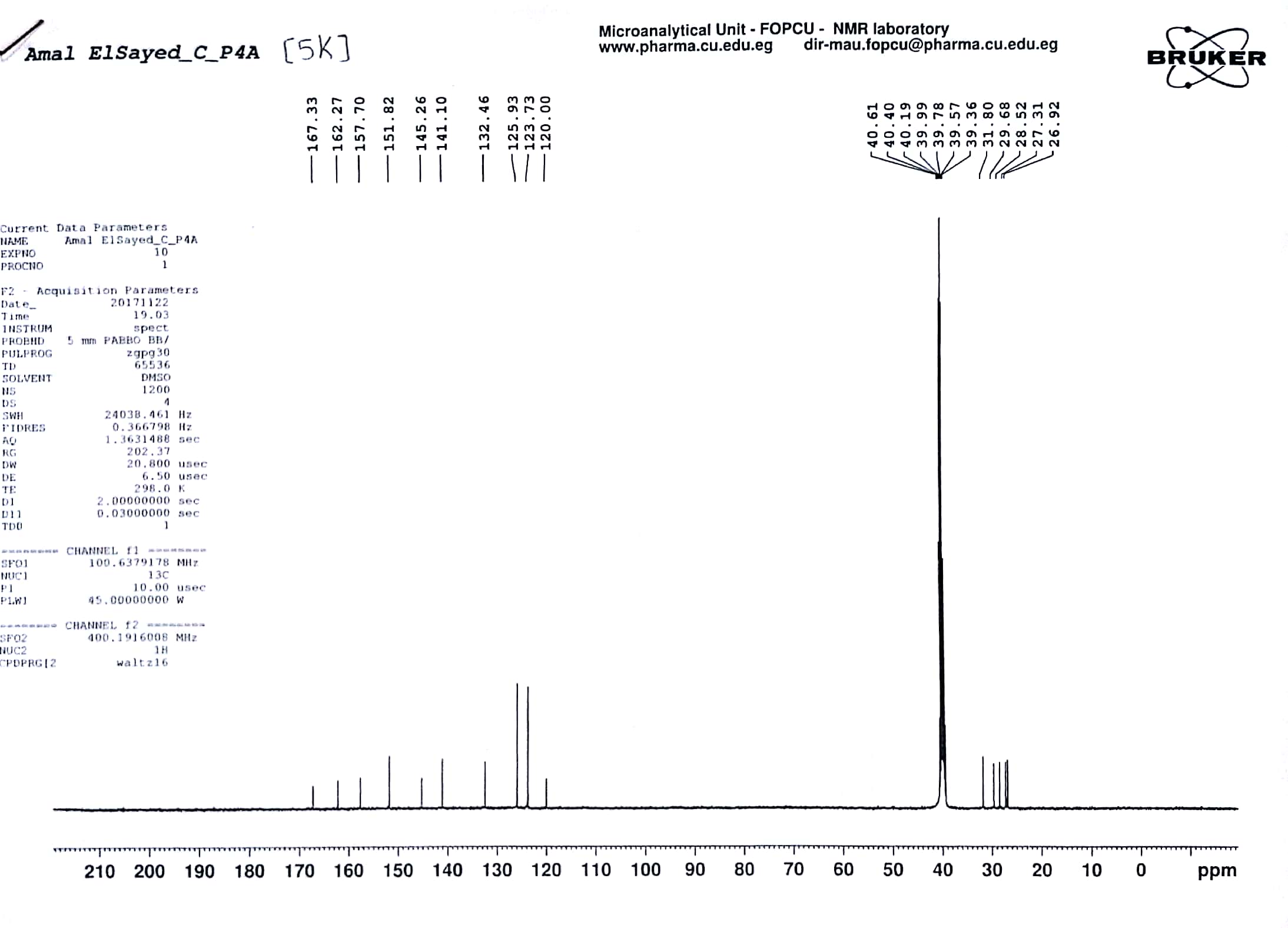

Supplement: Supplemental Material [file IENZ_A_1593160_SM4492.docx]
